# Widespread episodic thiamine deficiency in Northern Hemisphere wildlife

**DOI:** 10.1038/srep38821

**Published:** 2016-12-13

**Authors:** Lennart Balk, Per-Åke Hägerroth, Hanna Gustavsson, Lisa Sigg, Gun Åkerman, Yolanda Ruiz Muñoz, Dale C. Honeyfield, Ulla Tjärnlund, Kenneth Oliveira, Karin Ström, Stephen D. McCormick, Simon Karlsson, Marika Ström, Mathijs van Manen, Anna-Lena Berg, Halldór P. Halldórsson, Jennie Strömquist, Tracy K. Collier, Hans Börjeson, Torsten Mörner, Tomas Hansson

**Affiliations:** 1 Department of Environmental Science and Analytical Chemistry (ACES), Stockholm University, SE-10691 Stockholm, Sweden; 2 Department of Biochemistry, Genetics and Immunology, University of Vigo, Lagoas-Marcosende, ES-36310 Vigo, Spain; 3 Leetown Science Center, Northern Appalachian Research Laboratory, U.S. Geological Survey (USGS), Wellsboro, Pennsylvania 16901, USA; 4 Department of Biology, University of Massachusetts Dartmouth, Dartmouth, Massachusetts 02747, USA; 5 Leetown Science Center, Conte Anadromous Fish Research Laboratory, U.S. Geological Survey (USGS), Turners Falls, Massachusetts 01376, USA; 6 River Ecology and Management, Karlstad University, SE-65188 Karlstad, Sweden; 7 Department of Aquatic Resources, Institute of Freshwater Research, Swedish University of Agricultural Sciences (SLU), SE-17893 Drottningholm, Sweden; 8 Department of Medicine Solna and Center for Molecular Medicine, Karolinska Institutet, SE-17176 Stockholm, Sweden; 9 Institute for Risk Assessment Sciences (IRAS), Utrecht University, NL-3508 TD Utrecht, the Netherlands; 10 Medical Products Agency, Box 26, SE-75103 Uppsala, Sweden; 11 The University of Iceland’s Research Centre in Suðurnes, IS-245 Sandgerði, Iceland; 12 NOAA Fisheries, Northwest Fisheries Science Center, Seattle, Washington 98112, USA; 13 Department of Aquatic Resources, Fisheries Research Station, Swedish University of Agricultural Sciences (SLU), Brobacken, SE-81494 Älvkarleby, Sweden; 14 Department of Disease Control and Epidemiology, National Veterinary Institute (SVA), SE-75189 Uppsala, Sweden

## Abstract

Many wildlife populations are declining at rates higher than can be explained by known threats to biodiversity. Recently, thiamine (vitamin B_1_) deficiency has emerged as a possible contributing cause. Here, thiamine status was systematically investigated in three animal classes: bivalves, ray-finned fishes, and birds. Thiamine diphosphate is required as a cofactor in at least five life-sustaining enzymes that are required for basic cellular metabolism. Analysis of different phosphorylated forms of thiamine, as well as of activities and amount of holoenzyme and apoenzyme forms of thiamine-dependent enzymes, revealed episodically occurring thiamine deficiency in all three animal classes. These biochemical effects were also linked to secondary effects on growth, condition, liver size, blood chemistry and composition, histopathology, swimming behaviour and endurance, parasite infestation, and reproduction. It is unlikely that the thiamine deficiency is caused by impaired phosphorylation within the cells. Rather, the results point towards insufficient amounts of thiamine in the food. By investigating a large geographic area, by extending the focus from lethal to sublethal thiamine deficiency, and by linking biochemical alterations to secondary effects, we demonstrate that the problem of thiamine deficiency is considerably more widespread and severe than previously reported.

Thiamine (vitamin B_1_), an aminopyrimidine ring linked by a methylene bridge to a thiazolium ring, is a water-soluble vitamin needed in all living cells, and as such it is a possible target for noxious influence by environmental disturbances. Inside animal cells, non-phosphorylated thiamine (T) is phosphorylated to thiamine diphosphate (TDP), which functions as a cofactor in at least five life-sustaining enzymes required for basic cellular metabolism, whereas thiamine monophosphate (TMP) is a degradation product, which is recycled or excreted. During the last few decades, severe thiamine deficiency has been observed in wildlife, such as fish^1^, reptiles^2^, and birds^3^. A number of research projects, in both North America and Europe, have investigated the problem and tried to find the underlying cause^4^,^5^,^6^. So far, however, no general cause has been found. The purpose of the present investigation was to provide a better basis for a biochemical understanding of the problem. We also found that thiamine deficiency in aquatic wildlife was much more widespread than previously reported, both geographically and among taxa—a finding that may be related to recently observed thiamine depletion in the aquatic environment^7^. Thiamine deficiency is ultimately lethal, but it also has a number of preceding sublethal health effects, such as memory and learning disorders, immunosuppression, damage to the blood-brain barrier, neurological disorders, reduced food intake, and altered carbohydrate, protein, and lipid metabolism^8^,^9^,^10^,^11^. Thiamine deficiency was systematically investigated in seven feral species belonging to three animal classes: bivalves, ray-finned fishes, and birds. The investigation covered 45 stations in 15 regions in the Northern Hemisphere (Fig. 1, Fig. S1a–o, Table S1).

When working with feral animals in the field, the occurrence of thiamine deficiency may be demonstrated in several ways, not only by comparison of central tendency with non-thiamine-deficient (control) specimens: 1) control values may be known from the literature, as *e.g.* in the case of latency (percentage apoenzyme, *i.e.* the degree of missing cofactor) of thiamine-dependent enzymes; 2) the range of observed values, of *e.g.* thiamine concentrations and enzyme activities, is usually larger in a group of specimens with various degree of thiamine deficiency than in a group where all specimens are non-thiamine-deficient; 3) remediation of specimens by thiamine treatment proves that the specimens were thiamine-deficient before the treatment; and 4) typical clinical symptoms associated with subsequent mortality are indicative of thiamine deficiency. We employed all of these approaches to demonstrate thiamine deficiency, and their rationale is described in more detail in the Methods. The thiamine deficiency biomarker responses in the feral species were confirmed in laboratory experiments with domestic chicken (*Gallus gallus*) and blue mussel (*Mytilus sp*.). The primary biochemical effects of the thiamine deficiency were also linked to a number of secondary effects, such as reduced swimming endurance, impaired growth, lowered body condition, reduced reproductive outcome, parasite infestation, and altered blood chemistry and composition. Many previous investigations, especially in salmonines (Salmoninae, a subfamily within Salmonidae), have focused mainly on the relationship between thiamine deficiency and mortality, without consideration of sublethal effects, which occur at substantially higher thiamine concentrations than the reduced levels of thiamine associated with direct mortality. A growing awareness of the importance of sublethal thiamine deficiency led us to revisit previously published data on thiamine concentrations in salmonine eggs in order to find the threshold for sublethal thiamine deficiency. Because thiamine deficiency obviously has a strong negative impact on both reproduction and survival, our hypothesis, that thiamine deficiency may be a significant contributor to population declines in many ecosystems in the Northern Hemisphere, was strongly supported.

## Results

### Blue mussel (*Mytilus sp*.)

**Figure 2:**
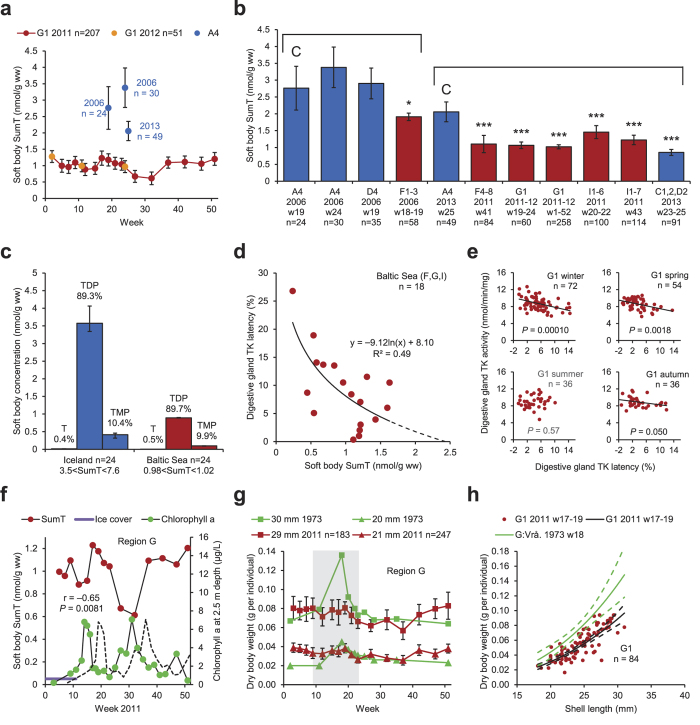
Blue mussel (*Mytilus sp*.). (**a**) Soft body SumT at G1 in the County of Södermanland (red or orange) and at A4 in Iceland (blue). (**b**) Soft body SumT in Iceland (blue) and the Baltic Sea area (red). ANOVA-type regression model. (**c**) Concentration and proportion soft body T, TDP, and TMP in Icelandic (blue) specimens with high SumT and Baltic Sea (red) specimens with low SumT. (**d**) Digestive gland TK latency and soft body (except digestive gland) SumT in the Baltic Sea. (**e**) Digestive gland TK activity and latency at G1 during four seasons. (**f**) Soft body SumT at G1 (red) and chlorophyll *a* in the water at G2 (green) in the County of Södermanland. Dashed line: five weeks delayed chlorophyll *a* curve. (**g**,**h**) Dry body weight in specimens of different lengths at G1 2011 (red/black, this investigation) and Vrångskär in the County of Södermanland 1973 (green, adapted from Kautsky^16^). (**g**) The shaded area indicates the reproductive period (March 1–June 10) defined by Kautsky^16^. (**h**) Week 17–19. Dashed lines: 95% CI. • Bars: arithmetic means. Whiskers: 95% CI. C: control. Group comparisons are indicated by brackets.

A one and a half year long baseline investigation with regular sampling of blue mussels was performed at G1 in the Baltic Sea. SumT (T+TDP+TMP) in the G1 blue mussels was generally low (1.0±0.1 nmol/g) compared with A4 in Iceland (Fig. 2a,b). Low SumT levels, similar to those at G1, were found also in other parts of the Baltic Sea (Fig. 2b). Obviously, the investigated Baltic Sea blue mussels suffered from severe thiamine deficiency. There was also significant geographic and temporal variation in SumT (Fig. 2a,b), indicating that the thiamine deficiency occurs episodically. The data in Fig. 2b do not, however, warrant conclusions about trends over the years. The even SumT level in the groups with lowest SumT 2011–2013 (Fig. 2b) may indicate a threshold for survival of ca 1.0 nmol/g. It is important that SumT concentrations as high as 3.4 nmol/g do occur (Fig. 2a,b), and may be indicative of a more normal thiamine status. The proportions of T, TDP, and TMP were very similar in specimens from Iceland and the Baltic Sea differing in SumT levels (Fig. 2c, Fig. S2a). This finding suggests that the cause of the thiamine deficiency is not malfunction of the thiamine phosphorylation within the cells. As expected, but not previously shown in blue mussels, digestive gland transketolase (TK) activity was positively related to soft body SumT in a subsample from the Baltic Sea (Fig. S2c), whereas digestive gland TK latency (proportion of apoenzyme) was negatively related to soft body SumT in the same material (Fig. 2d). A negative relationship was also demonstrated between digestive gland TK latency and digestive gland SumT in a number of aquaculture blue mussels experimentally subjected to hypoxia and hyperthermia (Fig. S2d). These relationships demonstrate that TK activity and latency are relevant biomarkers of thiamine deficiency also in this invertebrate species. Negative relationships between digestive gland TK activity and latency were observable at A4 (Fig. S2e) and in three of four seasons at G1 (Fig. 2e).

In the aquatic food web, thiamine is produced mainly by micro and macro algae, and to a more limited extent by fungi and bacteria^12^,^13^,^14^,^15^. Hence, SumT concentrations in the blue mussels are expected to be positively correlated with chlorophyll *a* in the water, but we found a negative correlation between blue mussel SumT and water chlorophyll *a* (Fig. 2f). This finding indicates a disturbed production and/or flux of thiamine in the aquatic food web today. If the chlorophyll *a* curve was delayed five weeks, however, there was a tendency towards a positive correlation (Fig. 2f, dashed line). An explanation of this observation could be that thiamine in the blue mussels comes mainly from fungi and bacteria feeding on settling dead phytoplankton some time after the blooms.

In Fig. 2g,h and Fig. S2i, dry body weight data on the blue mussels from G1 2011–2012 are compared with corresponding data from a nearby (ca 12 km) station in region G in 1973 presented by Kautsky^16^. The most notable observation was the almost complete absence of a peak in the dry body weight during the reproductive period in 2011 (Fig. 2g, shaded area). This difference was also reflected in Fig. 2h, illustrating the respective dry body weights in week 17–19, which was the time of the peak in the dry body weight in 1973. Lower dry body weight during the reproductive period was also observed 2011–2012 compared with corresponding data from south-western Finland in 1992 (Fig. S2j) presented by Öst & Kilpi^17^. These findings suggest impaired reproduction in the blue mussels today, possibly as a result of thiamine deficiency. During the rest of the year (outside the reproductive period) the dry body weight was instead higher 2011–2012 than in 1973 (Fig. S2i). Explanations of this observation could be a trade-off between somatic growth and gonadal growth, or a generally higher availability of food for the blue mussels today due to eutrophication. Important, though, is that the blue mussels do not have a generally lower dry body weight today than 40 years ago. This finding speaks against the hypothesis that the recent population declines in several diving duck species (including the common eider) feeding on blue mussels in the Baltic Sea^18^ would be caused by generally lower flesh content in the blue mussels.

### Common eider (*Somateria mollissima*)

**Figure 3:**
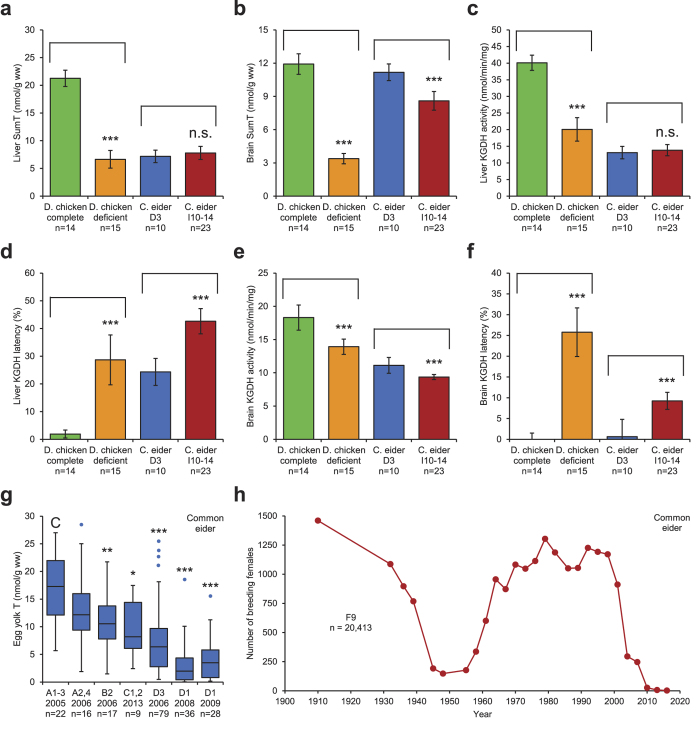
Common eider (*Somateria mollissima*) and domestic chicken (*Gallus gallus*). (**a*****–*****f**) Domestic chicken thiamine “complete” group (green, 17.5 nmol T per g dry fodder) and thiamine “deficient” group (orange, 1.2 nmol T per g dry fodder), common eider females at D3 in eastern Iceland (blue) and at I10–14 in the County of Blekinge (red). Student’s *t*-test. (**a**) Liver SumT. (**b**) Brain SumT. (**c**) Liver KGDH activity. (**d**) Liver KGDH latency. (**e**) Brain KGDH activity. (**f**) Brain KGDH latency. (**g**) Box plot of common eider egg yolk T in Iceland. Data from 2005 were adapted from Balk *et al*.^3^. Wilcoxon-Mann-Whitney test. (**h**) A 100 year survey of more than 20,000 common eider nests on 28 islands in the archipelago at F9 in the County of Stockholm. • Bars: arithmetic means. Whiskers: 95% CI (except in the box plot). C: control. Group comparisons are indicated by brackets.

For assessment of thiamine status in common eider females, we used domestic chicken as a surrogate control (Fig. 3a–f, Fig. S3a–e). A thiamine “complete” group and a thiamine “deficient” group were produced in the laboratory by giving domestic chickens different concentrations of thiamine in their fodder during 11–12 days. The mean dose (nmol T per g dry fodder) was 17.5 for the complete group and 1.2 for the deficient group. Figure 3a and Fig. S3a illustrates that the deficient group had substantially lower SumT and TMP concentrations in the liver than the complete group, and that these concentrations were very similar to those found in the common eider females from Iceland (D3) and Sweden (I10–14). Fig. 3b–f and Fig. S3b show the results of six other thiamine deficiency biomarkers in the same material: brain SumT, liver α-ketoglutarate dehydrogenase (KGDH) activity and latency, brain KGDH activity and latency, and proportion liver TDP. The general pattern was that thiamine status was substantially lower in the deficient group than in the complete group, and that the common eider females were more similar to the deficient group, except for brain SumT and brain KGDH latency in the Icelandic material. Moreover, when there was a difference between Icelandic and Swedish common eider females, the thiamine deficiency was more severe in Sweden. Thiamine deficiency is often associated with a biphasic relationship between proportion TDP and T concentration in various tissues (Fig. S3c–e). The mechanism behind this relationship is described in detail in the Supplementary Results. Accordingly, thiamine deficiency is indicated in the common eider females by the negative relationship between the proportion TDP and the T concentration in both the liver and brain (Fig. S3f,g). There was also a negative relationship between activity and latency for liver TK (Fig. S3h), liver KGDH (Fig. S3i), and brain KGDH (Fig. S3j).

Yolk T concentration was analysed in Icelandic common eider eggs sampled on several occasions between 2005 and 2013 (Fig. 3g). There was significant geographic and temporal variation in yolk T (Fig. 3g), indicating that the thiamine deficiency occurs episodically. The data in Fig. 3g do not, however, warrant conclusions about temporal trends. A majority of the eggs from D1 in 2008 and 2009 had a yolk T concentration under 5 nmol/g (Fig. 3g), which may be a threshold for the production of viable offspring. This threshold is based on the mean yolk T concentration 4.7 nmol/g, which was coincident with an almost complete absence of common eider pulli in the County of Södermanland (region G) during the breeding season 2005^3^. More recently, extremely low numbers of common eider pulli were observed also in the County of Blekinge (region I) coincidently with abnormal behaviour of both females and pulli (see Supplementary Results).

Figure 3h illustrates a 100 year survey of more than 20,000 common eider nests on 28 islands in an area (F9) within the Stockholm Archipelago. The dip between 1939 and 1961 was probably due to excessive hunting and environmental disturbances during the economic depression and the Second World War. Between 1964 and 2001, the population was relatively stable at around 1,100 nests per year. From 2001, there was a dramatic decline through 2016, when the total number of nests was only two, the lowest number ever. This development was anticipated in our work 2005–2009^3^. In recent years, large common eider population declines have been observed also in Iceland^19^. We suspect thiamine deficiency to be the cause of these recent population declines.

### Anguillid eels (*Anguilla spp.*)

**Figure 4:**
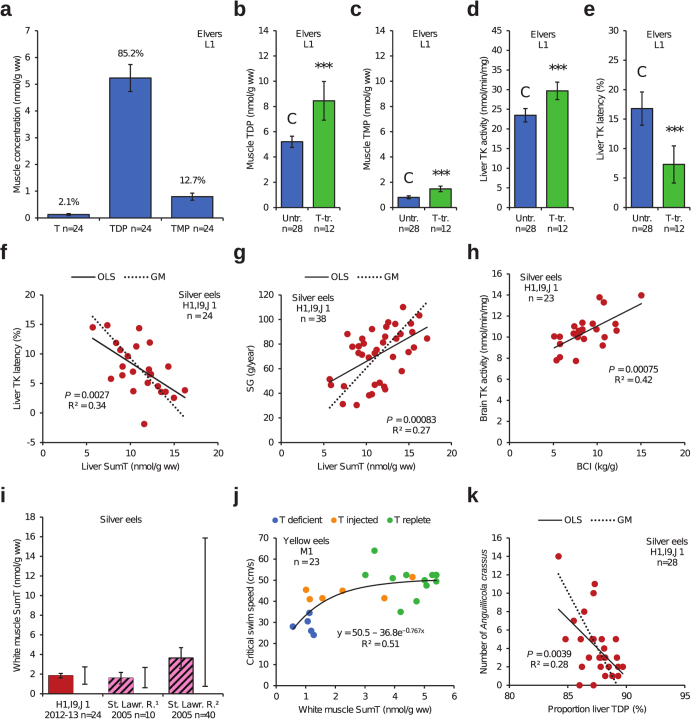
European eel (*Anguilla anguilla*) and American eel (*A. rostrata*). (**a*****–*****e**) Untreated (blue) and T-treated (green) elvers from L1 in the UK. The T-treated specimens were bathed twice in a 100 mg/L T solution for 48 h each time. (**a**) Concentration and proportion muscle T, TDP, and TMP. (**b*****–*****e**) Student’s *t*-test. (**b**) Muscle TDP. (**c**) Muscle TMP. (**d**) Liver TK activity. (**e**) Liver TK latency. (**f*****–*****h**) Female European silver eels in Sweden. (**f**) Liver TK latency and SumT. (**g**) SG and liver SumT. (**h**) Brain TK activity and BCI. (**i**) White muscle SumT in female European silver eels in Sweden (red) and two groups of American silver eels in St. Lawrence River, Canada (pink/black), adapted from Fitzsimons *et al*.^21^. The range is indicated to the right of each coloured bar. (**j**) Critical swim speed and white muscle SumT in experimental American yellow eels from M1 in Maryland, USA. (**k**) Number of the swim bladder parasite *Anguillicola crassus* and proportion liver TDP in female European silver eels in Sweden. • Bars: arithmetic means. Whiskers: 95% CI. OLS: ordinary least squares regression. GM: geometric mean regression.

Elvers were sampled at the mouth of River Severn (L1) in south-western England, UK, *i.e.* in the middle of the geographic range of the European eel (*Anguilla anguilla*). The proportions of T, TDP, and TMP in the muscle (Fig. 4a) were similar to those commonly reported in non-thiamine-deficient fish^20^, although the T proportion was somewhat lower. This finding suggests normal function of the thiamine phosphorylation within the cells. Thiamine deficiency was indicated, however, by the negative relationship between the proportion muscle TDP and the muscle T concentration (Fig. S4a). Thiamine deficiency was also indicated by the positive response to bathing in a T solution. Such treatment should have no effect on non-thiamine-deficient individuals, but muscle TDP, muscle TMP, and liver TK activity all increased (Fig. 4b–d), whereas liver TK latency decreased (Fig. 4e). Further indications of thiamine deficiency were illustrated by the negative relationships between liver TK activity and latency (Fig. S4b) and between liver TK latency and muscle TDP (Fig. S4c). The latter relationship indicated that the thiamine deficiency was of systemic nature. There was also a positive relationship between liver TK activity and muscle TDP (*P* = 0.028, R^2^ = 0.12, n = 40, not shown).

For silver eels there was a multitude of variables indicating severe thiamine deficiency both in the European eel and the American eel (*A. rostrata*). Liver TK latency was negatively related to liver SumT (Fig. 4f). Liver TK activity was positively related to liver SumT (Fig. S4d) and negatively related to liver TK latency (*P* = 0.042, R^2^ = 0.18, n = 24, not shown). There were also negative relationships between proportion TDP and T concentration in the white muscle (Fig. S4e), brain (Fig. S4f), and liver (Fig. S4g). The systemic nature of the thiamine deficiency was further indicated by the positive relationships between brain SumT and liver SumT (Fig. S4h), between somatic growth (SG) and liver SumT (Fig. 4g), between SG and liver/brain SumT ratio (Fig. S4i), and between brain TK activity and body condition index (BCI) (Fig. 4h). Mean white muscle SumT concentrations in the European and American silver eels were 1.6–3.6 nmol/g (Fig. 4i), although single specimens of the latter species had considerably higher white muscle SumT^21^. For example, the maximum white muscle SumT concentration observed in American silver eels 2005 was 15.9 nmol/g (Fig. 4i). It is thus likely that a white muscle SumT concentration of 1.6–3.6 nmol/g is far from sufficient for migration to the Sargasso Sea and production of viable (non-thiamine-deficient) eggs, as also suggested by Fitzsimons *et al*.^21^. The mass balance of SumT in the spawning female silver eel was modelled for two scenarios, which are available as Supplementary Information. The effect of thiamine deficiency on swimming endurance was analysed in American yellow eels (Fig. 4j). Swimming endurance was reduced at the white muscle SumT concentrations observed in many feral European and American eels today (Fig. 4i,j). Thiamine deficiency has been associated with immunosuppression in several investigations^*e.g.*^ 22, as well as with increased parasite infestation^23^. Hence, it is interesting to note the negative relationship between the number of the swim bladder parasite *Anguillicola crassus* and proportion liver TDP in silver eels (Fig. 4k), further analysed in the Supplementary Results. There was also a positive relationship between brain TK latency and the number of this parasite in the same specimens (*P* = 0.039, R^2^ = 0.20, n = 28, not shown).

### Salmonines (Salmoninae)

Here, we focus primarily on sublethal thiamine deficiency in both parents and their offspring of a number of salmonines. This approach differed from previous investigations within this scientific field, which focused mainly on mortality in the offspring. Our current focus provides a new perspective to the problem, and we have revisited existing literature to evaluate the prevalence of sublethal thiamine deficiency among salmonines in the Northern Hemisphere.

**Figure 5:**
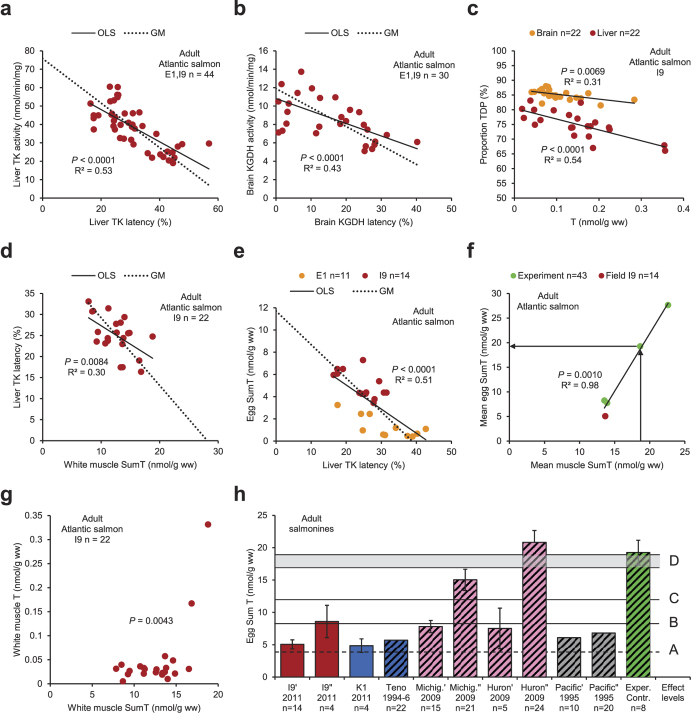
Salmonines (Salmoninae). (**a*****–*****g**) Atlantic salmon (*Salmo salar*). (**a**) Liver TK activity and latency in both sexes. (**b**) Brain KGDH activity and latency in both sexes. (**c**) Proportion TDP and T concentration in the brain and liver in both sexes. (**d**) Liver TK latency and white muscle SumT in both sexes. (**e**) Egg SumT and liver TK latency in females. (**f**) Mean egg SumT and mean muscle SumT in females from the field (red, this investigation) and experimental females (green, adapted from Fynn-Aikins *et al*.^20^). (**g**) The white muscle T concentration was low up to a white muscle SumT threshold of ca 17–19 nmol/g, where it started to increase. (**h**) Original data (plain bars) and literature data (striped bars, selection from Tables S5c,d) on egg SumT concentrations in mature female salmonines in the Northern Hemisphere. I9′, K1, River Teno, and experimental control: Atlantic salmon. I9″: sea trout (*S. trutta*). Lake Michigan’^,^”, Lake Huron’^,^”: lake trout (*Salvelinus namaycush*). Pacific coast’: Chinook salmon (*Oncorhynchus tshawytscha*). Pacific coast”: coho salmon (*O. kisutch*). (A) 3.9 nmol/g, threshold for mortality in the offspring; (B) 8.3 nmol/g, threshold for 20% reduced growth in the offspring^24^; (C) 12 nmol/g, threshold for liver TK latency in the parental females; and (D) 17–19 nmol/g, suggested threshold range for sublethal thiamine deficiency in the parental females and their offspring. • Bars: arithmetic means. Whiskers: 95% CI. OLS: ordinary least squares regression. GM: geometric mean regression.

In parental Atlantic salmon (*Salmo salar*) of both sexes from E1 and I9 in the Baltic Sea area, thiamine deficiency was indicated by the negative relationship between liver TK activity and latency (Fig. 5a), and even the liver TK latencies of 16–57% alone were evidence of severe sublethal thiamine deficiency. Liver TK activity and latency were also related to uncoordinated swimming (wiggling) in the parents (Fig. S5a,b). Brain KGDH activity and latency were negatively related and latencies of 0–40% were observed (Fig. 5b). The proportion TDP was negatively related to the T concentration, both in the liver and the brain (Fig. 5c). The thiamine status in the females was mostly lower at E1 than at I9, and lowest in the E1 females with mortality (lethal thiamine deficiency) in the offspring (Fig. S5c–h). The systemic nature of the thiamine deficiency was indicated by the negative relationships between liver TK latency and white muscle SumT (Fig. 5d) and egg SumT and liver TK latency (Fig. 5e), as well as the positive relationships between egg SumT and liver TK activity (Fig. S5j), white muscle SumT and total weight (Fig. S5k), liver SumT and heart SumT (Fig. S5l), liver SumT and brain SumT (Fig. S5m), and heart SumT and liver/brain SumT ratio (*P* = 0.0076, R^2^ = 0.31, n = 22, not shown).

Extrapolation to zero latency in Fig. 5d gave a white muscle SumT concentration of 28.2±6.8 nmol/g, and extrapolation to zero latency in Fig. 5e gave an egg SumT concentration of 11.7±2.3 nmol/g. These white muscle and egg SumT concentrations were not essentially different from those obtained by direct measurement in Atlantic salmon females, differing in thiamine status, by Fynn-Aikins *et al*.^20^. Their data demonstrated a strong positive relationship between mean egg and muscle SumT over a large range of SumT concentrations, and the means of our data from I9 fitted into this relationship (Fig. 5f), in which *e.g.* a muscle SumT concentration of 18.6 nmol/g corresponded to an egg SumT concentration of 19.3 nmol/g (arrows). An indication of how high the white muscle SumT concentration at least should be in a non-thiamine-deficient individual was provided by the threshold in Fig. 5g, where the white muscle T concentration was low up to a white muscle SumT concentration of ca 17–19 nmol/g, where it started to increase. The significance of this threshold was analysed with Fisher’s exact test (*P* = 0.0043), where the *P*-value represents the probability to obtain the two higher values at the end of the data array by pure chance. Egg SumT concentrations in a selection of mature female salmonines in the Northern Hemisphere are presented in Fig. 5h. (A more complete compilation of literature data on egg SumT concentrations of >4.0 nmol/g is given in the Table S5c,d). The indicated levels are as follows: (A) 3.9 nmol/g, threshold for mortality in the offspring (see Supplementary Results); (B) 8.3 nmol/g, threshold for 20% reduced growth^24^; (C) 12 nmol/g, threshold for liver TK latency (Fig. 5e); and (D) 17–19 nmol/g, suggested threshold range for sublethal thiamine deficiency (see Supplementary Results).

### Herring (*Clupea harengus*)

The herrings sampled here turned out to be essentially non-thiamine-deficient. Because they consisted of mature specimens that had migrated to their spawning grounds at the correct time of the year and were strong enough to catch the jig of spinning rods, they may constitute a non-random sample of the Baltic Sea herring population. The importance of these herrings was to show that non-thiamine-deficient individuals can still be found in the field, and that they exhibit biomarker responses that are not typical of thiamine deficiency (Fig. S6a–f, Table S6).

## Discussion

This investigation demonstrated severe thiamine deficiency in six out of seven studied aquatic species present in the Northern Hemisphere: blue mussel, common eider, European eel, American eel, Atlantic salmon, and sea trout. The analysed variables included activity and latency of a cytosolic (TK) and a mitochondrial (KGDH) thiamine-dependent enzyme in the digestive gland (blue mussels) and the liver and brain (vertebrates), as well as concentrations of T, TDP, and TMP in the digestive gland, liver, brain, muscle, heart, and eggs. Further analyses are presented in the Supplementary Results. The enzyme and thiamine variables indicated low thiamine status in a majority of the investigated specimens. We also analysed a number of secondary effect variables, such as—in the blue mussel: growth, condition, and reproduction—in the common eider: condition, liver size, histopathological alterations, and reproduction—in the European and American eel: swimming endurance, growth, condition, blood chemistry and composition, parasite infestation, and reproduction—and in the Atlantic salmon: swimming behaviour, growth, condition, blood chemistry and composition, and reproduction. There were several strong relationships between the thiamine deficiency variables and the secondary effect variables, indicating a wide range in thiamine status, typical for groups of specimens with various degree of thiamine deficiency, as described in the Methods. These relationships also indicated that many health effects, including abnormal behaviour, observed in biota today may well be due to thiamine deficiency. In many cases, we estimated the expected thiamine status of non-thiamine-deficient individuals, *e.g.* by extrapolation to zero latency, and generally, the hypothesized non-thiamine-deficient individuals had much higher thiamine status than the thiamine-deficient individuals. The episodic nature of the thiamine deficiency, in both time and space, was demonstrated by large differences in thiamine status between sampling groups. From a biochemical point of view, it is unlikely that the thiamine deficiency is caused by impaired phosphorylation within the cells, because the proportions of the phosphorylated forms were normal and the proportion TDP initially increased with increasing thiamine deficiency. Rather, the results point towards insufficient amount of thiamine in the food, as indicated by the relationship between the common eider and its main prey, blue mussels (see Supplementary Results). Such knowledge constitutes foundational information needed for the search for the causative agent(s). By investigating a large geographic area, by extending the focus from lethal to sublethal thiamine deficiency, and by linking biochemical alterations to secondary effects, we were able to demonstrate that the problem of thiamine deficiency is much more widespread and severe than previously reported.

The biogeochemical environment is highly impacted by human activities, both directly through the release of pollutants and nutrients, and indirectly through altered biogeochemical cycles of substances. The degree of human impact today, *e.g.* the number of man-made substances being released into the environment, is enormous compared with previous centuries. Consequently, the work to find a causative agent or agents for the thiamine deficiency will be a multi-step procedure. Before attempting to test possible candidates among a myriad of specific substances in exposure experiments, it is absolutely necessary to know which species, trophic levels, and geographic areas are affected by the thiamine deficiency, as well as its temporal variation. Such information should facilitate finding of the toxic mechanism, which in turn will be critical for finding the causative agent(s).

Decades of environmental research have proved that in order to avoid toxic effects in biota, including humans, man must stop releasing persistent pollutants into the environment. It is well known that lipophilic persistent pollutants biomagnify in the food web in a way such that top predators are most affected. Impaired health as a result of deficiency of an essential vitamin, however, surely differs from this paradigm, a presumption supported by the episodic nature of thiamine deficiency and its lack of correlation with exposure to classic lipophilic persistent pollutants. Although severe thiamine deficiency in salmonines has been observed since the early 1970s in both northern Europe and North America^1^, most of the research has focused mainly on mortality^25^,^26^ and not on sublethal effects. The present investigation sets out to demonstrate that this is inadequate, and it is tempting to draw a parallel to classic environmental pollutants, which also were investigated predominantly with respect to their lethal effects in the beginning (ca 1950–1970). It was not until the mid-1960s that concern started to arise about their sublethal effects, and doses orders of magnitude lower than those causing direct mortality were found to be important. Also for the classic environmental pollutants, the extension of the focus from lethal to sublethal effects revealed that the problem was much more widespread and severe than previously realized.

In order to understand the ecological implications of thiamine deficiency, it is important to remember that it always starts with molecular alterations on the subcellular level. Inactivation of thiamine-dependent enzymes is followed by an increase in toxic metabolites, such as lactate, glyoxals, and phytanic acid, as well as a decrease in life sustaining molecules, such as ATP, NADH, and NADPH. Such alterations compromise the integrity of tissues and organs, and their malfunction results in systemic disorders affecting central functions of life, such as growth, reproduction, immune defence, behaviour, nerve function, sensory functions, learning, and memory. Moreover, the metabolic disorders caused by thiamine deficiency are often only partially reversible, *i.e.* physiological conditions may not be fully restored, even though thiamine supply is fully restored. This has been demonstrated for both TK^27^,^28^,^29^,^30^ and KGDH^31^,^32^,^33^, as well as for other thiamine-dependent enzymes and metabolites^27^,^32^,^33^. One phenomenon that contributes to irreversible damage is focal cell necrosis, where dead cells are not replaced^34^,^35^,^36^,^37^. These relatively recent observations that a short-lasting (days–weeks) episode of thiamine deficiency may cause long-lasting (many years or for the rest of an organism’s life) sublethal effects^38^,^39^ add another dimension to the problem. In fact, many feral animals may have gone through one or more episodes of thiamine deficiency in recent years and thus suffer from a long-lasting or permanent reduction in thiamine-dependent enzyme activities. This phenomenon may have dramatic consequences for the majority of ecological research performed during the last decades, because it is not certain that animals display normal characteristics when subject to past and/or present thiamine deficiency. All kinds of characteristics may be affected, *e.g.* feeding, migration, behaviour, habitat preference, reproduction, and sex ratio. This is important to be aware of, especially since thiamine deficiency has been observed even in remote areas. For example, thiamine deficiency must be taken into account in the attempts to track animal movements, such as the silver eels’ migration to the Sargasso Sea, by use of telemetry equipment^40^,^41^,^42^. It is also possible that thiamine treatment of silver eels is necessary before viable offspring can be produced by hormonal treatment in the laboratory and in future aquaculture^43^,^44^,^45^,^46^. Another important phenomenon is that thiamine-deficiency-induced immunosuppression in feral animals involves increased spreading of pathogens between species. The avian influenza is a striking example, because feral water birds form the natural reservoir of these viruses^47^,^48^,^49^. During the last decade, several investigations of feral birds have pointed at the increased disease risk by immunosuppression, which is also reflected in altered disease dynamics^3^,^50^,^51^,^52^,^53^,^54^.

It is well known that populations lose much of their genetic variation when passing through a bottleneck. Recently, it has been demonstrated in seabirds that such loss of genetic variation also directly reduces the fitness of single individuals^55^. Despite the fact that biodiversity loss^56^,^57^ may well be the most important global threat to sustainability^58^, relatively few scientists have attempted to estimate the relative importance of the different factors contributing to this serious problem. Factors that have been proposed include habitat loss, global warming, and lack of food, although surprisingly often without explicit scientific references. Although the first two factors constitute serious problems, they are insufficient to explain the already ongoing global biodiversity loss^57^,^59^. For example, it is now 30 years since agricultural intensification ceased to explain the population declines of many farmland bird species^60^,^61^. It should also be remembered that global warming still mainly is a risk scenario of the future. The increase in global average surface temperature from 1951 to 2010 was within the range 0.6–0.7 °C^62^, which is notably less than feared future temperature increases. It may thus be time to revise several opinions about the dramatic current population declines^63^,^64^, sometimes referred to as the sixth mass extinction^65^. From 1970 to 2010/2012, population sizes of both terrestrial and marine vertebrate species dropped by half^66^,^67^, and from 1950 to 2010, the global seabird population declined overall by ca 70%^68^. We suggest that thiamine deficiency may be a significant contributor to this fatal process, and that the cause must be searched for at the chemical and biochemical levels.

## Methods

Thiamine concentrations were measured with High Performance Liquid Chromatography (HPLC) with fluorescence detection. Thiamine-dependent enzyme activities were measured spectrophotometrically in subcellular fractions. The detailed methods are described in the Supplementary Methods.

### How thiamine deficiency is demonstrated

Several facts support the concept that the latency of thiamine-dependent enzymes should be zero or very low in non-thiamine-deficient individuals. Literature data on TK and KGDH in organs, such as liver, kidney, and brain, in animals, such as mouse, rat, domestic chicken, European eel, and rainbow trout (*Oncorhynchus mykiss*), show that the mean latency of these enzymes in groups of control specimens is mostly 0–6%^3^,^28^,^69^,^70^,^71^,^72^,^73^,^74^,^75^,^76^,^77^, whereas higher latencies are often associated with formation of toxic metabolites^*e.g.*^ 78 and disease with more or less permanent damage^*e.g.*^ 38,39. For example, the decrease in TK activity during an episode of severe thiamine deficiency is only partly reversible^28^,^69^,^79^. Moreover, urinary excretion of thiamine strongly declines when latency starts to increase^80^. Obviously, the body strives to keep the latency low. From an evolutionary point of view, the ability to synthesize thiamine is believed to have been lost in animals during evolution, because it always has been abundant in the food^81^. Hence, throughout the biochemical literature, thiamine is customarily assumed to be present in sufficient amounts in non-thiamine-deficient individuals. Apparently, there is no such thing as “natural latency” and thus latencies higher than 6% are indicative of thiamine deficiency. Thiamine deficiency may, however, sometimes be present even in animals with low latencies, because the apoenzyme is not always maintained in thiamine-deficient tissues^73^,^74^,^75^,^82^.

Another important property of thiamine is that there is an upper limit for how much thiamine each organ in the body can hold, and any excess thiamine is excreted within a relatively short time^20^,^80^,^81^,^83^,^84^,^85^. Because thiamine normally should be present in sufficient amounts in the diet, there is no need for storing extra thiamine. To the best of our knowledge, there are no data in the biochemical literature indicating storage of excess thiamine in somatic tissues^81^. Together, these properties of thiamine suggest that the natural thiamine concentration in a non-thiamine-deficient tissue should not be substantially lower than the maximum observed thiamine concentration in this tissue. Therefore, thiamine concentrations substantially lower than the maximum concentration are indicative of thiamine deficiency.

Literature data show that thiamine concentrations and activities of thiamine-dependent enzymes may decrease to as little as 5–10% of control levels in single thiamine-deficient individuals^77^,^79^,^86^. Hence, the range of measured thiamine deficiency biomarker values are significantly larger in a group of specimens with different degrees of thiamine deficiency than in a corresponding control group, where all specimens are non-thiamine-deficient^*e.g.*^ 71,74,77,78,84,86,87. As a consequence, bivariate relationships between thiamine-deficiency biomarkers are often discernible only in groups of specimens with different degrees of thiamine deficiency, where the range of measured biomarker values is large, and not in non-thiamine-deficient groups of specimens, where this range is much smaller. This phenomenon has been observed in numerous investigations^*e.g.*^ 70,78,79,80,87,88,89,90,91. The observed relationships between thiamine deficiency biomarkers and secondary effects are also in agreement with the scientific literature on thiamine deficiency.

Relationships between latency and another biomarker allow extrapolation to zero latency, as an indication of what a control value of the other biomarker might be. The observed covariance between independently measured biomarkers also corroborate that our analyses were correctly performed. Finally, another way to demonstrate thiamine deficiency is remediation. Administration of thiamine to a non-thiamine-deficient individual should have no effect at all, whereas a therapeutic effect of thiamine proves that the specimen was thiamine-deficient prior to treatment.

### Animal care

All use of animals in this investigation was performed in accordance with the permits required by the respective national laws and local regulations. The sampling of common eiders (including eggs and pulli) in Sweden was approved by the Swedish Environmental Protection Agency, SEPA (Dnr. NV-02953–11) and the Stockholm Northern Research Ethics Committee (Dnr. N58/11). Permission to temporarily visit bird protection areas in the County of Blekinge 2010–2014 was granted by the County Administrative Board of Blekinge (Dnr. 521–441–10). The sampling of common eiders and their eggs in Iceland was approved by the Icelandic Ministry for the Environment and Natural Resources (UMH13060039, UMH05040093/12–1). The domestic chicken experiments were approved by the Stockholm Northern Research Ethics Committee (Dnr. N351/10, Dnr. N209/13). The purchase and direct import of elvers from the UK to Stockholm University was approved by the Swedish Board of Agriculture (Dnr. 6.2. 18–4928/13). The American eel swimming endurance experiment was approved by U.S. Geological Survey, Leetown Science Center Institutional Animal Care and Use Committee. The American eel maturation experiment was approved by the University of Massachusetts Dartmouth’s Institutional Animal Care and Use Committee (Protocol No. 09–03).

## Additional Information

**How to cite this article**: Balk, L. *et al*. Widespread episodic thiamine deficiency in Northern Hemisphere wildlife. *Sci. Rep.* **6**, 38821; doi: 10.1038/srep38821 (2016).

**Publisher's note:** Springer Nature remains neutral with regard to jurisdictional claims in published maps and institutional affiliations.

## Supplementary Material

Supplementary Information

## Figures and Tables

**Figure 1:**
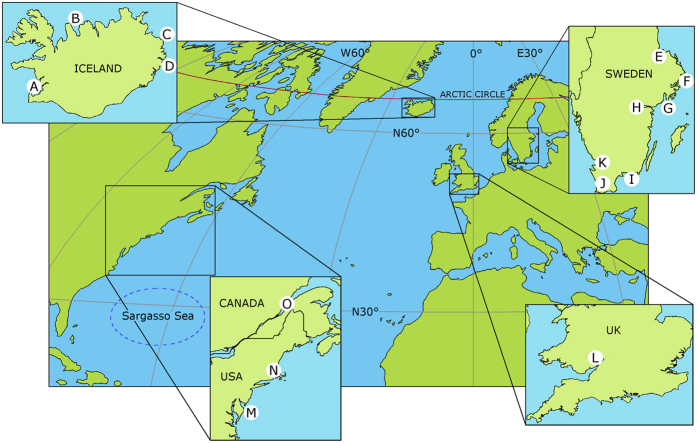
The 15 investigated regions in the Northern Hemisphere. (A) South-western Iceland. (B) Northern Iceland. (C) North-eastern Iceland. (D) Eastern Iceland. (E) County of Uppsala. (F) County of Stockholm. (G) County of Södermanland. (H) County of Östergötland. (I) County of Blekinge. (J) County of Skåne. (K) County of Halland. (L) Gloucestershire. (M) Maryland. (N) Massachusetts. (O) Quebec. • The European eel (*Anguilla anguilla*) and the American eel (*A. rostrata*) reproduce in the Sargasso Sea. The maps were modified with GIMP 2.8.16 (http://www.gimp.org/downloads/) from Free World Maps (http://www.freeworldmaps.net/printable/).
