# Supplementary material for: Widespread episodic thiamine deficiency in Northern Hemisphere wildlife
Source: Sci Rep. 2016 Dec 13;6:38821. doi: 10.1038/srep38821 (PMC5153840; doi:10.1038/srep38821)
Supplement: Supplementary Information [file srep38821-s1.pdf]

Supplementary Information for

Widespread episodic thiamine deficiency in Northern Hemisphere wildlife

Lennart Balk, Per-Åke Hägerroth, Hanna Gustavsson, Lisa Sigg, Gun Åkerman, Yolanda Ruiz Muñoz, Dale C. Honeyfield, Ulla Tjärnlund, Kenneth Oliveira, Karin Ström, Stephen D. McCormick, Simon Karlsson, Marika Ström, Mathijs van Manen, Anna-Lena Berg, Halldór P. Halldórsson, Jennie Strömquist, Tracy K. Collier, Hans Börjeson, Torsten Mörner & Tomas Hansson

**Contents**	**Pages**
Supplementary Methods	2–21
Supplementary Results	22–45
Blue mussel (*Mytilus sp*.)	22–24
Common eider (*Somateria mollissima*)	25–31
Anguillid eels (*Anguilla spp*.)	32–37
Salmonines (Salmoninae)	38–43
Herring (*Clupea harengus*)	44–45
Further Acknowledgements	46
Supplementary Figures S1–7	47–62
Supplementary Tables S1–6	63–75
Supplementary References	76–96

Supplementary Methods

Biological material. The seven feral species studied in this investigation were blue mussel (*Mytilus sp.*), common eider (*Somateria mollissima*), European eel (*Anguilla anguilla*), American eel (*A. rostrata*), Atlantic salmon (*Salmo salar*), sea trout (*S. trutta*), and herring (*Clupea harengus*). They represent the following three animal classes: bivalves (Bivalvia), ray-finned fishes (Actinopterygii), and birds (Aves). Moreover, farmed domestic chickens (*Gallus gallus*) with experimentally induced thiamine deficiency were used as a surrogate control for the field material. Samples were collected at 45 stations in Europe and North America (Fig. 1, Figs. S1a–o, Table S1). The stations were selected for their general prevalence of the investigated species, and not for reported occurrence of thiamine deficiency or other diseases. In Iceland, incipient thiamine deficiency in birds was demonstrated by us in a previous investigation[1]. Nevertheless, samples were collected in Iceland as representative of an area with less frequent occurrence of thiamine deficiency symptoms (*e.g.* paralysis) among birds[1]. The biological material was sampled rapidly and efficiently by a team of experienced personnel. When necessary, a mobile field laboratory was used to ensure the same rapid sampling of the material at all stations.

Chemicals. Bovine serum albumin (A4378), coenzyme A (C3144), 17α,20β-dihydroxy-4-pregnen-3-one (DHP; P6285), ethoxyresorufin (E3763), α-glycerophosphate dehydrogenase (G6751), HEPES (H4030), α-ketoglutaric acid (K1875), $MgCl_2$ (Ultra M2670), NaCl (S7653), NAD^+ (N1511), NADH (N8129), NADPH (N7505, ≥97%), resorufin (R3257), D-ribose 5-phosphate (R7750), sucrose (Ultra S7903), thiamine (T; T4625), thiamine diphosphate (TDP; C8754), thiamine monophosphate (TMP; T8637), triosephosphate isomerase (T2391), Tris-Cl (T3253), Triton X-100 (T9284), Trizma® base (T1503), and D-xylulose 5-phosphate (15807) were purchased from Sigma-Aldrich (St. Louis, MO, USA). DL-dithiothreitol (43819) was purchased from Fluka (Riedel-deHaën, Germany). Acetonitrile LiChrosolv® (1.14291.2500), copper sulphate *p.a.* (1.02791.0250), dipotassium hydrogen phosphate *p.a.* (1.05104.1000), disodium carbonate *p.a.* (1.06392.1000), Folin-Ciocalteu's phenol reagent (1.09001.0500), hydrochloric acid 30% Suprapur (1.00318.1000), potassium dihydrogen phosphate *p.a.* (1.04873.1000), potassium hexacyanoferrate (III) *p.a.* (1.04973.0250), potassium sodium tartrate *p.a.* (1.08087.500), and trichloroacetic acid (TCA) *p.a.* (1.00807.1000) were purchased from Merck (Darmstadt, Germany). Ethyl acetate, Baker-analyzed (9282-03), dipotassium hydrogen phosphate, anhydrous (0241), hexane, Baker-analyzed (8088), and sodium hydroxide, Baker-analyzed (0402) were purchased from J. T. Baker (Deventer, The Netherlands). Mayer's hematoxylin (01820) was purchased from Histolab Products AB (Göteborg, Sweden). Sodium hydroxide, EKA pellets (1.3303-1) was purchased from AkzoNobel (Bohus, Sweden). Salmon pituitary extract (SPE) was purchased from Argent Laboratories (Redmond, WA, USA). Heparin 100 IE/KY/mL and 5000 IE/KY/mL was purchased from LEO Pharma (Malmö, Sweden). Tricaine methanesulfonate (Tricaine-S, MS-222) was purchased from Western Chemical, Inc. (Ferndale, WA, USA). Eosin (B381402) and Luxol fast blue (B381750) were produced at the National Veterinary Institute (Uppsala, Sweden). Any other chemicals were purchased from common commercial sources and were of analytical purity. Pure water was produced by a Cyclon 4BD water still double distilled (Fistreem International Ltd., Loughborough, UK) or a Milli-Q Integral 3 system (Thermo Fisher Scientific Inc., Waltham, MA, USA).

Blue mussels. Systematically, blue mussels consist of at least three closely related taxa known as the *Mytilus edulis* complex[2]. The main component taxa are *M. edulis*, *M. trossulus*, and *M. galloprovincialis*. Collectively, they populate both coasts of the North Atlantic, including the Baltic Sea, as well as other parts of the world[3]. The component taxa have been shown to hybridize with each other, when present at the same locality[2,4,5]. For example, the Baltic Sea and the Swedish west coast are populated by both *M. edulis* and *M. trossulus*, as well as hybrids of the two[5]. Hybridization has recently been facilitated by human activities, such as shipping (ballast water), whereby taxa have been introduced to new areas. Since the taxonomic relationships within the *Mytilus edulis* complex are still under scientific debate, and we have not analysed our sampled blue mussels genetically, we refer to them simply as *Mytilus sp*. Blue mussels were collected manually with a hand net and/or with a 30 cm wide trawl handled by a rope from a small boat. In Iceland (regions A–D), blue mussels were collected at a depth of down to 1 m during the lowest tide each particular day (*i.e.* specimens periodically exposed to air were avoided). The only exception was 21 blue mussels collected at 7.0 m depth at A4 week 25 2013. In Sweden, blue mussels were collected in regions F, G, and I. All blue mussels from G1 were collected within a range of ca 40 m along the shore, and at a depth of ca 1.5 m. G1 is a narrow and shallow natural strait between two larger water basins. The current through the strait changes direction with irregular intervals of half an hour up to a few hours, owing to local differences in atmospheric pressure. (There is almost no tide in the Baltic Sea at this latitude.) The current seems to be beneficial for the blue mussels, since they were more abundant at G1 than at other places in region G, at least in 2011 when our sampling started. In regions F and I, blue mussels had to be collected within the depth range 1.0–8.5 m since they were not present at all depths. For a verification experiment, aquaculture blue mussels were also collected at the Swedish west coast. Within a few hours, the blue mussels were transported to the laboratory in a thermoinsulated plastic 30 L box filled with water from the collection site.

Blue mussel verification experiments. These experiments were performed to verify that the analysis of transketolase (TK) activity and latency as biomarkers of thiamine deficiency works also in blue mussels, since this has not been demonstrated previously. In order to artificially induce thiamine deficiency in the laboratory, the aquaculture blue mussels from the Swedish west coast were incubated in the air at room temperature for five days. Such treatment has been demonstrated to induce oxidative stress[6], which probably caused the observed thiamine deficiency in our aquaculture blue mussels. After this treatment they were returned to marine water from the collection site at field temperature for 16 h to partly recover before sampling. Their digestive gland was divided lengthwise in two equal halves (left and right). One half was used for TK analysis and the other half was used for thiamine quantitation. A corresponding experiment was performed with blue mussels from G1. These specimens were randomly assigned to the experimental groups. They were incubated in the air at room temperature for two days (medium stress) or four to five days (maximum stress), after which they were returned to brackish water from the collection site at field temperature for three days (medium stress group) or one day (maximum stress group) to partly recover before sampling. A control group of non-incubated G1 blue mussels was also sampled. In this experiment the whole digestive gland was used for TK analysis.

Sampling of blue mussels. On each sampling occasion, ca 24 specimens were sampled within a few hours after arrival at the laboratory. This group, called “common eider food”, was later analysed for thiamine in order to determine how much thiamine the common eider actually obtains

from its major prey item. Another group of ca 18 specimens were left overnight for defecation at the same temperature as where they were collected. Initially, a control experiment for this treatment was performed, where the digestive gland was analysed for activity and latency of TK, and whereby no effect of the overnight storage and defecation was observed. At sampling, specimens were lightly blotted on a paper tissue and weighed (total weight including shell and inside water) to the nearest 0.01 g. Length, width, and height of each specimen were then measured with an electronic digital calliper to the nearest 0.1 mm. In order to investigate size differences, all blue mussels, except those from the Swedish west coast, were classified by length as either small (15<length≤23 mm), medium (23<length≤27 mm), or large (27<length≤38 mm). In the collected material from the Baltic Sea and Iceland, 80% (2,100 of 2,640) of the specimens were 19–31 mm long. The "common eider food" group usually contained 6 small, 12 medium, and 6 large specimens, whereas the "defecation" group usually contained 6 specimens of each length class. Accordingly, there was an even distribution of lengths within the interval 19–31 mm. In the "common eider food" group, each specimen was opened by cutting off the adductor muscles with a lancet, and the soft body was dissected, lightly blotted on a paper tissue, weighed to the nearest 0.01 g, put in a cryotube, submerged in liquid nitrogen, and stored at –140 °C until thiamine quantitation. In the "defecation" group, each specimen was opened by cutting off the adductor muscles with a lancet, and the digestive gland was dissected with forceps and a pair of ophthalmic scissors (Fine Science Tools GmbH, Heidelberg, Germany) under a Leica MZ8 stereomicroscope (Leica Microsystems GmbH, Wetzlar, Germany), lightly blotted on a paper tissue, weighed to the nearest 0.01 g, and homogenized in 400 µL ice-cold 0.25 M sucrose in a 500 µL Potter-Elvehjem homogenizer (size 18) with four up and down strokes at 400 rpm and under constant cooling with ice-water. The homogenate was diluted with another 400 µL ice-cold 0.25 M sucrose, transferred to a 2 mL Eppendorf tube, and centrifuged at 10,000 g_{av} and 4 °C for 10 min in an Eppendorf 5415R centrifuge (Eppendorf, Hamburg, Germany). Aliquots of the supernatant were put in cryotubes, submerged in liquid nitrogen, and stored at –140 °C until analysis of TK activity and latency. Storage at this ultra-low temperature is an important measure of precaution, especially for preservation of the apoenzymes, which may be less thermostable than the holoenzymes. The remaining soft body of each specimen was dissected and processed in the same way as the soft bodies of the "common eider food" group. The sampling of each specimen was performed without interruption, although two specimens were sometimes sampled in parallel. In order to facilitate comparison with blue mussels described in the literature, we also sampled additional blue mussels from G1 for soft body dry weight determination. During 2011–2014, on eight occasions randomly distributed over the year, 30 additional blue mussels were sampled for this purpose (in total 240 specimens). They were dissected in the same way as the specimens of the "common eider food" group.

Dry weight determination and calculation in blue mussels. Most previously published measurements of blue mussel condition involve soft body dry weight determination. Both thiamine concentration and TK activity and latency are thermolabile, however, and cannot be measured in dried material. Therefore, we determined the mean soft body dry weight proportion in the 240 additional blue mussels from G1 sampled for this purpose. The specimens were dried at 95 °C for 24 h, allowed to cool in a desiccator, and weighed to the nearest 0.01 g. The mean soft body dry weight proportion had a range of 13.4–16.8%, and there was no pattern in the variation over the year. Hence, we used a grand mean of 14.9% for calculation of the dry weight of all Baltic Sea blue mussels that were not dried, irrespective of the time of the year. In the investigations by

Kautsky[7] and Öst & Kilpi[8], the drying was performed at 60 °C for 48 h. Hence, we compared the two drying regimes in a control experiment, where the blue mussels (n=30) were first dried at 60 °C for 48 h and weighed, and then dried at 95 °C for additional 24 h and weighed again. The 95 °C drying regime yielded a 4.4% lower dry weight than the 60 °C drying regime (67.9±11.7 mg instead of 71.3±12.6 mg). This difference is too small to have any significant influence on the comparison of our results with those of Kautsky[7] and Öst & Kilpi[8]. In order to match the length groups 20 mm and 30 mm defined by Kautsky[7], two length groups denoted 21 mm and 29 mm were composed of blue mussels from G1. They consisted of specimens in the length intervals 17–23 mm and 27–33 mm, respectively.

Body condition index (BCI) for blue mussels. To allow analysis of thiamine and TK in the blue mussels (no drying) we defined BCI as the wet soft body weight (g) divided by the total specimen volume (cm^3). The total specimen volume was calculated as the product of specimen length, width, and height, and an empirically determined constant, which took the values 0.45 in the Baltic Sea, 0.46 in Iceland, and 0.39 at the Swedish west coast (45–90 mm long blue mussels). The constant was calculated from the total weight of specimens completely filled with water at the beginning of the sampling, taking into account wet shell weight and density, water density, and wet soft body density. The mean wet shell density was calculated from the wet shell weight and volume. The wet shell volume was determined by immersion of several shell halves in water in a graduated cylinder. The mean wet shell density was 1.14 g/cm^3 in the Baltic Sea, 2.40 g/cm^3 in Iceland, and 2.47 g/cm^3 at the Swedish west coast. The wet soft body density was assumed to be 1 g/cm^3.

Chlorophyll *a* in region G. Data on chlorophyll *a* concentration in region G were obtained from the database Svenskt HavsARKiv (SHARK) provided by the Swedish Meteorological and Hydrological Institute (SMHI). The data were produced within Swedish coordinated environmental monitoring by the Swedish Environmental Protection Agency (SEPA). The chlorophyll *a* concentration at 2.5 m depth was calculated as the arithmetic mean of the values at 0 and 5 m depth. For chlorophyll *a* determination, water was collected with a 5 L Niskin water sampler. Within 16 h, a volume of 1–2 L was filtered on a 47 mm Whatman GF/F filter in reduced light. After filtration, 3 mL of 1% magnesium hydroxide carbonate suspension per litre sample was added to the filter, which was stored at −20 °C until analysis (no longer than 3 months). Chlorophyll *a* was extracted with ethanol at room temperature in darkness for 6–24 h. During this time the sample was thoroughly shaken at least three times, and once more before centrifugation. The sample was centrifuged twice at 2,950 rpm for 5 min. Chlorophyll *a* absorbance was measured with a Hitachi U-2000 Spectrophotometer (Hitachi, Tokyo, Japan) at 664 nm and corrected for background at 750 nm. Chlorophyll *a* concentration was calculated according to the formulae defined by Jeffrey and Humphrey[9]. Samples were measured in duplicate, normally from the same Niskin water sampler after thorough mixing of the sample. A certified reference material, consisting of a known amount of chlorophyll *a* dissolved in acetone, was analysed at least twice a year. Moreover, a laboratory reference material (LRM) was analysed in parallel with the samples on every occasion. The LRM consisted of chlorophyll extracted with ethanol or acetone, and was stored at −20 °C.

Common eiders. On the European mainland, the common eider is a short-distance migrant, whereas in Iceland, it is a non-migrant. Several investigations have shown that blue mussels are an important part of the common eider diet[*e.g.* 8,10–15]. The preferred length of the blue mussels in

these investigations varied somewhat between places: 8–28 mm (mean ca 17 mm) in the northern Baltic Proper[8]; 30–40 mm in Kattegat[10]; 16–41 mm (mean ca 27 mm) in middle Scotland in November–February[12]; mean 23 mm in north-eastern Scotland[14]; and 5–29 mm (mean 14 mm) in northern Norway[16]. Accordingly, the blue mussels sampled by us, within the main length interval 19–31 mm, were well representative of the common eider food.

Sampling of adult common eider females. A total of 23 adult common eider females were sampled in region I during the breeding season in 2011. A corresponding material of 12 specimens was sampled in regions C and D in 2013. The females were caught with a large hand net while incubating their offspring in the nest. The female and its entire nest (including eggs and/or pulli) were placed in a pet transport box and brought to the laboratory and sampled within a few hours after collection. It is our impression that this procedure, not to separate the female from its nest and offspring, kept the female calmer than it would have been otherwise. While waiting to be sampled, one of the females from region I died from a massive heart attack, as determined by post-mortem examination. This is especially interesting, because heart failure may be caused by thiamine deficiency[17]. (Since this specimen was found dead some time after the heart attack, it was excluded from the sampling.) Each specimen was decapitated and weighed to the nearest 1 g. The obtained weights were later adjusted for loss of blood at decapitation by multiplication with a factor of 1.03[18]. Liver and brain were dissected and weighed to the nearest 0.01 g. For thiamine quantitation, a liver piece and the right half of the brain were put in cryotubes, submerged in liquid nitrogen, and stored at –140 °C until analysis. For analysis of thiamine-dependent enzymes, a central piece of the liver was cut into smaller pieces with a pair of scissors and homogenized in an equal volume of ice-cold 0.25 M sucrose in a 10 mL Potter-Elvehjem homogenizer (size 21) with five up and down strokes at 400 rpm and under constant cooling with ice-water. The homogenate was diluted to 20% with ice-cold 0.25 M sucrose, transferred to 2 mL Eppendorf tubes, and centrifuged at 10,000 g_{av} and 4 ºC for 10 min in an Eppendorf 5415R centrifuge (Eppendorf, Hamburg, Germany). The supernatants were mixed and aliquots were put in cryotubes, submerged in liquid nitrogen, and stored at –140 °C until analysis of TK activity and latency. The remaining pellets in the 2 mL Eppendorf tubes were submerged in liquid nitrogen, and stored at –140 °C until analysis of α-ketoglutarate dehydrogenase (KGDH) activity and latency. The left half of the brain was also allocated to analysis of thiamine-dependent enzymes. Brain 10,000 g_{av} supernatants and pellets were prepared in the same way as for the liver pieces. Samples for histopathological examination were dissected from the liver, myocardium, kidney, pancreas, intestine, lung, ovary, and thigh muscle and fixed in 10% neutral buffered formalin. No histopathological samples of the brain were taken, because the whole brain had to be allocated to analysis of thiamine and thiamine-dependent enzymes. This procedure was necessary in order to sample the brain in exactly the same way as for other bird specimens. Peripheral nerves and ganglia were, however, present in the other tissue samples and were thus included in the histopathological analysis. The sampling of each specimen was performed without interruption and completed before the next specimen was decapitated and sampled. The nests were classified by the amount of down in the categories “normal” (50–100%), “less than normal” (5–50%), and “much less than normal” (0–5%), where 100% represents a nest fully furnished with down. Some eggs were addled and/or contained a dead embryo or foetus. The percentage of such eggs in each clutch was recorded.

Sampling of common eider eggs. 207 common eider eggs were collected in Iceland (regions A–D) in 2005–2013. Only newly laid eggs were used, and the criterion for this was absence of blood

vessels. The eggs were weighed to the nearest 0.01 g, and length and width were measured with a Vernier calliper to the nearest 0.1 mm. In domestic chicken eggs, the yolk T concentration is more than ten times the white T concentration on a wet weight basis[19,20], and in the yolk, both plasma and granules contain significant amounts of T[21]. Similar conditions are assumed in common eider eggs. Therefore, egg yolk was sampled with a 2 mL syringe (without needle) to avoid contamination with white, and put into cryotubes, which were submerged in liquid nitrogen, and stored in liquid nitrogen (samples from 2008–2009) or at –140 °C (remaining samples) until thiamine quantitation. Egg weight loss due to water evaporation between laying and sampling was obtained as the difference between the initial weight and the measured weight. The initial weight was calculated from the fresh egg density (determined by us) and the volume, which was calculated[22] from length, width, and a shape constant[23]. The yolk T concentration was adjusted for the water loss, using a literature value[24] of the yolk water content, whereby the relative decrease in yolk water content was approximated by the relative egg water loss. The egg water loss was generally 0–10% of the initial egg weight, resulting in a 0–5% adjustment of the yolk T concentration, since ca half of the yolk was water.

Breeding common eider survey in region F. The Lygne Archipelago is situated on the outskirts of the Stockholm Archipelago. The total area is 1.3 km², of which 0.2 km² is land, distributed on a number of relatively small islands of different sizes. The vegetation, which is concentrated to the larger islands, is characterized by low trees, such as birch, rowan, and alder, and ground cover, such as creeping juniper, heather, crowberry, and swamp fragments. The shores are mainly composed of flat rocks, boulders, and occasionally a small meadow. The Lygne Archipelago has probably been a place for fishing and hunting since the Middle Ages or even longer. There are traces of settlements of seasonal inhabitants. Since the mid-1800s, the bird populations have been regularly monitored, although reliable quantitative data on the number of common eider nests are available only from 1910 and onwards. This survey, which is without parallel within Sweden, was performed with relatively even intervals (mostly three years), and care was taken to include only nests built the current year, as judged by *i.a.* the presence of egg shells and/or down. In 1953, the Lygne Archipelago was established as a game and bird preservation area by the Stockholm County Administrative Board. In 1983, the bird preservation area was restricted by the same authority to 12 islands within the Lygne Archipelago with prohibition against going closer to an island than 100 m off the shore during April 1 to July 31. Mink traps and hunting with dogs have kept the area mostly free of mink. Despite high natural and recreational values, the Lygne Archipelago is sparsely visited by tourists, perhaps a total of ca 30 short-staying boats during a sunny summer. Accordingly, the human pressure on the area is comparatively low. The breeding common eider survey curve presented here includes only those 28 stations that were monitored throughout the entire period from 1910 until 2016. The counting of nests must be regarded as a very reliable method in comparison with many other methods for monitoring of bird populations.

Domestic chicken experiments. Three domestic chicken experiments with various doses of T in the food were performed as a surrogate control for effects of the thiamine deficiency observed in biological material from the field. Despite species differences, there are many similarities in the physiological response to thiamine deficiency, making the inter-species comparison valid. Newly hatched domestic chickens (SweHatch, Väderstad, Sweden), weighing ca 40 g, were given a thiamine-free domestic chicken fodder (Special Diets Services, Essex, UK), supplemented (by us) with T at various doses. The thiamine-free fodder was claimed to contain <0.4 nmol thiamine per

g by the manufacturer, and our own analysis confirmed that it contained <0.1 nmol T per g, <0.1 nmol TMP per g, and <0.3 nmol TDP per g. For all other nutrients, the fodder fulfilled the dietary requirements of domestic chicken as specified in "Nutrient Requirements of Poultry, Ninth Revised Edition, 1994"[25]. The water content of the dry fodder was 8.3%. The experimental groups were matched with respect to start weight and otherwise randomized. All experimental groups were pair-fed with respect to the lowest dose group to ensure that all groups got the same amount of food. The experiments were terminated when the lowest dose groups started to show clinical symptoms of thiamine deficiency. In the first experiment, the aim was to produce reference values for the investigated thiamine deficiency biomarkers for comparison with the adult female common eiders. The experiment included a "deficient" group, given a T dose (nmol/g dry fodder) of either 0.9 (n=7) or 1.5 (n=8), and a "complete" group, given a T dose of either 10 (n=7) or 25 (n=7). The rationale of having a range of T doses in each group was to achieve a more relevant comparison with the adult female common eiders. Moreover, the dose range 0.9–1.5 nmol/g was chosen to obtain evident symptoms of thiamine deficiency on the biochemical level. In the second experiment, the aim was to produce a dose-response curve for the investigated thiamine deficiency biomarkers. The included T doses (nmol/g dry fodder) were 0.3 (n=8), 0.9 (n=15), 1.5 (n=8), 2.5 (n=8), 5 (n=7), and 10 (n=7). For histopathological analysis a third experiment was performed, where domestic chickens (n=9) were given the thiamine-free fodder without any supplement of T. In experiment one and two, sampling was performed on day 11 and 12, when the domestic chickens had grown by 0–50 g, whereas in the third experiment, sampling was performed on day 10. All domestic chickens were sampled in the same way as the adult female common eiders, except that the samples for histopathological examination were dissected from the brain, liver, myocardium, kidney, and calf muscle.

Body indices for birds. A body condition index (BCI) was defined as the body weight (hg) divided by the brain weight (g). A liver body index (LBI) was defined as the liver weight expressed as percent of the body weight.

European and American eel. The European and American eels have five distinct life stages: leptocephalus, glass eel, elver, yellow eel, and silver eel. Spawning has been suggested to occur in the Sargasso Sea[26–28] and the leptocephali partly swim and partly drift with the currents to the European and American coasts. The time of this journey has been estimated to at least 8–9 months and up to three years for the European eel[29–31]. The extent and nature of the feeding during this time is largely unknown, although gelatinous marine plankton, so-called marine snow, have been suggested to be included in the diet[32]. Upon arrival at the coast, the leptocephali metamorphose into semi-transparent glass eels, which may remain in the estuary for up to a year[33]. During this time, the glass eels start to gradually transform into elvers, which migrate further upstream[33] into or close to freshwater habitats[34]. The elver stage is followed by the yellow eel stage, which may last for 3–30 years[29,35,36]. This stage is a period of feeding and growth[34], which ends with transformation of the yellow eels into silver eels, when they are ready to begin their migration back to the Sargasso Sea for spawning[37,38]. For the European eel, this journey of 5,000–6,000 km[39] has been estimated to take four to eight months with a peak at six months[40]. For the American eel, the migration journey is 1,000–5,000 km[41] and may take two to six months, based on migration start in August–December[41] and peak spawning in February[30]. The transformation of yellow eels into silver eels is associated with extensive physiological alterations. Besides the change in pigmentation, there is an increased synthesis of growth hormone (GTH-II) by the pituitary[42,43], and

the gonad starts to grow. Female European silver eels were caught at H1, I9, and J1 in Sweden in the early autumn 2012 and 2013. Their gonadosomatic index (GSI, defined below) was 1.1–1.9%. Such high values occur almost exclusively (to ca 99%) in silver eels[43,44]. This criterion together with the morphological characteristics length, weight, eye diameter[45], and pigmentation assured that the collected female European eels were, with high probability, correctly classified as silver eels. European elvers were caught at L1 in the UK in late April 2013. This area and its prevalence of European eel have been described in detail by Aprahamian[46,47]. The juvenile eels were unmistakably pigmented and thus considered to have reached the elver stage[48,49]. Female American silver eels were caught at N1 (MA, USA) and O1 (QC, Canada) in the autumn 2011, whereas American yellow eels were caught as glass eels at M1 (MD, USA) in 2010. Both in Sweden and USA, silver eels were caught with different types of eel traps at the various locations. The traps were also located in different environments (*e.g.* in a river or in a lake). As a result, the traps differed in fishing efficiency (*e.g.* with respect to the swimming activity of the eels). This may have caused a bias in the thiamine status of the eels, and as a precaution, no comparisons of thiamine status were made between regions within Sweden. Both the European elvers and the American glass eels were caught with a hand net. Because of the obvious difficulty to catch spawning female silver eels with eggs in the Sargasso Sea, egg production was induced by gonadotropin treatment of female American silver eels in the laboratory to obtain information about the transfer of thiamine to the eggs. The European elvers were used in an experiment, where the effect of thiamine treatment on thiamine deficiency biomarkers was investigated. The American yellow eels were used in an experiment, where the effect of thiamine status on swimming endurance was investigated.

Eel swim bladder parasite. The investigation of female European silver eels included counting of the swim bladder parasite *Anguillicola crassus*, which is a nematode. This parasite has been claimed to originate from South-east Asia[50] and it was not observed in Western Europe until the early 1980s[51]. It was reported for the first time in the UK in 1987[52], and in Sweden it was described as a new parasite in 1988[53]. From the mid-1990s, it has been found also in American eels in North America[54]. The life cycle of *A. crassus* includes small copepods as intermediate hosts, other fish as facultative reservoir hosts, and anguillid eels as final hosts[55]. An outlier with 37 *A. crassus* was omitted, since the number of this parasite in all the other European silver eels was in the range 0–14.

Female European silver eel sampling. Each specimen of the silver eels was placed upside down in an oblong, V-shaped plywood box and held by two persons. In this position, the total length was measured to the nearest 1 cm, and a blood sample was drawn from the caudal vein with a heparinized (100 IE/KY/mL) 2 mL syringe, gently turned several times to prevent the blood from clotting. The specimen was decapitated and weighed (including head) to the nearest 1 g. While the body was still held by two persons, liver, heart, gonad, and brain were dissected and weighed to the nearest 0.01 g. The brain was so small (0.07–0.17 g) that it was allocated entirely to either thiamine quantitation or analysis of TK and KGDH. The otoliths were dissected for age determination. For thiamine quantitation, pieces of the liver and epaxial white muscle, as well as the entire heart, and, for ca half of the specimens, the entire brain were put in cryotubes, submerged in liquid nitrogen, and stored at –140 °C until analysis. The number of *A. crassus* was recorded. For analysis of thiamine-dependent enzymes, a central piece of the liver was cut into smaller pieces with a pair of scissors and homogenized in an equal volume of ice-cold 0.25 M sucrose in a 10 mL

Potter-Elvehjem homogenizer (size 21) with five up and down strokes at 400 rpm and under constant cooling with ice-water. The homogenate was diluted to 20% with ice-cold 0.25 M sucrose, transferred to 2 mL Eppendorf tubes, and centrifuged at 10,000 g_{av} and 4 ºC for 10 min in an Eppendorf 5415R centrifuge (Eppendorf, Hamburg, Germany). The supernatants were mixed and aliquots were put in cryotubes, submerged in liquid nitrogen, and stored at –140 °C until analysis of TK activity and latency. The remaining pellets in the 2 mL Eppendorf tubes were submerged in liquid nitrogen, and stored at –140 °C until analysis of KGDH activity and latency. For ca half of the specimens, 10,000 g_{av} supernatants and pellets of the entire brain were prepared in the same way as for the liver pieces. The precaution to store the samples for enzymatic analysis at –140 °C was specifically motivated by the fact that the thermostability of the apoenzyme has been demonstrated to be lower than that of the holoenzyme for TK in the European eel[56]. Blood glucose, haemoglobin, and haematocrit were measured in duplicates directly at the sampling. Blood glucose was measured with the HemoCue® Glucose 201 RT System (HemoCue AB, Ängelholm, Sweden), based on the methods presented by Banauch *et al*.[57] and Bergmeyer[58]. Haemoglobin was measured with the HemoCue® Hb 201$^+$ System (HemoCue AB, Ängelholm, Sweden), based on the method presented by Vanzetti[59]. Haematocrit was determined according to the standard procedure for the haematocrit centrifuge Haemofuge no. 780 (Heraeus Christ, GmbH, Osterode, Germany). A glass capillary, filled with ca 24 µL blood, was centrifuged for 2 min and read with a haematocrit reader. The sampling of each specimen was performed without interruption and completed before the next specimen was sampled. Age was determined from the otoliths according to the "ICES WKAREA report 2009"[60]. It is customary to refer to the age of eels in fresh water in terms of years post metamorphosis[49]. Thus an elver is age zero.

European elver experiment and sampling. The elvers were a sample from a large batch imported to Sweden by Scandinavian Silver Eel AB (Helsingborg, Sweden). They were 6–8 cm long and weighed 0.17–0.40 g when they arrived at our laboratory on day one. They were all in seemingly good condition, swimming vigorously, and there was no mortality. Retrospectively, we were informed that the rest of the batch imported to Sweden did not develop any diseases or mortality in the mandatory quarantine, from which the experimental sample was exempt. The elvers were offered Atlantic cod (*Gadus morhua*) roe during the course of the experiment, but did not eat. The elvers were randomly assigned to the experimental groups. A total of 24 untreated specimens were sampled on day two, and four additional untreated specimens were sampled on day ten. A total of 12 specimens were bathed twice (day five and eight) in 2 L of a 100 mg/L T solution for 48 h each time. The thiamine treated specimens were sampled on day ten. There was no mortality during the experiment. Each specimen was weighed to the nearest 0.01 g and decapitated. For analysis of TK, the liver was dissected in a Petri dish on ice under a Leica MZ8 stereomicroscope (Leica Microsystems GmbH, Wetzlar, Germany). The liver was homogenized in 300 µL ice-cold 0.25 M sucrose in a 500 µL Potter-Elvehjem homogenizer (size 18) with five up and down strokes at 400 rpm and under constant cooling with ice-water. The homogenate was diluted with another 400 µL ice-cold 0.25 M sucrose, transferred to a 2 mL Eppendorf tube, and centrifuged at 10,000 g_{av} and 4 °C for 10 min in an Eppendorf 5415R centrifuge (Eppendorf, Hamburg, Germany). Aliquots of the supernatant were put in cryotubes, submerged in liquid nitrogen, and stored at –140 °C until analysis of TK activity and latency. For thiamine quantitation, the decapitated body rest was weighed to the nearest 0.01 g, put in cryotubes, submerged in liquid nitrogen, and stored at –140 °C until analysis. The sampling of each specimen was performed without interruption and completed before the next specimen was sampled.

American yellow eel swimming endurance experiment and sampling. Initially, a total of 30 specimens were randomly divided into two groups, "replete" and "deficient". The groups were placed in two cylindrical flow-through basins, which were 60 cm in diameter and had a depth of 28 cm. Water temperature was maintained at 19 °C and flow was maintained at 6.5 L/min during the course of the experiment. The "replete" group was fed a basal fish meal diet supplemented with ca 38 nmol T per g, whereas the "deficient" group was initially fed the same basal diet supplemented with bacterial thiaminase and no T. The latter diet has been used previously to induce thiamine deficiency in lake trout (*Salvelinus namaycush*)[61]. The "deficient" group was maintained on this diet until the specimens started to show signs of thiamine deficiency, such as uncoordinated swimming and mortality, paralleled by low muscle thiamine concentrations. A few specimens, both with and without uncoordinated swimming, were sampled for confirmation of the low muscle thiamine concentrations. These specimens were killed with an overdose of MS-222 followed by cervical dislocation. Muscle tissue was immediately dissected and stored at –80 °C until thiamine quantitation. At this point, the diet was changed to basal diet supplemented with ca 1.5 nmol T per g and without bacterial thiaminase. This dietary T concentration prevents mortality but maintains a low thiamine status in the specimens[62]. The yellow eels were maintained under these conditions for over a year and they weighed at least 20 g at the time of the swimming endurance experiment. Owing to sampling and mortality, there were 12 specimens left in the "replete" group and 11 specimens left in the "deficient" group. In order to further characterize the effect of thiamine on swimming endurance, an intermediate group was created just prior to the experiment. Six randomly selected specimens from the "deficient" group were injected with thiamine 2–4 h prior to the experiment. The injection contained 15 μmol T dissolved in sterile saline, pH=6.8, per specimen. The yellow eels were individually placed in the swimming chamber, which was 30.5 cm in diameter and 70 cm long. A complete description of the swimming chamber is given by Leonard *et al.*[63]. The water temperature was maintained at 18–20 °C. Each specimen was acclimatized to the chamber for 60 min with the water flow set at 10 cm/s. The water flow was then incrementally increased by 10 cm/s every 20 min and the time until exhaustion was observed. The "critical swim speed" was interpolated according to the principle described by Brett[64]. Once exhausted, the yellow eels were removed from the swimming chamber and killed with an overdose of MS-222 followed by cervical dislocation. Each specimen was weighed to the nearest 0.1 g, and the total length was measured to the nearest 1 mm. Liver and white muscle samples were immediately dissected, placed in sample containers, and stored at –80 °C until thiamine quantitation. In the evaluation of the effect of white muscle SumT on critical swim speed, both total length and total weight were found insignificant as confounding variables, *i.e.* the material was homogeneous with respect to these size variables.

American silver eel maturation experiment and sampling. The maturation experiment was performed according to the protocol presented by Oliveira & Hable[65]. This protocol has been successful for the production of fertilizable American eel eggs. A total of 13 field-collected silver eels, ranging from 405 to 2,169 g, were maintained in 1,000 L basins connected to a seawater (29–31 ‰) flow-through system at 20 °C during the experiment. The basins were exposed to ambient daylight from windows and thus received ca 10 h light and 14 h darkness per day. The silver eels were allowed to acclimatize to the laboratory conditions for two weeks prior to the beginning of hormonal treatment. Since eels are presumed not to eat during migration[48], they were not fed during the acclimation or maturation. Each week, the females were lightly sedated, weighed to the

nearest 1 g, and given injections of 10 mg SPE in 0.5 mL saline per specimen. When females showed a continuous body mass increase of >5% per week, oocytes were sampled weekly by aspiration biopsy using a 16-gauge needle inserted into the body cavity approximately 5–8 cm anterior of the urogenital opening[66]. Oocytes were considered approaching maturation when microscopic examination showed that they were nearing 800 µm in diameter and fulfilled several morphological criteria described by Oliveira & Hable[65]. At this time, a booster injection of 10 mg SPE was given, and the following day, ovulation was induced with an injection of 2 mg DHP per g body mass, 1.67 mg/mL in a saline:ethanol 1:1 (v:v) mixture. DHP injections were given as a series of three injections per side of the eel 3–4 cm from the ventral midline and presumably directly into the ovaries. Upon ovulation, eggs were collected by hand stripping directly into plastic zip lock bags. Air pockets were squeezed out and the bags were sealed. The sampled eggs never came in contact with water. The livers were dissected from the spent females and placed in sample containers. Both eggs and livers were immediately frozen and stored at –80 °C until thiamine quantitation.

Atlantic salmon and sea trout. Adult Atlantic salmon was sampled at E1 at the Fisheries Research Station in Älvkarleby, River Dalälven, in the autumn 2006, and at I9 at Mörrums Kronolaxfiske, River Mörrumsån, in the autumn 2011. On the latter occasion, sea trout eggs were sampled as well. Atlantic salmon larvae, produced by artificial fertilization, were sampled at I9 at Mörrums Kronolaxfiske, River Mörrumsån, and at K1 at Statkraft's Salmon Aquaculture Facility in Laholm, River Lagan, in the spring 2012.

Sampling of adult Atlantic salmon and sea trout. Adult Atlantic salmon and sea trout were caught during the autumn in stationary fish traps in the respective rivers. They were kept in large flow-through basins until sampling. Each specimen was taken out of its basin with a hand net. At I9, the live specimen (without sedation) was placed upside down in a semi-open stand made of a plastic pipe filled with water in order to take a blood sample. The blood was drawn from the caudal vein with a heparinized (5000 IE/KY/mL) 2 mL syringe, gently turned several times to prevent the blood from clotting. At both E1 and I9, each specimen was stripped of eggs or milt for artificial fertilization. The specimen was then killed with a blow on the head and weighed to the nearest 10 g. The total weight was calculated by addition of the weight of the stripped eggs or milt. Total length was measured to the nearest 1 cm. Liver and remaining gonad (if any) were dissected. At I9, female gonad weight was calculated as the sum of the weight of the stripped eggs and the weight of remaining gonad, whereas the male gonad weight was not recorded. Hence, in the calculation of somatic indices, we assumed the male gonad to constitute 3% of the somatic weight, a value supported by the literature[67,68]. At I9, pieces of liver, epaxial white muscle, and heart tip, as well as the right half of the brain, were sampled for thiamine quantitation, whereas at E1, only eggs were sampled for thiamine quantitation. The samples were put in cryotubes, submerged in liquid nitrogen, and stored at –140 °C until analysis. For analysis of thiamine-dependent enzymes, a central piece of the liver, as well as the left half of the brain (I9) or the entire brain (E1), were prepared in the same way as for the female European silver eels. At E1, the liver was sampled from the first 22 specimens, whereas the brain was sampled from the remaining 8 specimens. At I9, on the other hand, liver and brain were sampled from all specimens. The precaution to store the samples for enzymatic analysis at –140 °C was specifically motivated by the fact that the thermostability of the apoenzyme has been demonstrated to be lower than that of the holoenzyme for TK in the rainbow trout (*Oncorhynchus mykiss*)[69]. Blood glucose, haemoglobin, and

haematocrit were measured in the same way as for the female European silver eels. The sampling of each specimen was performed without interruption and completed before the next specimen was sampled. Subsequent genetic analysis revealed that some of the specimens at I9 were in fact hybrids between Atlantic salmon and sea trout. These specimens were excluded from the evaluation of data. For sea trout at I9, only eggs for thiamine quantitation were sampled.

Sampling of Atlantic salmon larvae. The Atlantic salmon larvae were sampled at 166–175 day-degrees (d°C) (I9) or 175–180 d°C (K1) post hatch to facilitate comparison with previous investigations of Atlantic salmon larvae sampled at 182 d°C post hatch (E1) by Amcoff *et al.*[70]. At I9, the larvae originated from known family pairs created from the mature Atlantic salmon sampled previously, and accordingly, relationships between the thiamine status of the mature female and her offspring were possible to analyse. For each family pair at I9, the following material was sampled from the larvae: 3–4 pools of 3–4 whole larvae each for thiamine quantitation; 4 pools of 6 livers each for analysis of TK activity and latency; and 1 pool of 12 livers for analysis of KGDH activity and latency. The individual larvae were weighed to the nearest 0.01 g, and total length of the specimens sampled for TK analysis was measured to the nearest 0.25 mm. The pools of whole larvae were put in cryotubes, submerged in liquid nitrogen, and stored at –140 °C until analysis. The livers for TK analysis were homogenized in 500 µL ice-cold 0.25 M sucrose in a 500 µL Potter-Elvehjem homogenizer (size 18) with eight up and down strokes at 400 rpm and under constant cooling with ice-water. The homogenate was diluted with another 100 µL ice-cold 0.25 M sucrose, put in cryotubes, submerged in liquid nitrogen, and stored at –140 °C until analysis of TK activity and latency, which were measured in the homogenate. The livers for KGDH analysis were homogenized in 500 µL ice-cold 0.25 M sucrose in a 500 µL Potter-Elvehjem homogenizer (size 18) with eight up and down strokes at 400 rpm and under constant cooling with ice-water. The homogenate was diluted with another 700 µL ice-cold 0.25 M sucrose, transferred to a 2 mL Eppendorf tube, and centrifuged at 10,000 g_{av} and 4 °C for 10 min in an Eppendorf 5415R centrifuge (Eppendorf, Hamburg, Germany). The supernatant was removed and the pellet in the 2 mL Eppendorf tube was submerged in liquid nitrogen, and stored at –140 °C until analysis of KGDH activity and latency. At K1, the larvae were obtained as a random mixture of several unknown family pairs. From this material the following samples were taken: 4 pools of 3–4 whole larvae each for thiamine quantitation and 6 pools of 6 livers each for analysis of TK activity and latency. These samples were processed in the same way as those of the larvae from I9.

Analysis of thiamine-deficiency-induced mortality in Atlantic salmon larvae. Several Swedish rivers have been exploited for hydroelectric power plants. Because the dams impede spawning migration of Atlantic salmon and sea trout, the hydroelectric power plants must establish at least one facility per exploited river for compensatory breeding of these species to support their stocks in each river. (In River Mörrumsån, there has been no injunction to perform compensatory breeding, but this was done anyway in the years 1985–1997.) Breeding has been performed in family pairs, generally by combining the eggs of one female with the milt from one or two males. Since 1974, thiamine-deficiency-induced mortality in the larvae has been recorded at these compensatory breeding facilities, although with gaps in the time series. The relationship between thiamine deficiency and mortality has been based on typical thiamine deficiency symptoms in the larvae[71]. The number of females with thiamine-deficiency-induced mortality in the larvae, as well as the total number of females, have been recorded each year in each investigated river, and the corresponding frequencies of females with larval mortality have been calculated. These

frequencies for nine rivers are presented here, both for the rivers separately, and as an average of one to nine rivers. Because we have used original data, the time series presented here may differ somewhat from previous compilations of frequencies of females with thiamine-deficiency-induced mortality in the larvae. Data on total length and total weight at E1, which were measured routinely in connection with the compensatory breeding, were used to compare the total weight and somatic condition index (SCI, defined below) between females with and without larval mortality.

Adult herring. Adult herring was caught on their spawning grounds just outside Karlshamn (I8) in the County of Blekinge at the time of spawning in mid-April 2012. The 21 specimens were caught with spinning rods with a jig during three days of intense fishing. On average, four spinning rods were active during 3–4 h each day. Contrary to most other biological material included in this article, the herring material was probably a non-random sample from the population. Firstly, there was a selection of specimens that were healthy enough to migrate to their breeding area at the coast for spawning. Secondly, the catch method was selective of more active specimens capable of catching a jig. After catch, the specimens were directly put in a fish tank with water from the collection site, and transported to the laboratory for sampling within a few hours.

Sampling of adult herring. The adult herring were stunned with a blow on the head, and a blood sample was drawn from the caudal vein with a heparinized (5000 IE/KY/mL) 2 mL syringe, gently turned several times to prevent the blood from clotting. Liver and brain were dissected for thiamine quantitation and analysis of thiamine-dependent enzymes. Because the brain was so small (0.14–0.24 g), it was allocated to either thiamine quantitation or enzymatic analysis in roughly equal proportions of the specimens. Also white muscle was dissected for thiamine quantitation. The sampling was performed in the same way as for the female European silver eels. The sampling of each specimen was performed without interruption and completed before the next specimen was sampled.

Body indices for fish. The somatic weight was defined as the total weight minus the gonad weight. Somatic growth (SG) was defined as the somatic weight (g) divided by the age (years). Heart somatic index (HSI) was defined as the heart weight expressed as percent of the somatic weight; liver somatic index (LSI) was defined as the liver weight expressed as percent of the somatic weight; and gonadosomatic index (GSI) was defined as the gonad weight expressed as percent of the somatic weight. Gonadototal index (GTI) was defined as the gonad weight expressed as percent of the total weight. For Atlantic salmon and herring, somatic condition index (SCI) was defined as 100 times the somatic weight (g) divided by the cubed total length (cm^3)[72]. For European eel, however, the total length was found inappropriate as an allometric standard, because it was probably reduced by thiamine deficiency. As an alternative, we used the brain weight, which has been demonstrated to be relatively constant in rats[73] and mice[74] with and without severe thiamine deficiency. Hence, for the European eel, a body condition index (BCI) was defined as the somatic weight (kg) divided by the brain weight (g). There was a good correspondence between BCI and SG in the European silver eels (Fig. S4j), demonstrating the validity of the new BCI variable.

Thiamine quantitation. Non-phosphorylated thiamine (T), thiamine monophosphate (TMP), and thiamine diphosphate (TDP) were quantitated in the biological material with high performance liquid chromatography (HPLC) with fluorescence detection (Figs. S7a–d). Analysed matrices included: blue mussel soft body and digestive gland; common eider liver, brain, and egg yolk;

domestic chicken liver and brain; European eel liver, brain, white muscle, eggs, and elver decapitated body rest; Atlantic salmon liver, brain, heart tip, white muscle, eggs, and whole larvae; sea trout eggs; and herring liver, brain, and white muscle. The samples were prepared and analysed according to Brown *et al*.[75] with modifications suggested by Kankaanpää *et al*.[76] and with the modification that the derivatization reagent (hereafter reagent), potassium hexacyanoferrate, was prepared in 150 µL of 0.72 M NaOH to a concentration of 0.05%. The method of Brown *et al*.[75] was based on the use of 0.5 g sample and a reagent concentration close to ours. Every new matrix was, however, examined for adequate amount of sample and reagent, and when necessary, the amount of sample and/or reagent concentration were adjusted (described below) to optimize the thiamine quantitation. In the derivatization reaction, thiamine is converted to thiochrome. Samples were filtered through a Chromacol 17-SF-45(T) syringe filter (Thermo Fisher Scientific Inc., Waltham, MA, USA). Thiamine was quantitated with a LaChrom HPLC system with the following components: Interface D-7000, Pump L-1000, Autosampler L-7200, Column Oven L-7350, FL Detector L-7485, Solvent Degasser L-7612 (Merck, Hitachi, Tokyo, Japan). The analysis was performed with fluorescence detection (excitation λ=375 nm, emission λ=433 nm) and chromatographic conditions according to Mancinelli *et al*.[77] except that 85 mM, pH=7.5, potassium phosphate buffer:acetonitrile 45:55 (v:v) was used as eluent under isocratic conditions. Before mixing of the two liquids, the acetonitrile was degassed in an ultrasonic bath (J.P. Selecta S.A., Abrera, Spain). The two liquids were mixed in the HPLC pump after the solvent degasser. Each run was 25 min at a flow of 1 mL/min. The chromatographic column was a Luna® 5µm NH_2 column 250x4.6 mm (00G-4378-E0) with double guard columns (SecurityGuard™ NH_2 4x3.0 mm, AJ0-4302), all from Phenomenex (Torrance, CA, USA). Guard columns as well as chromatographic column were frequently replaced in order to maintain good chromatography. For some matrices (*e.g.* blue mussels), it was necessary to replace the guard columns even before there were any obvious signs of degraded chromatography, in order to save the chromatographic column. Thiamine was quantitated by comparison with external standards. The retention time window compared with the external standard was set to ±0.06 min for T and TMP, whereas the TDP peak was always unambiguous. The respective peaks were also checked visually. The level of detection (LOD) for the method was determined by repeated injections of a standard solution of T, TMP, and TDP into the HPLC system, as described by Boqué & Vander Heyden[78]. This was done using two standard solutions with the respective concentrations 80 nM and 200 nM before clean-up, which gave very similar results, and from which the mean LOD was calculated. LOD for T and TMP was ca 0.01 nmol/g in a 0.5 g sample and ca 0.03 nmol/g in a 0.2 g sample, whereas LOD for TDP was ca 0.03 nmol/g in a 0.5 g sample and ca 0.07 nmol/g in a 0.2 g sample. To check the reproducibility of the method, a homogeneous reference material was prepared from six domestic chicken livers, which were pooled, submerged in liquid nitrogen, manually ground to powder in a mortar, and stored at –140 °C. Before this reference material was consumed, a new reference material was prepared from deer liver and intercalibrated with the previous reference material. The deer liver was submerged in liquid nitrogen and hammered to pieces, which were ground to powder in batches with a CryoMill (Retsch GmbH, Haan, Germany) in a 50 mL cell containing four 10 mm stainless steel balls (3 min pre-cooling and 3 min grinding at 25 Hz). The batches of deer liver were then mixed to homogeneity in liquid nitrogen in a mortar and stored at –140 °C. Coefficients of variation for the analysis of the domestic chicken liver reference material were 23.7% for T, 7.3% for TMP, and 13.6% for TDP, and coefficients of variation for analysis of the deer liver reference material were 9.4% for T, 9.4% for TMP, and 14.8% for TDP. A domestic chicken egg yolk reference material, which was prepared in the same way as the domestic

chicken liver reference material, had a coefficient of variation for T of 13% for analyses performed in 2005–2007 and 6.4% for analyses performed in 2013. SumT was defined as T+TMP+TDP.

Modifications of the thiamine quantitation. In the beginning, thiamine in blue mussels from G1 was quantitated either in single specimens or in pools of two or three specimens. It turned out, however, that the thiamine concentrations obtained from samples weighing more than 0.75 g were systematically too low. When this was discovered, pools of blue mussels were abandoned, and henceforth any blue mussel soft body weighing more than 0.75 g was split into smaller parts analysed separately, and the result was reported as a weighted average for that specimen. Livers from all species were analysed as 0.2 g samples and with a reagent concentration of 0.2%. Common eider egg yolk was analysed as 0.2 g samples taken from a semi-thawed cryotube. In order to avoid error due to potential inhomogeneity within the common eider brain, the right half of each brain was homogenized in 2% TCA and an aliquot corresponding to 0.5 g brain tissue was taken out for further preparation and analysis. For the same reason, the right half of each domestic chicken brain was analysed, and if a sample weighed more than 0.55 g, it was split in two halves analysed separately, and the result was reported as a weighted average for that specimen. For domestic chicken brain samples, a reagent concentration of 0.2% was used. Atlantic salmon heart tip and white muscle were analysed as 0.2 g samples. Atlantic salmon eggs were analysed as pools of three eggs, each pool weighing 0.35–0.55 g. Duplicate egg pools were analysed for each female. Atlantic salmon whole larvae were homogenized and analysed as pools weighing ca 0.5 g. The European eel brain samples weighed 0.07–0.17 g and the herring brain samples weighed 0.14–0.24 g *(i.e.* less than the usual sample weight of 0.5 g). The volume of the homogenization medium was halved for the European eel brain samples, but not for the herring brain samples. In order to maintain the ability to make reliable comparisons with other analyses with the method of Brown *et al*.[75], no other adjustments of the method were made.

Normalization of thiamine concentrations in stored material. For three batches of blue mussels sampled in 2006, half of each batch was analysed in 2007, whereas the other half was stored at –140 °C and analysed in 2013. In the material that had been stored deep frozen for six years, SumT was ca 32% lower than in the corresponding material analysed in 2007. Such degradation with time has been observed previously, at least at temperatures between –20 °C and –80 °C[79]. In order to compensate for the degradation of thiamine during the storage at –140 °C, the SumT concentrations of blue mussels sampled in 2006 and analysed in 2013 were multiplied by a factor of 1.46. Such blue mussels constituted 65% of the blue mussels sampled in 2006. No degradation of T was observed in common eider egg yolk stored at –140 °C for the same time. An explanation of the difference between blue mussel soft body and common eider egg yolk may be that thiamine is less stable in a cellular matrix than in pure egg yolk.

Normalization of thiamine concentrations in blue mussel. In order to rescue some of the thiamine analyses from pools of two or three blue mussels (described above), a standard curve was constructed from measurements in 41 samples of a homogeneous reference material of blue mussels from G1. The sample weights were evenly distributed over the range 0.1–2.0 g. The standard curve revealed that the TDP concentration decreased exponentially with sample weights over 0.75 g. This relationship allowed normalization of the TDP concentrations measured in samples weighing between 0.75 and 1.25 g by the formula:

$$C_{TDPnorm} = C_{TDP} * \exp(1.5 * (weight_{sample} - 0.75)) \quad (1)$$

The constant 1.5 was found empirically to minimize the difference between the normalized TDP values and the (correct) TDP values obtained from corresponding blue mussels analysed at sample weights less than 0.75 g.

Also T and TMP were systematically low in samples weighing more than 0.75 g. For these two forms of thiamine, an empirically determined factor was used to normalize the values by the formulae:

$$C_{Tnorm} = C_{T} * 2.3 \quad (2)$$
$$C_{TMPnorm} = C_{TMP} * 1.2 \quad (3)$$

Normalization with the above formulae was performed in 17% of the T values, 9% of the TMP values, and 18% of the TDP values from G1. The differences in the percentage of normalized T, TMP, and TDP values were due to the unequal numbers of chromatographic peaks that were impossible to quantitate (described below). Moreover, blue mussels with normalized thiamine values were evenly distributed throughout the investigated material, *i.e.* they were not confined to a certain sampling occasion or time of the year. All other analysed blue mussel samples weighed less than 0.75 g.

Estimation of thiamine concentrations in blue mussel and domestic chicken. TDP, which generally constitutes ca 90% of the SumT in blue mussels (Fig. 2c, Fig. S2a), was possible to quantitate in all specimens without any problems (Figs. S7b,c). For T and TMP, however, quantitation was sometimes impossible, owing to interfering chromatographic peaks (Fig. S7c). In such cases, T and TMP concentrations were estimated in order to obtain an unbiased value of SumT (not for the study of T and TMP alone). The estimation formulae were based on those blue mussels, where all three forms of thiamine were possible to quantitate. Missing T concentrations were estimated as 0.4% of the TDP concentration, except at G1, where a constant T concentration value of 0.005 nmol/g was used instead. The rationale of using a constant instead of a percentage at G1 was that no percentage could be determined from the quantitated T values at this station, whereas the mean of these values was 0.005 nmol/g. Missing TMP concentrations were estimated as 11% of the TDP concentration in all cases. Approximately 57% of the T concentrations and 7% of the TMP concentrations in the blue mussels were estimated in this way. For domestic chicken, a few T concentrations were estimated in the same way and for the same purpose (to obtain an unbiased value of SumT, not for the study of T alone).

Normalization of thiamine concentrations in fish eggs. The T concentration in fish eggs may be analysed before or after the eggs have taken up water (water-hardening) after release from the female. In Atlantic salmon, the egg volume typically increases with 25% during this process[80], which is completed within a few hours. When we have analysed SumT in salmonine eggs dissected or stripped directly from the ovary, we have adjusted for the water-hardening by multiplying the SumT concentration by a factor of 0.8. Since we do not know the corresponding factor for anguillid eggs, the egg thiamine concentrations in American eels were not adjusted for water-hardening.

Estimation of thiamine concentrations in fish eggs. No Atlantic salmon eggs were sampled at K1. Hence, the egg SumT concentrations at this station were estimated from the SumT concentrations in the larvae 175–180 d°C post hatch, using the strong correlation between SumT concentrations in newly fertilized eggs and larvae 166–175 d°C post hatch observed at I9 (see Supplementary Results).

Quality aspects on thiamine quantitation. Several practices were applied to ensure a correct quantitation of thiamine. All material sampled for thiamine quantitation was stored at –140 °C in order to minimize dephosphorylation and degradation of the thiamine. For the same reason we kept strictly to the directions and time limits of the method described by Brown *et al.*[75]. This method was chosen because it is the most frequently used method for quantitation of the respective phosphorylated forms of thiamine, and because it has been used by us previously. It thus enables us to make reliable comparisons with many other analyses (including our own previous data). The three forms, T, TMP, and TDP were always analysed separately, even in cases where only SumT was used in the evaluation of data. By this strategy, we were able to monitor the respective proportions of the three forms, and, as a quality control, verify that they were in the expected range for each analysed matrix. Material with higher thiamine concentrations was analysed together with material with lower thiamine concentrations (on the same occasion) to ensure that the observed differences were real. In a large number of cases (ca 30 samples), where the chromatographic peaks of T and TMP were obscured by interfering chromatographic peaks, standard addition was applied to correctly identify the T and TMP peaks in the chromatograms (Fig. S7c). The information obtained in this way facilitated interpretation also of those chromatograms with interfering chromatographic peaks, where standard addition was not applied. Control experiments confirmed that quantitation by standard addition and external standard gave equivalent results (Fig. S7d). With all this work on quality control, we are confident that we have identified the thiamine peaks and quantitated the thiamine concentrations correctly.

Liver/brain SumT ratio. Our investigations so far[1] suggest that the ratio of the liver SumT concentration and the brain SumT concentration is useful as a biomarker of thiamine deficiency in vertebrates. The rationale of this biomarker is that homeostasis in the brain has higher priority than homeostasis in the liver. Hence, thiamine deficiency becomes apparent in the liver before it becomes apparent in the brain. The liver/brain SumT ratio has been 2–3 in non-thiamine-deficient vertebrate specimens, and as low as below 1 in thiamine-deficient specimens[81–83]. At more severe thiamine deficiency, however, the liver/brain SumT ratio may start to increase, because liver SumT cannot decrease any further, and therefore remains relatively constant, while the brain SumT starts to decrease with increasing thiamine deficiency. This was *e.g.* the case in the common eider females in the County of Blekinge (region I) compared with those in Eastern Iceland (region D) in the present investigation. Another exception to the rule was the non-thiamine-deficient herring in the present investigation, which had remarkably high brain SumT concentrations and, as a result, a liver/brain SumT ratio below 1, whereas the other analysed biomarkers did not indicate thiamine deficiency in these specimens. We propose that the liver/brain SumT ratio is useful as a thiamine deficiency biomarker when interpreted with care.

Other forms of thiamine. Intracellularly, thiamine occurs also in the forms of thiamine triphosphate, adenosine thiamine diphosphate, and adenosine thiamine triphosphate. The metabolism of these thiamine forms has been reviewed recently by Bettendorff *et al.*[84], and to date

their physiological role is unknown. Owing to this circumstance and the fact that they generally amount to less than 1% of the total thiamine[84] under normal physiological conditions in the tissues analysed here, we have not reserved specific resources for their measurement in the present investigation.

Analysis of thiamine-dependent enzymes. TDP is a cofactor in thiamine-dependent enzymes, which are essential in the basic metabolism of every living cell. If the cofactor is missing, the thiamine-dependent enzyme is inactive. Thiamine deficiency may thus result in a variety of toxic effects, owing to reduced enzymatic activity. Here, we measured the endogenous activity of the two thiamine-dependent enzymes transketolase (TK) and α-ketoglutarate dehydrogenase (KGDH). TK activity was measured in the digestive gland (blue mussels) and the liver and brain (vertebrates), whereas KGDH activity was measured only in the liver and brain. The endogenous activity reflects the amount of thiamine-dependent enzyme with the cofactor originally present in the sample. We also measured the maximum activity, which is obtained after addition of excess TDP to the sample. If the sample contains enzyme without the cofactor (apoenzyme), the supplied extra cofactor binds to the apoenzyme and thereby turns it into a fully active holoenzyme. Hence, the difference between maximum and endogenous activity reflects the original amount of enzyme where TDP was missing. Latency is this difference expressed as percent of the maximum activity, and has been demonstrated[85] to be a better measure than TPP-effect, where this difference is expressed as percent of the endogenous activity. Theoretically, the maximum activity is always higher than or equal to the endogenous activity. A maximum activity slightly lower than the endogenous activity may occur, however, but is a result of method uncertainty. To avoid positive bias, the resulting negative latencies were used in all calculations and figures, whereas in the tables, they were truncated in the reporting of the final result. Both endogenous activity and latency are two important biomarkers of thiamine deficiency. All activities of the thiamine-dependent enzymes reported here are endogenous activities, unless otherwise stated. TK activity was measured with a Hitachi U-3200 UV/Vis Spectrophotometer (Hitachi, Tokyo, Japan) or a Shimadzu UV-2600 UV-Vis Spectrophotometer (Shimadzu Corporation, Kyoto, Japan) in the 10,000g_{av} supernatant. (For Atlantic salmon larvae, however, TK in the liver was measured in the homogenate.) The measurement was performed in 100 mM Tris-Cl buffer, pH=7.6, at 30 ºC (blue mussels and fish) or 25 mM Tris-Cl buffer, pH=7.0, at 40 ºC (birds), and otherwise according to Tate & Nixon[86]. For measurement of maximum activity, TDP was added to the cuvette to a final concentration of 100 μM. The results were expressed as nmol NADH consumed per min and mg protein, which was quantitated according to Lowry *et al.*[87] with bovine serum albumin as the standard. KGDH activity was measured with a Shimadzu UV-2501PC UV-Vis Spectrophotometer (Shimadzu Corporation, Kyoto, Japan) or a Shimadzu UV-2600 UV-Vis Spectrophotometer (Shimadzu Corporation, Kyoto, Japan) in the re-suspended 10,000g_{av} pellet. The measurement was performed at 25 ºC (all species) according to Lai & Cooper[88] and Lai *et al.*[89]. For measurement of maximum activity, TDP was added to the cuvette to a final concentration of 200 μM. The results were expressed as nmol NADH formed per min and mg protein, which was quantitated according to Lowry *et al.*[87] with bovine serum albumin as the standard. All measurements were carried out in duplicate, the enzyme activities were demonstrated to be linear with time and amount of protein, and appropriate background and control incubations were performed routinely.

Analysis of ethoxyresorufin-*O*-deethylase (EROD) activity. EROD activity in the 10,000g_{av} supernatant was measured with a Jasco FP-777 Spectrofluorometer according to the method

described by Prough *et al*.[90]. The results were expressed as pmol resorufin formed per min and mg protein, which was quantitated according to Lowry *et al*.[87] with bovine serum albumin as the standard. The measurements were carried out in duplicate, the enzyme activities were demonstrated to be linear with time and amount of protein, and appropriate background and control incubations were performed routinely.

Histopathology. The common eider and domestic chicken tissue samples for histopathological analysis were dehydrated overnight, embedded in paraffin, and cut in 4 µm sections collected on microscope slides. The sections were stained with haematoxylin and eosin (H&E) and Luxol fast blue, and examined microscopically. The microscope image was taken with a Nikon ECLIPSE 80i microscope with a Nikon Digital Sight DS-Fi1 video camera coupled to NIS-Elements D 4.13.03 image analysis software (all from Bergman Labora, Danderyd, Sweden).

Data analysis. Statistical analysis was made with the softwares StatView 5.0 (SAS Institute Inc., Cary, NC, USA) and Intercooled Stata 12.2 (StataCorp LP, College Station, TX, USA), and included analysis of variance (ANOVA), regression models, the Wald test, the Student's *t*-test, the Z-test, the Pearson correlation, and the Wilcoxon-Mann-Whitney test. When an analysis included pools of blue mussels, each pool was given a weight corresponding to the number of specimens in the pool. The Shapiro-Wilk normality test and the Breusch-Pagan/Cook-Weisberg test for heteroscedasticity, as well as diagnostic plots, were used to ascertain that assumptions of normality and homoscedasticity were met. Only 2-tailed tests were used. *P*-values below 0.05 were considered significant. One star (*) denotes $P<0.05$, two stars (**) denote $P<0.01$, and three stars (***) denote $P<0.001$. Only biological (not technical) replicates were used here, *i.e.* the number of observations corresponds to the number of analysed specimens.

In the bar and line graphs, the bars and markers represent arithmetic means, and the whiskers represent 95% confidence intervals (CI). Also in the text, values are given as arithmetic mean±95% CI. In the box plots, the box represents the quartiles: Q1, Q2, and Q3. Two fences are defined as $Q1-1.5\times(Q3-Q1)$ and $Q3+1.5\times(Q3-Q1)$. Whiskers are drawn extending from the ends of the box to the most extreme values that are still inside the fences. Observations that fall outside the fences are regarded as possible outliers and are indicated by dots[91].

Demonstration of bivariate linear relationships was carried out by ordinary least squares (OLS) regression, because of its versatility and easily interpretable *P*-value for the slope and coefficient of determination (R^2). When both y and x are subject to error, however, OLS regression slopes are more or less biased[92]. The problem becomes apparent as a less good fit of the regression line to the data. In such cases, especially when we were interested in the intercept, we performed geometric mean (GM) regression as a complement to the OLS regression. GM regression gives an unbiased slope and intercept when both y and x are subject to error[92]. When necessary, 95% CI for the GM regression intercepts were computed by bootstrapping with 100,000 replications per computation.

Because blue mussel length classes (small, medium, large) sometimes differed in SumT concentration, length class was included as a confounding variable in the blue mussel SumT comparisons between areas and sampling occasions. Means and 95% CI were reported as predicted values for medium blue mussels, *i.e.* as if all specimens were of medium length.

In the graphs, colours were used in the following way: Baltic Sea area – red; Atlantic Ocean – blue; Great Lakes area – pink; Pacific Ocean – grey; and control groups – green. Orange and violet were used for multiple ends.

Supplementary Results

Blue mussel (*Mytilus sp.*)

Blue mussels are widespread within the Northern Hemisphere, and they are the main primary consumer in the Baltic Sea, where they also constitute important prey for several birds and fish, *e.g.* a number of diving ducks (including the common eider), flatfish, the European eel[34,93], and the eelpout (*Zoarces viviparus*). Recently, also the roach (*Rutilus rutilus*) has been demonstrated to eat blue mussels[94]. A few decades ago, blue mussels were very common on the hard bottoms of the Baltic Sea[95] and completely dominated its animal biomass (90%) and production[96]. Their filtration of organic particulate matter has been recognized as a key function of the entire ecosystem[97–99]. Knowledge is limited, however, about the thiamine production in the primary food of the blue mussels, *i.e.* the phytoplankton[100], and further research is needed to elucidate current relationships in the aquatic thiamine production.

To the best of our knowledge, there is no quantitative investigation of the density change of the Baltic Sea blue mussel population over the last decades. In region G, however, environmental monitoring of the blue mussel biomass indicated a decline at three out of six investigated stations 1993–2010[101]. These findings agree with our own observations that the blue mussels have disappeared, more or less, from many places in the Baltic Sea and Iceland, where they previously were highly abundant. Blue mussel population declines have also been reported at other places in the Northern Hemisphere, such as the Romanian Black Sea coast[102], the Wadden Sea[103–105], eastern England[106], the Connecticut coast[107], the US west coast[108–110], and Japan[111]. Other Northern Hemisphere bivalves with mortality that may, at least in part, be related to thiamine deficiency include *Chamelea gallina* in the Adriatic Sea[112], various species in the Dutch Wadden Sea[103,113], cockle (*Cerastoderma edule*) in eastern England[106], Iceland scallop (*Chlamys islandica*) in Iceland[114], and barnacle (*Balanus glandula*)[110] and Pacific oyster (*Crassostrea gigas*)[115] at the US west coast. Blue mussels and other bivalves have a well-developed immune defence[116,117], which may be suppressed by thiamine deficiency. Hence, infection of various pathogens may be related to thiamine deficiency. In recent decades, several infections in bivalves have been reported in various parts of the Northern Hemisphere, including Pacific oyster at the French Mediterranean coast[118] and the French west coast[119], Iceland scallop in Scottish, Faroese, and Icelandic waters[120], Japanese scallop (*Mizuhopecten yessoensis*) in Japan[121], and Akoya pearl oyster (*Pinctada fucata*) in Asia[6]. It has been concluded that temperature and reproductive effort alone are insufficient to cause summer mortality in Pacific oysters[122]. Additional stressors, such as *e.g.* opportunistic pathogens, are necessary to cause this mortality[122].

Soft body SumT concentration sometimes differed between the length classes small, medium, and large (Table S2a). When there was a difference, larger specimens had lower SumT than smaller specimens. This finding suggests that thiamine deficiency increases with age. The mechanism for this phenomenon may be that older blue mussels have been subject to more episodes of thiamine deficiency, which may have resulted in more or less permanent damage[123–126]. Another possibility is that the size of filtered food particles is correlated with the size of the blue mussels, and that the food of smaller mussels thus contains a larger proportion of bacteria and fungi, which may contain more thiamine than the phytoplankton. To facilitate comparison between areas and sampling

occasions, SumT data were normalized by statistical means and presented as if all blue mussels were of medium length.

Thiamine deficiency disease may be due to malfunction of the thiamine phosphorylation within the cells. Such malfunction has previously been observed to be caused by genetic factors[127], metabolic factors[128,129], as well as xenobiotic compounds[130,131]. Our data, however, suggest normal function of the thiamine pyrophosphokinase[132], because the proportions of T, TDP, and TMP were very similar in specimens from Iceland and the Baltic Sea, differing in SumT levels (Fig. 2c, Fig. S2a).

The blue mussels in Fig. S2b were experimentally subjected to various times of hypoxia and hyperthermia, a way to artificially induce thiamine deficiency in the laboratory. The negative relationship between digestive gland TK activity and latency (Fig. S2b) verifies, for the first time, the usefulness of these thiamine deficiency biomarkers also in blue mussels. It is noteworthy that the digestive gland TK activity seems to have a general lower limit of 5–6 nmol/min/mg (Fig. 2e, Figs. S2b,c,e–g). This limit may be a threshold, below which long-term survival is impossible. It is also interesting that even the highest digestive gland TK activities (Fig. S2e) were within a factor of only 2.5–3.0 of this threshold. Accordingly, the margin for survival of the majority of individuals seems to be small today.

A negative relationship between digestive gland TK activity and latency was observed at A4 (Fig. S2e) and in three of four seasons at G1 (Fig. 2e). No corresponding relationship was observed at B1 in week 42 (Fig. S2f), C2 in week 41 (Fig. S2g), or G1 in the summer (Fig. 2e). It is noteworthy that the negative relationship between digestive gland TK activity and latency was absent only when SumT concentrations were among the lowest observed in this investigation (Figs. 2a,b; additionally (not shown), SumT at B1 week 42 was 1.0±0.1 nmol/g), *i.e.* when thiamine deficiency was particularly severe. Part of an explanation may be that individuals with higher TK activities are missing under such conditions (Fig. S2h). Possibly, TK latency decreases at particularly severe thiamine deficiency, because the cells no longer maintain the apoenzymes. This would result in an increased number of individuals with both low TK activity and low TK latency. If this is the case, no negative relationship between TK activity and latency will appear.

An indication of what the TK activity might be in a non-thiamine-deficient individual is obtained from extrapolation to zero latency in the GM regression in Fig. S2e, which illustrates the group with highest thiamine status (A4 week 25) of those groups where TK activity and latency was analysed. This intercept takes the value 12.5±1.3 nmol/min/mg and should be regarded as a minimum, because an individual may suffer from thiamine deficiency even at zero latency if TK latency is not the most sensitive of all thiamine deficiency biomarkers. An indication of what the soft body SumT concentration might be in a non-thiamine-deficient individual is obtained in Fig. 2b, where the highest mean value was 3.4 nmol/g. Also this value should probably be regarded as a minimum, because SumT values up to 7.6 nmol/g have been observed in individual blue mussels. Extrapolation to zero latency in Fig. 2d and Fig. S2d gave a SumT concentration of 2.2–2.4 nmol/g. This is lower than 3.4 nmol/g, but still higher than the SumT concentrations of most blue mussels today. Extrapolation to a SumT concentration of 3.4 nmol/g in Fig. S2c gave a TK activity of ca 12.9±4.5 nmol/min/mg (not shown), which agrees well with the GM intercept in Fig. S2e.

Fig. S2k illustrates the strong positive linear relationship between dry body weight and wet weight based BCI for the two length groups compared in Fig. 2g. Accordingly, wet weight based BCI is a valid alternative to dry weight based condition variables. A comparison of BCI levels between the three Baltic Sea regions investigated here was made in Fig. S2l. Mean BCI values were in the range 0.20–0.24 g/cm^3, and region F had somewhat lower BCI than region G and I (Fig. S2l). It is noteworthy, however, that all data in Fig. S2l were from weeks 41–43 (the only time when data were available from all three regions), which was within a period of strong increase in BCI, at least at G1 (Fig. S2m, upper arrow). Hence, the observed lower BCI in region F may be a temporal rather than a geographic difference (Fig. S2l). In any case, regional differences in BCI within the Baltic Sea area were small. It is also noteworthy in Fig. S2m that the strong increase in BCI week 37–42 (upper arrow) followed upon a strong increase in SumT week 32–37 (lower arrow). It may thus be hypothesized that thiamine status must increase before BCI can increase.

Table S2b shows that BCI was positively related to soft body SumT at 9 out of 18 stations (50%) or on 11 out of 44 sampling occasions (25%). Moreover, 9 of the 11 sampling occasions with a positive linear relationship (82%) occurred during September to December, despite a relatively even distribution of sampling occasions between the first and the second half of the year. When all sampling occasions were pooled, there was a strong positive linear relationship (P<0.0001) between BCI and SumT (Table S2b). Obviously, thiamine status affects BCI in the blue mussels, and perhaps most evidently in the autumn. Figs. S2n–q illustrate two examples with a positive relationship between BCI and SumT (week 22 and 51) and two examples without this relationship (week 42 and 43). It is noteworthy that the relationship was absent in the period when BCI was increasing strongly. It may thus be hypothesized that the relationship between BCI and SumT is obscured when these variables are subject to changes, and apparent when they are more stable. Such variability may explain why the BCI curve was uncorrelated with the SumT curve in Fig. S2m, even though the two curves were roughly similar. BCI is probably influenced by many factors that may obscure its relationship with SumT. Possibly, the episodic nature of the thiamine deficiency contributes to the observed pattern, where this relationship sometimes is apparent and sometimes not.

Common eider (*Somateria mollissima*)

Part of our previous investigation of thiamine deficiency in birds[1] focused on the reproduction of the common eider by analysis of eggs and pulli. Yet, to find the causative agent, and its biochemical mechanism, it is necessary to analyse also the egg laying females. The histopathological findings in the Icelandic and Swedish adult female common eiders were consistent with the normal background pathology in this species, and there were no indications of serious infectious diseases. This is in accordance with previous investigations of thiamine-deficient Atlantic salmon, where no association was found between the thiamine deficiency and a number of common microorganisms[133]. In this context, it should be pointed out that the immunosuppression of thiamine-deficient individuals makes them more susceptible to various pathogens than non-thiamine-deficient individuals. Hence, the observation of a few stray pathogens in analysed specimens provides no evidence that these particular pathogens are the primary cause of the disease.

Because the common eider does not lend itself to a life at the laboratory, we used domestic chicken as a surrogate control. To mimic the field situation, with assumed variation in the thiamine status of the common eider females, the thiamine "complete" and "deficient" domestic chicken groups in Figs. 3a–f and Figs. S3a,b were composed of two dose groups each. The thiamine "complete" group combined the dose groups 10 and 25 (mean 17.5) nmol T per g dry fodder, whereas the thiamine "deficient" group combined the dose groups 0.9 and 1.5 (mean 1.2) nmol T per g dry fodder. Table S3a shows that an average Baltic Sea common eider, feeding on blue mussels, obtains a daily SumT intake comparable to that of the lower dose groups (0.9–1.5 nmol per g dry fodder) of the domestic chicken. This finding suggests that the thiamine deficiency in the common eider primarily is a result of low thiamine concentration in its main prey, the blue mussel.

The low daily SumT intake of the common eider agrees well with the dramatic population declines observed for the Baltic Sea common eiders in recent years[134–136] (Fig. 3h). These declines cannot be explained by factors of more temporary character, such as toxic algal blooms[*e.g.* 137], certain predation[*e.g.* 138], or extreme weather. In favour of the thiamine deficiency hypothesis is also the fact that the populations of other diving duck species feeding on blue mussels in the Baltic Sea have declined as well[135], even though they differ largely in other biological traits. For example, the common eider visits the Baltic Sea during the summer half of the year, whereas the long-tailed duck (*Clangula hyemalis*) visits the Baltic Sea during the winter half of the year. Moreover, the common eider population has declined also in Iceland during the last 15 years, as determined by a yearly survey of common eider nests in 17 colonies, and the decline was more pronounced in some of the investigated regions[139]. This development has been paralleled by a more general population decline in several Icelandic seabird species[140–142].

The dose-response curve for proportion liver TDP in the domestic chicken was clearly biphasic (Fig. S3c). A decrease in dose from 10 to 1.5 nmol per g dry fodder resulted in an increase in proportion liver TDP, whereas a further decrease in dose from 1.5 to 0.3 nmol per g dry fodder resulted in a decrease in proportion liver TDP (Fig. S3c). This latter part of the dose-response curve (0.3–1.5 nmol per g dry fodder) was statistically significant by linear regression (P=0.0078). The same biphasic response pattern has been observed also in thiamine deficient rats[143]. The two phases of the dose-response curve for proportion liver TDP may be interpreted in the following

way. At moderate thiamine deficiency the cells try to keep as much thiamine as possible as TDP, which is the form active as a cofactor in the thiamine-dependent enzymes. The proportion TDP increases with thiamine deficiency at the expense of the proportions of TMP and T. At more severe thiamine deficiency, T and TMP cannot decrease any further, and then the proportion TDP begins to decrease with increasing thiamine deficiency. We propose this biphasic relationship as a useful indicator of thiamine deficiency.

In the domestic chicken dose groups 1.5–10 nmol per g dry fodder, there was a negative relationship between proportion TDP and T concentration in the liver (Fig. S3d) and brain (Fig. S3e), and the same relationship was observed in the common eider females (Figs. S3f,g). A corresponding negative relationship between proportion TDP and T concentration has also been observed in human blood[144]. Thiamine deficiency was further indicated by the negative relationships between activity and latency for liver TK (Fig. S3h), liver KGDH (Fig. S3i), and brain KGDH (Fig. S3j) in the common eider females. The negative relationship between liver KGDH activity and latency differed between Iceland and Sweden (Fig. S3i). This difference may be a result of different histories of thiamine deficiency between the two groups, as discussed below.

Four biomarkers that very clearly demonstrated thiamine deficiency in the common eider females were KGDH activity and latency in the liver and brain (Figs. 3c–f, Figs. S3i,j). Such effects on KGDH have previously been associated with a decrease also in its maximum activity[145–147]. Hence, it is noteworthy that the maximum activity of this enzyme in the liver was lower in the common eider females with at least one dead offspring (before hatch) than in those without dead offspring (Fig. S3k). This is another indication that the thiamine status of the female affects the breeding outcome, even in a material where all individuals suffer from severe thiamine deficiency, and even though offspring mortality was analysed only in the eggs.

The inventories of common eiders at I11 in the County of Blekinge during the breeding seasons 2011–2013 revealed a consistently low number of surviving pulli. The average number of eggs in a clutch was ca 4.0 (mean of three years), which was lower than ca 4.6 reported before the 1970s[148,149]. The station hosted an average of ca 300 females each year, and by simple multiplication, there should be ca 1,200 pulli on the water shortly after hatch, but only 5–10% of this number was observed. The number of females without pulli shortly after hatch was 5–10 times the number of females with pulli.

From the common eider data available here, it is difficult to estimate what the thiamine concentrations and thiamine-dependent enzyme activities should be in non-thiamine-deficient individuals. The usual method to extrapolate to zero latency in relationships between latency and another thiamine deficiency biomarker may not be applicable here, because the regression line may be biased by the thiamine deficiency. A good example of this is the two different liver KGDH relationships in Fig. S3i, one for Iceland and one for the Baltic Sea area. They give extrapolated liver KGDH activities of 20.3±5.0 and 28.3±3.2 nmol/min/mg, respectively, at zero latency. It is unlikely, however, that the true KGDH activity in non-thiamine-deficient individuals should differ so much between the two countries. Moreover, the liver KGDH activity in the domestic chicken complete group was even higher, 40.1±2.3 nmol/min/mg (Fig. 3c). For common eider thiamine concentrations, there are no correlations with latency available in our material (further discussed

below). We just know that liver SumT was about one third of that in the domestic chicken complete group (Fig. 3a).

Liver EROD activity was positively related to thiamine deficiency in Icelandic common eiders (Figs. S3l,m, Table S3b). Liver TDP, liver SumT, liver/brain TDP ratio, liver/brain SumT ratio, endogenous brain TK activity, and maximum brain TK activity were all negatively related to EROD activity (Table S3b). The proportion liver TMP was positively related to EROD activity, whereas the proportion liver TDP was negatively related to EROD activity (Table S3b). The latter two observations indicate that the Icelandic common eiders suffer from a degree of thiamine deficiency corresponding to the left-hand part of the dose-response curve in Fig. S3c, where proportion TDP decreases with increasing thiamine deficiency. This finding is, in fact, compatible with the observations in Fig. S3f, because most of the Icelandic specimens are found to the left, and the negative relationship between the proportion liver TDP and the liver T concentration is dependent mainly on the Swedish specimens. The proportion liver TMP is the main complement to the proportion liver TDP, and consequently, the linear relationship between proportion liver TMP and EROD activity has the opposite sign to the linear relationship between proportion liver TDP and EROD activity.

EROD activity, strongly associated with the CYP1A subfamily of cytochrome P450 enzymes[150–152], is one of the most commonly used biomarkers of exposure to classic persistent organic pollutants (POPs), such as polycyclic aromatic hydrocarbons (PAHs), dioxins, and planar polychlorinated biphenyls (PCBs)[153,154]. Caution should be used, however, when studying individuals close to their reproduction period, as in the present investigation, because of the well-known fact that EROD activity is altered by oestrogen and other oestrogenic substances[155,156]. Still, the positive relationship between EROD activity and thiamine deficiency in the Icelandic common eiders raises the obvious question if classic POPs may be responsible for the thiamine deficiency. The answer is most probably no, because no corresponding relationship was observed in the Swedish common eiders (Table S3b), and because the exposure to classic POPs in the environment does not vary episodically like the occurrence of thiamine deficiency (*e.g.* Figs. S5t,u for Atlantic salmon presented below). Also the negative relationship between EROD activity and LBI (Fig. S3n) speaks against classic POPs as the cause of the thiamine deficiency. If classic POPs were the cause, no or a positive relationship would be expected instead.

EROD induction by thiamine deficiency has, in fact, been suggested previously in Atlantic salmon[157]. Moreover, laboratory studies have shown that thiamine deficiency alone may cause induction of cytochrome P450 enzymes[158]. Grosse III & Wade[159] showed that laboratory rats given a thiamine-deficient diet for three weeks developed both high NADPH-cytochrome P450 reductase activity, as well as increased amount of cytochrome P450 enzymes in the liver, measured according to Omura & Sato[160]. This was observed despite decreased enzymatic markers of the hexose monophosphate shunt. Increased activity of the cytochrome P450 activity N-nitrosodimethylamine demethylase, as well as an increased amount of the isozyme CYP2E1, were also observed when rats were fed thiamine-deficient diets during three weeks[161,162]. Additional cytochrome P450 activities induced in connection with thiamine deficiency analysed *in vitro* include oxidation of aminopyrene, ethylmorphine, and N-methylaniline, as well as hydroxylation of acetanilide and aniline[163,164]. Effects of thiamine-deficient food on enzymatic variables may result from the thiamine deficiency *per se*, or from reduced feeding owing to loss

of appetite, or a combination of the two[165,166]. Moreover, it has been shown that induction of P450 enzymes by well-known inducers, such as phenobarbital and 3-methylcholanthrene, may be stronger in thiamine-deficient individuals[166,167]. Enhanced metabolism and excretion of paracetamol were also observed *in vivo* in thiamine-deficient rats[168]. Taken together, these findings suggest that depuration of various xenobiotics may increase in thiamine-deficient animals. This is not only positive, however, since the biotransformation of xenobiotics is always associated with a risk for injury. Increased depuration by thiamine deficiency should also be taken into account in investigations of biota in the field today, especially when long-term monitoring of xenobiotic levels in such biota show decreasing temporal trends.

An obvious question is why a positive relationship between EROD activity and thiamine deficiency was observed in Iceland but not in Sweden (Table S3b). The Baltic Sea area is more polluted by classic POPs than Iceland[169,170], and maybe the relationship between EROD activity and thiamine deficiency can only be observed when there is less interference by other EROD inducers. The Swedish common eiders may also be too thiamine-deficient for the EROD activity to respond normally. A possible mechanism for this could be secondary infections and inflammatory responses, which are known to reduce cytochrome P450 activities, including EROD activity[171,172]. It has also been shown in Atlantic salmon that specimens with severe thiamine deficiency lose their EROD activity[173,174].

Although it is possible that EROD induction may be caused by thiamine deficiency, as indicated in the Icelandic common eiders from a relatively unpolluted area, it should be remembered that, so far, the isozyme that has been demonstrated to increase by thiamine deficiency is CYP2E1[162], which typically catalyses the demethylation of N-nitrosodimetylamine, but has not been associated with EROD activity[150,175,176]. To the best of our knowledge the mechanism behind this CYP2E1 induction from thiamine deficiency is not known in detail. Individual cytochrome P450 enzymes have distinct but more or less broad substrate specificities, which in many cases overlap with one another, and Kelley *et al*.[177] showed that a synergism between CYP2E1 and CYP1A2 could occur in reconstituted systems with NADPH-cytochrome P450 reductase. Regulation attributes of CYP2E1 and members of the CYP1A subfamily highlights the complexity of these enzymes.

Another possible mechanism for EROD induction by thiamine deficiency is inhibited thiamine-dependent α-oxidation of certain fatty acids[178,179], whereby their metabolism may be shunted to ω-hydroxylation instead. For example, inhibited α-oxidation of the highly toxic phytanic acid[180–182], which is a degradation product of chlorophyll, may result in an urgent need for ω-hydroxylation of this molecule to facilitate its subsequent β-oxidation. This ω-hydroxylation is performed by the CYP4 family of cytochrome P450 enzymes[183,184]. Again, however, we have found no information in the literature about EROD activity of the CYP4 subfamily members. An alternative effect of thiamine deficiency on EROD activity could be decreased body lipid reserves, as described below for the European and American eel, and release of classic lipophilic EROD inducers when the body lipids are consumed.

The data presented here demonstrate severe thiamine deficiency in common eider females in both Iceland and in Sweden. When there was a difference between these countries, the thiamine deficiency was mostly more severe in Sweden (Figs. 3b,d–f, Figs. S3i,j, Table S3c). The lower liver/brain TDP and SumT ratios in Iceland than in Blekinge (Table S3c) were due to higher brain

TDP and SumT levels (Student's *t*-test $P<0.001$, not shown) in Iceland than in Blekinge, whereas liver TDP and SumT levels were the same in the two countries (Table S3c). This observation suggests that liver thiamine has reached a lower limit in both Blekinge and Iceland, whereas brain thiamine still may reflect differences in thiamine status. It is noteworthy that the liver/brain SumT ratio was below one in both countries, which, in itself, is a strong indication of severe thiamine deficiency. The higher LBI in Sweden than in Iceland (Table S3c) may also be due to more severe thiamine deficiency in Sweden. Experiments, in which rats were fed a thiamine-deficient diet, have shown that LBI decreases in the thiamine-deficient group compared with control when fed *ad libitum*[73,185], whereas LBI increases in the thiamine-deficient group compared with a pair-fed control[73,186]. Abnormal increase in liver size has also been observed in humans at advanced stages of thiamine deficiency[187]. It may thus be hypothesized that LBI decreases at more moderate thiamine deficiency, mainly due to reduced feeding owing to loss of appetite, whereas at more severe thiamine deficiency, LBI starts to increase. This would be in line with both the observed negative relationship between LBI and EROD activity in Iceland (Fig. S3n) and the higher LBI in Sweden than in Iceland (Table S3c). The finding of more severe thiamine deficiency in Swedish than in Icelandic common eiders agrees with our previous investigation of thiamine deficiency in birds[1], where we observed severe thiamine deficiency in the Baltic Sea area and incipient thiamine deficiency in Iceland. The more severe thiamine deficiency in Sweden than in Iceland also adds another argument against classic POPs as the cause of the thiamine deficiency. If classic POPs were the cause, we would expect to find higher EROD activity in Sweden than in Iceland, but we observed the opposite, albeit the difference was small (Table S3c).

The data on Icelandic and Swedish common eiders presented here suggest that their thiamine status has only a small margin to the threshold for survival. It should also be remembered that the biomarker responses depend both on past thiamine deficiency, which may have occurred episodically during many years, and on present thiamine deficiency, which depends on the availability of thiamine at the time close before the sampling. Past thiamine deficiency may occur in combination with present thiamine deficiency or sufficiency, and correspondingly, past thiamine sufficiency may occur in combination with present thiamine deficiency or sufficiency. Such differences in the history of thiamine deficiency between the Icelandic and Swedish common eiders may partly obscure the biomarker responses. With this in mind, the thiamine deficiency biomarker response pattern was yet remarkably clear.

Histopathology of the liver, myocardium, kidney, pancreas, intestine, lung, ovary, thigh muscle, and peripheral nerves and ganglia was investigated in the 12 Icelandic and in ten of the 23 Swedish adult female common eiders. In the majority of specimens from both areas, mild periportal and perivascular mixed cell infiltration was present in the liver. Mixed cell infiltration in the kidneys was observed in four Icelandic and three Swedish specimens, probably indicating coccidian infestation. Four Icelandic and two Swedish specimens had a mild inflammatory reaction in the pancreas. Intestinal nematodes were observed in three Icelandic and one Baltic specimen(s). No signs of inflammation, infection or parasite infestation were observed in the other organs, and there were no degenerative or other changes in the peripheral nerves or ganglia. These histopathological findings were consistent with the normal background pathology in the common eider, and the degree of this background pathology did not differ between Iceland and Sweden.

There are very few scientific publications describing the histopathology of thiamine deficiency in birds. In an article from 1940, pigeons with experimentally induced thiamine deficiency were reported to show peripheral nerve degeneration, chromatolysis of neuronal cell bodies in dorsal root ganglia, and degeneration of spinal cord white matter[188]. Some of the pigeons also showed focal necrosis and inflammation in the heart[188]. In an article from 1972, domestic chicken, fed a thiamine-deficient diet, developed severe neurological signs[189]. There were, however, no histopathological lesions in the central or peripheral nervous system[189]. In an article from 2002, thiamine-deficient honeyeaters, maintained in an Australian wildlife sanctuary, displayed neuropil vacuolation, haemorrhage, and neuronal necrosis in the brainstem[190]. The majority of the honeyeaters were found dead without preceding clinical symptoms, and histopathological brain lesions were present in only one third of the specimens[190]. The observed differences in the histopathological manifestation between these three cases may be related to species or the degree and/or duration of the thiamine deficiency.

Because of the scarcity of previous histopathological investigations of thiamine-deficient birds, we produced nine thiamine-deficient domestic chickens in an experiment, where the brain, liver, myocardium, kidney, and calf muscle were sampled for histopathological analysis. Five of these specimens displayed minimal to moderate neuropil vacuolation in the cerebellar deep nuclei, whereas no other parts of the brain were affected. The vacuoles varied in size from small bubbles to large, empty spaces of the same size as a neuronal cell body (Fig. S3o). One specimen showed similar neuropil vacuolation in the lateral geniculate body of the brainstem, but not in other parts of the brain, such as the cerebral cortex, the cerebellar cortex or the hippocampus. There was no haemorrhage in any of the brains, and no demyelination. Two specimens displayed focal necrosis and inflammation in the myocardium, and similar alterations were also present in the calf muscle in one of these, as well as in a third specimen. The neuropil vacuolation observed in the cerebellar deep nuclei and brainstem of these domestic chickens was similar to some of the histopathological findings in thiamine-deficient honeyeaters[190]. Just like in the honeyeaters, histopathological alterations in the brain were absent in some of the domestic chickens. This finding was expected and suggests that histopathological alterations are secondary to subcellular alterations due to thiamine deficiency, since subcellular alterations were present in all thiamine-deficient domestic chickens and in a large majority of the common eiders. The observed alterations in the domestic chicken brains were consistent with those described in other thiamine-deficient animals (*e.g.* cats and foxes). Typically, neuropil vacuolation, haemorrhage, and/or neuronal necrosis are observed in the lateral geniculate body and other periventricular nuclei of the brainstem[191]. Although the brains of the 12 Icelandic and the ten Swedish adult female common eiders were not available for histopathological analysis, we had access to the brain of another adult common eider from region I. This specimen displayed clinical symptoms of thiamine deficiency and abnormal behaviour, such as walking around aimlessly on an inland highway (wrong habitat) fearless of humans, but without outer or inner injuries. Chemical analysis of this specimen also revealed a liver SumT concentration of 3.7 nmol/g and a brain SumT concentration of 5.9 nmol/g, which is substantially lower compared with most of the other common eiders investigated here. The brain of this extra common eider displayed minimal neuropil vacuolation, which may correspond to the histopathological findings in the thiamine-deficient domestic chickens. In this context it should also be mentioned that we have observed dozens of common eiders in the inland of this region, *i.e.* wrong habitat.

The cerebellar deep nuclei (notably affected in the domestic chickens) are with few exceptions the sole sources of output from the cerebellum, and their projections are widespread throughout the brainstem[192]. Consequently, damage to this area will result in disturbed regulation of muscle tonus, coordination of movements, and maintenance of balance. It is thus very interesting that the clinical symptoms of the domestic chickens included *i.a.* loss of strength in the legs and imbalance. They easily fell down on one side, or backwards, and were not able to get up again. They also showed faintness, and just wanted to lie under the radiator (artificial mother). All these symptoms were consistent with cerebellar dysfunction. Compared with many other animals, birds have more sophisticated cerebellar and vestibular systems, since they have to deal with complex movements in three dimensions[193]. The behavioural manifestations of lesions in the cerebellar deep nuclei may thus be more critical to birds than to many other animals. It has also been observed that cerebellar disorders tend to affect the dominant types of movement in animals, *i.e.* in birds that fly much, the clinical symptoms relate mainly to flight, in birds that swim, the swimming is impaired, and in walking animals, the gait is affected[194]. Possibly, thiamine-deficient birds will perform less well, when they have to cope with hard winds and avoid physical obstacles (*e.g.* wind turbines).

Altered behaviour may be manifested also in other ways. It is well known that common eiders furnish their nests with plenty of down. Hence, it is noteworthy that a normal amount of nest down was observed only in 67% of the nests in Iceland and 52% of the nests in Sweden, whereas the rest of the nests contained less or much less down than normal, according to our classification. Moreover, during the inventories of common eiders at I11 (described above), we found that 2–15% of the nests of several bird species, including the common eider, the barnacle goose (*Branta leucopsis*), the herring gull (*Larus argentatus*), the mallard (*Anas platyrhynchos*), the Canada goose (*B. canadensis*), and the mute swan (*Cygnus olor*), contained eggs of other species[195]. Many nests also contained odd items, such as stones and bits of plastic waste, which the birds apparently had included in the clutch[195]. During the inventories, we also observed pulli with abnormal behaviour, *e.g.* when attacked by a gull, they neither dived nor ran away, and were thus an easy prey to catch[196]. The observed abnormal behaviour indicated severe neurological disturbance, which is well in line with known effects of thiamine deficiency described in the literature, as well as the actual thiamine deficiency demonstrated in the females (this investigation) and pulli[1].

Anguillid eels (*Anguilla spp.*)

At least three members of the global genus *Anguilla*[197] have declined dramatically since the 1970s: the Japanese eel (*A. japonica*), the European eel, and the American eel[198]. At present, there is no scientifically proved and accepted explanation of these declines[199], although migration barriers in continental waters and silver eel mortality during passage through hydroelectric plant turbines[200], changes in ocean circulation patterns[201,202], over-fishing[202], and dioxin-like pollutants[203] have been suggested as contributing factors. The majority of these factors have, however, been possible to exclude as general or major contributors to the observed declines of anguillid eels[204]. The number of European eel leptocephali returning from the Sargasso Sea has declined by 90–99% since 1980[198], and the species is currently classified as "critically endangered" in "The IUCN Red List of Threatened Species™". Because of its magnitude, this decline must be considered as a threat to the genetic diversity of the species. The steepest decline in the European eel population occurred during the first half of the 1980s[198], and it is interesting that there was a large variation between years in the abundance of elvers and yellow eels in Swedish waters during this time and the preceding decade[205], a pattern consistent with episodically occurring thiamine deficiency. It is also interesting that the declines in the European and American eel populations have been coincident with the appearance of *A. crassus* at the respective continents. The close relationship between the European and American eel makes it interesting to compare their thiamine status on the respective sides of the Atlantic Ocean. The Japanese eel has recently been suggested to spawn in the north-western Pacific Ocean, west of the Mariana Islands[206,207], implying that also this species migrates a long way for spawning.

Although the thiamine status was improved in the elvers from River Severn (L1) that were bathed twice in a 100 mg/L T solution for 48 h each time (Figs. 4b–e, Figs. S4b,c), this treatment was not enough to completely eliminate the thiamine deficiency. The range of liver TK latencies decreased from 0–31% to 1–16%, but no more (Figs. S4b,c). The dose was low, however, compared with similar bathing treatments of Atlantic salmon fry, where T doses up to 2,000 mg/L were used to achieve complete elimination of the thiamine deficiency[70,208]. Also in these Atlantic salmon fry, a T dose of 100 mg/L resulted in only partial improvement of the thiamine status[208,209]. It should be pointed out that uptake of thiamine from the water is an unnatural uptake route, which is insignificant in the field, because natural waters have very low thiamine concentrations, both in marine[210–213] and limnic[214–217] environments. This also explains why such high bathing T doses are required to obtain full effect. The thiamine deficiency observed in the elvers (Figs. 4b–e, Figs. S4b,c) is likely to be less dependent on their previous egg yolk thiamine content, because anguillid eel eggs are only ca 1 mm in diameter, and it is presumed that the yolk is consumed within days up to a few weeks after hatch in the Sargasso Sea. This presumption is supported by observations of captive Japanese eel, in which the yolk was consumed in eight days[218]. This time should be compared with the time it takes for the leptocephali to reach the coast (at least 7–9 months and up to three years). The thiamine status of the leptocephali are thus likely to be more dependent on the thiamine content in the diet, the marine snow[32]. Too low thiamine concentration in the eel eggs may, however, cause mortality or irreversible damage to the eel larvae already in the Sargasso Sea. It is noteworthy that there were no muscle TDP concentrations below 2.7 nmol/g in the elvers (Fig. S4c). This value may represent a threshold for survival at this developmental stage.

The positive relationship between liver TK activity and SumT in the European silver eels at I9 differed between 2012 and 2013, although the ranges of SumT concentrations were almost identical (Fig. S4d). This difference may be a result of different types of eel traps, and thus different histories of thiamine deficiency (as discussed above for the common eider), between the two groups. The silver eels sampled in 2012 had lower TK activity, and may thus have been subject to more severe previous thiamine deficiency.

There was no negative relationship between KGDH activity and latency in the liver or brain in the European silver eels (not shown). For KGDH in the liver, the activity was 30.9±2.1 nmol/min/mg (range 22.4–40.0 nmol/min/mg, n=24) and the latency was 1.1±1.2% (range 0–5.3%, n=24). For KGDH in the brain, the activity was 10.3±0.8 nmol/min/mg (range 7.6–14.4 nmol/min/mg, n=24) and the latency was 3.9±2.1% (range 0–12.3%, n=24).

The relationship between the proportion liver TDP and the liver T concentration in the European silver eels was bimodal (Fig. S4g). The two modes probably reflect the two phases of the dose-response curve in Fig. S3c. The right-hand mode corresponds to the right-hand phase, where proportion liver TDP increases with increasing thiamine deficiency, whereas the left-hand mode corresponds to the left-hand phase, where the proportion TDP decreases with increasing thiamine deficiency. The fact that most silver eels in Fig. S4g had very low concentrations of liver T indicated severe thiamine deficiency in these specimens and may explain the negative relationship between the number of *A. crassus* in the swim bladder and the proportion liver TDP (Fig. 4k).

In the European silver eels, the SG was positively related to the liver SumT (Fig. 4g) and liver/brain SumT ratio (Fig. S4i). There was also a positive relationship between the brain TK activity and the BCI (Fig. 4h). These findings indicate a negative effect of thiamine deficiency on feeding and growth, a phenomenon that has been demonstrated previously in anguillid eels in the laboratory[219,220]. The validity of the variables SG and BCI was demonstrated by their close positive relationship (Fig. S4j).

The liver SumT (Fig. S4k), liver/brain SumT ratio (Fig. S4l), and liver/brain TMP ratio (Fig. S4m) all decreased with increasing age. These findings suggest that the thiamine deficiency increases with age. The mechanism for this phenomenon may be that older silver eels have been subject to more episodes of thiamine deficiency, which may have resulted in more or less permanent damage[123–126]. There was, however, no corresponding negative relationship between white muscle SumT and age (Fig. S4k). This finding may be explained by the fact that the white muscle SumT range was 1.0–2.7 nmol/g, which is a low level, perhaps a threshold for survival.

The haematocrit has been demonstrated to decrease in connection with thiamine deficiency in the European eel[221]. Hence, the positive relationships between haematocrit and TK activity in the liver (Fig. S4n) and brain (Fig. S4o), as well as the negative relationship between haematocrit and brain TK latency (Fig. S4p), all fit into the picture of thiamine deficiency in the European silver eels. Because both haematocrit and the number of *A. crassus* in the swim bladder (Fig. 4k) were related to thiamine deficiency, we analysed whether these two variables were related to each other. There was, however, no such relationship in the present investigation, a finding consistent with most existing literature[222–225], although with some exceptions[226]. It is therefore likely that the variation

in haematocrit and the number of *A. crassus* in the present investigation was, in fact, linked to various degree of thiamine deficiency.

Just like in other organisms, the latency of thiamine-dependent enzymes in non-thiamine-deficient eels should be zero or close to zero. This has been confirmed for the kidney in non-thiamine-deficient European eel, where the mean TK latency was found to be at most 2–6%[221,227]. The majority of the European silver eels in the present investigation had higher liver TK latencies, with a maximum of 15% (Fig. 4f), as well as higher brain TK latencies, with a maximum of 24% (Fig. S4p). These observations add to the evidence of thiamine deficiency in the European silver eels.

Because spawning female silver eels in the Sargasso Sea were not available for analysis[44], the mass balance of SumT in such individuals was modelled for two scenarios, which are available as an xlsx file in the Supplementary Information. By this model we could estimate the degree of thiamine deficiency, as well as plausible values for non-thiamine-deficient silver eels. The model compared the thiamine status at the start of the migration to the Sargasso Sea and just before spawning (release of the eggs). It was kept simple and did not account for loss of thiamine by metabolism and/or excretion. Neither did it account for loss of body mass due to energy metabolism during the migration. Values entered in the model (yellow cells in the xlsx file) were obtained from our own analyses, as well as from the literature.

The model assumed a female that weighed 1 kg at the start of the migration. At this stage, the gonad has begun to grow, but no visible eggs have developed. The mean GTI in the European silver eels was 1.4%, which was used in the model. The total body weight (TBW) change factor was assumed to be 1.30, based on the investigation by Oliveira & Hable[65]. It should be noted that the weight gain is caused mainly by uptake of water, since the silver eels do not eat during their migration to the Sargasso Sea. The GTI just before spawning was assumed to be 50%, based on the investigation by Palstra *et al.*[66]. This means that the gonad grows, not only by water uptake, but also at the expense of the somatic body, which shrinks. In the model, the somatic body was divided into a constant somatic body part (CSBP), consisting of bones, skull, eyes, brain, and scales, which do not shrink during maturation, and the body rest (BR), consisting of skin, gills, swim bladder, stomach, intestine, heart, blood, kidney, spleen, and fat, which shrinks by conversion into eggs. For simplicity, the BR was assumed to shrink uniformly. The CSBP was assumed to constitute 25% of the initial somatic weight, based on the investigation by Balk *et al.*[228]. The mean LSI in the European silver eels was 1.1%, which was used in the model. The white muscle somatic index (WMSI) and red muscle somatic index (RMSI) were assumed to be 41% and 7%, respectively, based on the investigation by Egginton[229].

To obtain information about the sexual maturation, egg production was induced by gonadotropin treatment of female American silver eels caught in Paskamansett River (N1) and St. Lawrence River (O1) and brought to the laboratory. At maturation, their eggs had a mean SumT concentration of 2.7 nmol/g (range 1.7–4.8, n=13), which was used in “Scenario 1 Average today”. The same SumT concentration was assumed for the gonad at the start of the migration. In the European silver eels, *i.e.* at the start of the migration, the mean liver SumT concentration was 11 nmol/g (range 5.7–17, n=38), whereas the mean liver SumT concentration in the mature gonadotropin-treated American silver eels was 6.1 nmol/g (range 4.9–9.6, n=12). Both these

values were used in “Scenario 1 Average today”. In the European silver eels the mean white muscle SumT concentration was 1.8 nmol/g. The mean red muscle SumT concentration was assumed to be 5 times higher, based on a number of observations in European and American eel and other fish species. Red muscle and liver had similar SumT concentrations in actively swimming fish species, such as the Atlantic bluefin tuna (*Thynnus thynnus*) and the Atlantic mackerel (*Scomber scombrus*)[230]. There were also substantial metabolic similarities between red muscle and liver in the American eel[231,232], and the red muscle had ca 5 times higher activity of glucose-6-phosphate dehydrogenase, catalysing the rate limiting reaction in the hexose monophosphate shunt, than the white muscle in the European eel[233]. The SumT concentration in the BR was assumed to be 0.3 times the SumT concentration in the liver. The initial CSBP SumT concentration was set to 1 nmol/g. Balance between available and required SumT in “Scenario 1 Average today” was obtained when the SumT concentrations in CSBP, white muscle, and red muscle were reduced to half from the start of the migration until just before spawning.

For “Scenario 2 Best today”, an indication of what the liver SumT concentration might be in a non-thiamine-deficient European silver eel was obtained by extrapolation to zero latency in Fig. 4f, which gave a value of 15.7±1.9 nmol/g. This was very similar to the liver SumT concentration in the replete group of the American yellow eels, which was 16.2±2.8 nmol/g. Hence, an initial liver SumT concentration of 16 nmol/g was used in “Scenario 2 Best today”. The highest white muscle SumT concentration observed in American silver eels in 2005 was 15.9 nmol/g[234] (Fig. 4i), and the initial white muscle SumT concentration was thus set to 16 nmol/g in “Scenario 2 Best today”. The highest white muscle SumT concentration in the American yellow eels in the swimming endurance experiment was 5.4 nmol/g. It was assumed that the white muscle concentration may decrease to this value during migration and maturation with retained swimming ability. The difference between the mean red and white muscle SumT concentrations in actively swimming fish species may range from a factor of two up to one order of magnitude higher[235]. A factor of two was chosen for the eels in “Scenario 2 Best today” in order not to overestimate the red muscle SumT concentration. This choice was motivated by the metabolic similarities between red muscle and liver described in connection with “Scenario 1 Average today” above, indicating that the SumT concentration should not differ too much between the red muscle and liver. No egg SumT concentrations in non-thiamine-deficient silver eels were available. Hence, the literature was surveyed for such egg SumT concentrations in other fish species with comparable egg sizes and larval development (Table S4). These egg SumT concentrations, which may come from non-thiamine-deficient specimens, were in the range 11–19 nmol/g (Table S4). The SumT concentration in the BR was assumed to be 0.3 times the SumT concentration in the liver. The initial CSBP SumT concentration was set to 2 nmol/g. Balance between available and required SumT in “Scenario 2 Best today” was obtained when the SumT concentration in the immature gonad and the mature eggs was set to 13 nmol/g and the SumT concentrations in CSBP and liver were reduced to half from the start of the migration until just before spawning.

The two modelled scenarios have several important implications. Firstly, the silver eels make a massive investment in their reproduction at the expense of the somatic body. Secondly, the white muscle is the dominant source of thiamine for the eggs, whereas *e.g.* the liver contributes with only a minor part. The same phenomenon has been indicated also in Atlantic salmon[236]. Thirdly, both white muscle and eggs in non-thiamine-deficient individuals should have substantially higher SumT concentrations than they have in the average silver eels today. SumT concentrations of

16 nmol/g in the liver and white muscle and 13 nmol/g in the eggs of non-thiamine-deficient silver eels are indeed plausible. Fourthly, comparison of available SumT in "Scenario 1 Average today" with required SumT in "Scenario 2 Best today" indicated that ca 80% of the necessary thiamine is missing today, and this value is probably an underestimation, since the model did not account for loss of thiamine by metabolism and/or excretion. Such clearance of thiamine during the migration to the Sargasso Sea was expected to be significant by Fitzsimons *et al*.[234]. The severe thiamine deficiency indicated here may also explain why the leptocephali, produced in the laboratory or in aquaculture by hormonal treatment of silver eels[*e.g.* 237–240], all die a few days after hatch. So far, it has been suspected that the problem is to find suitable food for the leptocephali after they have consumed their yolk sac[218], and some progress has, in fact, been made in this respect[240]. The results presented here, however, suggest that thiamine treatment of the silver eels and leptocephali may be necessary for successful aquaculture production of eels on a regular basis.

It is noteworthy that the maximum white muscle SumT concentration in the replete group of the American yellow eels in the swimming endurance experiment was only 5.4 nmol/g, whereas the maximum muscle TDP concentration in the elvers was 13.9 nmol/g (Fig. S4c), and the maximum white muscle SumT concentration in the silver eels was 15.9 nmol/g (Fig. 4i). Also in the yellow eels analysed by Fitzsimons *et al*.[234], the maximum white muscle SumT concentration, 4.1 nmol/g, was similar to that of the replete group of the American yellow eels. As an explanation, we propose that the muscles are more loaded with thiamine in the migratory life stages (glass eels, elvers, and silver eels) than in the more sedentary life stages (yellow eels). It has, in fact, been demonstrated that the transformation of yellow eels into silver eels is associated with altered properties of the muscles, adapting them to a more continuous swimming activity[241].

Impaired spawning migration due to thiamine deficiency has been demonstrated in rainbow trout[242] and coho salmon (*O. kisutch*)[243]. Hence, it is likely that spawning migration is impaired also in the European and American silver eels, which migrate a much longer distance (1,000–6,000 km). This hypothesis is supported by the fact that swimming endurance was reduced at the white muscle SumT concentrations observed in many feral European and American eels today (Figs. 4i,j). It has also been demonstrated that infestation by *A. crassus* has a negative effect on swimming endurance[225]. Our finding, however, that the number of this parasite is related to thiamine deficiency, probably as a secondary effect of immunosuppression, opens the further possibility that both the parasite infestation and the negative effect on swimming endurance are caused by thiamine deficiency. Moreover, a number of investigations have pointed out the importance of a sufficient lipid content in the muscle for the silver eels' migration to their spawning grounds[244–247]. It is thus particularly interesting that the muscle lipid content of European yellow eels has decreased in close parallel with the recruitment of European glass eels in 21 European rivers since around 1980[247]. Belpaire *et al*.[247] showed that the muscle lipid content may, in fact, have decreased to a level that is insufficient for the migration, gonad development, and spawning. It is well known that thiamine deficiency causes a decrease of the body lipid reserves[*e.g.* 74,248]. To the best of our knowledge, the biochemical mechanism behind this relatively fast lipid depletion is unknown. Hence, the phenomenon may be caused by increased metabolic use of the lipids, decreased lipid synthesis, or both. It may be hypothesized that an inhibited hexose monophosphate shunt[249], owing to low TK activity, results in less NADPH available for synthesis of fatty acids. Another possibility is that an inhibited citric acid cycle stimulates the formation of acetyl-CoA from β-oxidation of fatty acids. It has, in fact, been observed recently that the entire citric acid

cycle, *i.e.* not only KGDH, is altered by thiamine deficiency[250,251]. In recent years, decreased muscle lipid content and/or decreased condition has been observed also in other species of fish, such as eelpout, herring, perch (*Perca fluviatilis*), and sprat (*Sprattus sprattus*)[252–254], mammals, such as seals[254], and birds, such as guillemot (*Uria aalge*), barn swallow (*Hirundo rustica*), and tree swallow (*Tachycineta bicolor*)[252,255,256]. Hence, we suspect that also these observations may be a result of the widespread episodic thiamine deficiency.

Salmonines (Salmoninae)

The relationships between activity and latency of TK and KGDH were analysed in the parental Atlantic salmon at E1 in the County of Uppsala and I9 in the County of Blekinge (Table S5a). The slopes were always negative, and in three cases of five, they were significantly different from zero. The brain KGDH latencies were in the range 0–40% (Fig. 5b), and 21 out of 30 specimens had a brain KGDH latency above 6%. The occurrence of nine specimens with a brain KGDH latency below 6% may be explained by the fact that homeostasis in the brain has priority over homeostasis in other organs[82]. The relationships between activity and latency of TK and KGDH were analysed also in the Atlantic salmon larvae at I9 (Table S5a). The slopes were negative in both cases, with a tendency to being different from zero.

A number of sex differences were observed in the parental Atlantic salmon at E1 and I9 (Table S5b). The higher LSI in the females than in the males at I9 was most probably related to increased hepatic activity in the females in connection with their egg production (*e.g.* vitellogenin production). The higher HSI, haematocrit, and brain SumT in the males than in the females at I9 may indicate higher thiamine status in the males, because they make a smaller investment of thiamine in the reproduction than the females. In this context it was unexpected to find higher white muscle SumT concentrations in the females than in the males at I9. This phenomenon may be related to the fact that females store thiamine in their muscles for transfer to the eggs[*e.g.* 236]. Higher liver TK activity in the males than in the females at I9, as well as higher liver KGDH activity and lower KGDH latency in the males than in the females at E1, may indicate a higher thiamine status in the males than in the females in the same way as for HSI, haematocrit, and brain SumT at I9. It is noteworthy that at E1 the extrapolated liver KGDH activity of 15.8±5.6 nmol/min/mg at zero latency in the females was almost identical to the mean liver KGDH activity of 16.3±2.1 nmol/min/mg in the males (Fig. S5i, Table S5b). It was unexpected to find higher liver TK activity and lower liver TK latency in the females than in the males at E1. Possibly, this was a result of different histories of thiamine deficiency (as discussed above for the common eider) between the sexes, owing to their different investments in the reproduction.

The positive relationship between white muscle SumT and total weight in the parental Atlantic salmon at I9 (Fig. S5k) indicates that the thiamine status affects growth. Also at E1, females with thiamine-deficiency-induced offspring mortality (n=129) had lower mean total weight (P=0.0012) and lower mean SCI (P=0.0068) than females without thiamine-deficiency-induced offspring mortality (n=983), as analysed with the paired Student’s *t*-test for the years 2007–2016 (not shown). In this context, it is interesting that both Atlantic salmon and sea trout caught by angling at Mörrums Kronolaxfiske (I9) have increased in size since the beginning of the 1970s[257]. Possibly, larger individuals with higher thiamine status perform better than smaller individuals when it comes to migration and spawning.

Just like in the European eels, there was a positive relationship between the haematocrit and the liver TK activity in the parental Atlantic salmon at I9 (Fig. S5n). There was also a positive relationship between the haematocrit and the brain SumT (Fig. S5o). Moreover, erythrocyte haemoglobin was negatively related to the liver TK activity (Fig. S5p) and the heart SumT (Fig. S5q). Possibly, increased erythrocyte haemoglobin, due to thiamine deficiency, is a way for an individual to compensate for the loss of erythrocytes observed as decreased haematocrit. It may

also be that decreased KGDH activity in the citric acid cycle results in decreased production of reducing equivalents for the respiratory chain, and that this phenomenon is perceived by the body as oxygen deficit, which is miscompensated for by production of erythrocytes with more haemoglobin.

Thiamine deficiency was evident also in the Atlantic salmon larvae, both in the Baltic Sea stock (E1, I9) and in the Atlantic Ocean stock (K1). Liver TK activity was lower in these groups compared with larvae from thiamine-injected females analysed previously by Amcoff *et al.*[70] (Fig. S5r). There was also a negative relationship between liver TK activity and latency (Fig. S5s). Moreover, the liver TK latencies of 23–44% alone were evidence of severe sublethal thiamine deficiency. Liver KGDH latencies of 2–21% were also observed (not shown). Extrapolation to zero latency in Fig. S5s gave an activity of 4.0±1.2 nmol/min/liver, comparable to that of the larvae from thiamine-injected females (Fig. S5r).

The progression of thiamine deficiency symptoms starts with molecular alterations at the subcellular level, such as decreases in thiamine-dependent enzyme activities and alterations in thiamine metabolism. This kind of alterations are followed by subcellular and cellular accumulation of toxic metabolites, *e.g.* lactate. This, in turn, results in damage on the cellular, tissue, and organ levels, *e.g.* necrosis and haemorrhage. At this stage, macroscopic symptoms may start to develop, such as impaired growth, altered behaviour, memory and learning disorders, infectious diseases due to immunosuppression, and, ultimately, premature death. The knowledge about this progression underlines the importance of observation of the early, sublethal symptoms of thiamine deficiency, which occur at substantially higher thiamine concentrations than those associated with direct mortality. Not until recently, however, have the most sensitive (sublethal) thiamine deficiency biomarkers been investigated in feral animals. The previous focus has been mainly on mortality.

Early mortality syndrome (EMS), M74 (from the Swedish word “miljöfaktor” (environmental factor) and year 1974), swim-up syndrome (SUS), and Cayuga syndrome (from Cayuga Lake, NY, USA) are old terms, coined during the last decades to denote early life stage mortality in salmonine offspring in the Great Lakes area (North America) and the Baltic Sea area. In the mid-1990s, John D. Fitzsimons discovered that the common cause of the mortality was thiamine deficiency[258], and since then it is generally accepted that all four terms describe the same phenomenon. Several follow-up investigations in North America and the Baltic Sea area have confirmed Fitzsimons’ initial findings[259–261]. Much of the focus has been on determination of threshold SumT levels for mortality in the offspring. Mean initial egg concentrations of 0.25–3.9 nmol/g in groups of salmonines have been observed to cause offspring mortality in 8–100% of the females[262–267]. The highest mean initial egg SumT concentration, where some of the offspring died of thiamine deficiency, was 3.9 nmol/g. This concentration was observed in lake trout eggs and resulted in offspring mortality in 8% of the females[265]. In Fig. 5h, 3.9 nmol/g is indicated by a dashed line as the threshold level for mortality. Also other authors have suggested a SumT concentration of ca 4 nmol/g in salmonine eggs as a threshold for thiamine-deficiency-induced mortality[268–271]. To fully understand the problem of thiamine deficiency, however, the focus on mortality is too narrow. Thiamine deficiency is well known to induce a multitude of adverse sublethal effects at SumT concentrations much higher than those associated with direct mortality (*i.e.* egg SumT concentrations below ca 4 nmol/g). In salmonines, recent work has suggested a number of

sublethal effects of thiamine deficiency, such as impaired growth[272], reduced swimming endurance[242,243,273], reduced vison and feeding[272,274], and immunosuppression[62,275]. Accordingly, the use of “healthy” as the opposite of “dead” is misleading and should be abandoned. (Unfortunately, the use of this inadequate pair of opposition prevails, *e.g.* at many Atlantic salmon compensatory breeding facilities in Sweden with the task to support the Atlantic salmon stock in the Baltic Sea and the Atlantic Ocean.) By turning the focus also to sublethal effects, the mortality (EMS, M74, SUS, and Cayuga syndrome) appears rather as the tip of an iceberg. It is therefore of utmost importance to determine the SumT threshold for sublethal thiamine deficiency in salmonine eggs, which we set out to do here, both by measurements in salmonines and by revisiting existing literature within this scientific field. Table S5c contains a compilation of egg SumT concentrations in feral salmonines from the Northern Hemisphere, and Table S5d contains a corresponding compilation for aquaculture salmonines. These tabulated egg SumT concentrations range from 4.1 to 66 nmol/g. Hence, the true threshold for sublethal thiamine deficiency must lie somewhere within this range.

Also in feral birds, large variation in egg yolk T concentrations has been observed. In our previous investigation of European birds[1], the range of egg yolk T concentrations was 0.026–30 nmol/g in the common eider and 10–43 nmol/g in the herring gull. It is highly unlikely that an egg SumT maximum/minimum ratio of more than one order of magnitude, as in the common eider or in salmonines (0.25–66 nmol/g), would reflect only the natural variation expected in a pristine ecosystem.

In both bird and fish eggs, roughly half of the contents is yolk, which contains most of the thiamine, whereas the white contains only little thiamine[19,20]. Reported T concentrations in fish eggs refer to whole eggs, whereas reported T concentrations in bird eggs refer to the yolk. This should be taken into account when comparing T concentrations between bird and fish eggs. The reported T concentration in a fish egg is roughly half of that reported in a bird egg with the same yolk T concentration.

Previous investigations of salmonines have demonstrated a strong correlation between SumT concentrations in newly fertilized eggs, eyed eggs, and larvae post hatch[208,276]. This phenomenon was confirmed in the present investigation (newly fertilized eggs vs. eyed eggs, $r=0.92$, $P=0.00019$, $n=10$; newly fertilized eggs vs. larvae 166–175 d°C post hatch, $r=0.96$, $P<0.0001$, $n=10$; eyed eggs vs. larvae 166–175 d°C post hatch, $r=0.95$, $P<0.0001$, $n=10$; not shown). It has also been observed previously that newly fertilized eggs with a higher SumT concentration lose thiamine at a higher rate during their development than newly fertilized eggs with a lower SumT concentration[208,276–279]. Apparently, there are mechanisms for conservation of thiamine when initial egg SumT concentrations are low. In the present investigation, egg SumT concentrations in the Atlantic salmon at I9 were very low (mean=5.2 nmol/g) and decreased only little during the development.

The phenomenon that somatic tissues have an upper limit for how much thiamine they can hold, as described in the Methods, is particularly striking in animal experiments with thiamine doses that may be orders of magnitude higher than physiological concentrations[280,281]. In Atlantic salmon, experimentally exposed to different doses of T in the food, Fynn-Aikins *et al.*[282] demonstrated saturation of SumT in the liver, muscle, heart, and kidney. For salmonine eggs,

however, there seems to be no obvious upper limit for how much thiamine they can hold (Tables S5c,d), and it appears that they sometimes contain more thiamine than is necessary for the production of completely non-thiamine-deficient offspring. For example, the dietary thiamine requirements of several species of domesticated or laboratory animals are substantially lower than the highest thiamine concentrations observed in salmonine eggs (Tables S5c,d). Moreover, a strong linear relationship (P=0.00044, R^2=0.80) between the T concentrations in the diet and the egg yolk has been demonstrated in domestic chicken, at least in the diet T range 4–20 nmol/g fodder[283]. A consequence of this difference between somatic tissues and eggs is that the threshold for sublethal thiamine deficiency is more easily determined in somatic tissues than in eggs. In the somatic tissues, it is possible to measure activity and latency of thiamine-dependent enzymes, as well as thiamine-deficiency-induced toxic metabolites, none of which are produced in the eggs. There is also information in the degree of phosphorylation of the thiamine in the somatic tissues, whereas the eggs contain mostly T. In order to determine the threshold for sublethal thiamine deficiency in salmonine eggs, it was necessary to study the developing larvae originating from eggs with different T concentrations.

Fig. 5h presents original and literature data on egg SumT concentrations in salmonines from the Northern Hemisphere, including the Pacific Ocean, the Great Lakes in North America, the eastern Atlantic Ocean, and the Baltic Sea. The literature data in Fig. 5h are a selection from Tables S5c,d. Four threshold levels for thiamine-deficiency-induced health effects are indicated by horizontal lines:

- Line (A) at 3.9 nmol/g indicates the threshold for mortality in the larvae, as described above.
- Line (B) at 8.3 nmol/g indicates the threshold for 20% reduced specific growth rate (SGR) in the larvae of lake trout[272].
- Line (C) at 12 nmol/g indicates a threshold for liver TK latency in parental Atlantic salmon females.
  - A negative relationship between the egg SumT and the liver TK latency was found in 25 Atlantic salmon females in two Swedish rivers, E1 and I9 (Fig. 5e). This relationship demonstrates that egg thiamine status is directly related to thiamine status of the female. Extrapolation to zero latency gave an egg SumT concentration of 11.7±2.3 nmol/g. Zero latency does not necessarily mean, however, that the corresponding egg SumT concentration is enough to produce completely non-thiamine-deficient offspring. For example, liver TK latency may not be the most sensitive of all thiamine deficiency biomarkers, or the liver TK activity may be permanently reduced, due to previous episodes of thiamine deficiency. A tendency towards such permanent reduction is seen in Fig. 5e if the E1 and I9 Atlantic salmon are analysed separately. The more thiamine-deficient E1 specimens would give an even lower extrapolated egg SumT concentration if analysed alone. Hence, an egg SumT concentration of 12 nmol/g should be regarded as a minimum threshold for liver TK latency in the parental female.
- Line (D) at 17–19 nmol/g indicates a possible threshold range for sublethal thiamine deficiency.
  - According to the exponential relationship presented by Fitzsimons *et al*.[272] a 2% reduction in SGR of the larvae in lake trout occurs at an egg SumT concentration of 17 nmol/g. Assuming that a 2% reduction in SGR lies within the error margin, 17 nmol/g may be a threshold for reduced growth of the larvae.

- The threshold T concentration in white muscle of Atlantic salmon in Fig. 5g was 17–19 nmol/g. According to the relationship in Fig. 5f, this white muscle T concentration range corresponds to an egg T concentration range of 15–20 nmol/g. Hence, an egg SumT concentration of ca 18 nmol/g may be a threshold for sublethal thiamine deficiency.
- In our domestic chicken experiment, a mean liver TK latency of 40% corresponded to a mean liver SumT concentration of 5 nmol/g (n=15, not shown), whereas the liver SumT threshold for zero liver TK latency was 18 nmol/g (n=7, not shown). These SumT values differ by a factor of 3.6. The mean liver TK latency in the Atlantic salmon larvae at I9 was also 40% (range 35–44%, n=10, Fig. S5s), and they came from eggs with a mean SumT concentration of 5.1 nmol/g (range 3.4–7.3 nmol/g, n=14, Figs. 5e,f). If the same factor of 3.6, as in the domestic chicken liver, is applied to the Atlantic salmon eggs at I9, the resulting threshold for liver TK latency in the larvae becomes ca 18 nmol/g, which may be a threshold for sublethal thiamine deficiency.
- Support for 17–19 nmol/g as a possible threshold range comes also from supposedly non-thiamine-deficient aquaculture salmonines (Table S5d), which had egg SumT concentrations in the range 18–62 nmol/g.

As described above, a SumT concentration of 18 nmol/g in salmonine eggs corresponds to roughly 36 nmol/g in herring gull egg yolk, which seems to be enough for the production of healthy offspring[1]. Maybe, there are biochemical similarities between different classes of animal in the thiamine demand of the growing embryo. Moreover, the investigation by Fitzsimons *et al.*[272] indicated that an egg SumT concentration of 19 nmol/g in lake trout is no overestimation of the threshold for sublethal thiamine deficiency, since this concentration was associated with a 6% reduction in foraging rate in the larvae.

Accepting a SumT concentration of 17–19 nmol/g as the threshold for sublethal thiamine deficiency in fresh salmonine eggs leads to a new perspective, where a multitude of sublethal effects are to be expected below this level, apart from direct mortality. Some of these sublethal effects are described above, but more are to be expected, because we have used only a limited number of biomarkers. If there are other, more sensitive biomarkers than those measured here, even the threshold 17–19 nmol/g may be an underestimation. For example, the thiamine-dependent enzyme pyruvate dehydrogenase has been indicated as more sensitive to thiamine deficiency than TK[284]. Fig. 5h and Table S5c show that there is a high probability of sublethal effects of thiamine deficiency in many salmonines in the Northern Hemisphere. The absence of visible mortality among salmonine offspring should not be taken as evidence of full health. It has, in fact, been demonstrated in lake trout larvae that sublethal effects of thiamine deficiency, such as reduced SGR and reduced foraging rate, occurred in a much larger proportion of the population than direct thiamine-deficiency-induced mortality did[272]. Thiamine deficiency may also result in impaired health at later life stages than the larvae. It is well known that thiamine deficiency may cause long-lasting sublethal effects. Hence, both past and present episodes of thiamine deficiency may contribute to impaired health at a certain life stage. In this context, it is interesting that the rate, at which Atlantic salmon offspring return as adults for spawning, has decreased in many areas in the Northern Hemisphere during the last two decades[285–289]. We suspect thiamine deficiency to be the major cause of this negative development. This explanation has, in fact, already been suggested for Atlantic salmon in Swedish rivers[290]. Additional evidence is provided by Friedland *et al.*[288],

who observed that European Atlantic salmon recruitment appears to be governed by factors that affect the growth of post-smolts during their first summer at sea.

Figs. S5t,u, with data from nine rivers in the Baltic Sea area, demonstrate that the thiamine deficiency in Atlantic salmon occurs episodically in both time and space. The average curve is presented separately in Fig. S5t for clarity. It should be noted, however, that the average curve does not account for much of the variation illustrated in Fig. S5u. The average curve also depends on the different monitoring periods of the rivers. It may be especially noted in Fig. S5u that differences are large, both between rivers a certain year, and between years for a certain river. Maybe this episodic pattern is more pronounced between years than between rivers (Fig. S5u). Considering the above discussion on sublethal effects of thiamine deficiency, the observation of zero frequency of females with thiamine-deficiency-induced mortality in the offspring in some of the rivers some of the years does not mean that the offspring was free of sublethal effects. Corresponding monitoring data from North America are not available, but it is most probable that the thiamine deficiency occurs episodically there too. This is, in fact, indicated by Table S5c, where many egg SumT concentrations differ largely between sampling occasions at certain places. Moreover, 77% of the Chinook salmon (*Oncorhynchus tshawytscha*) females from the Yukon River in Alaska, USA, produced eggs with a mean SumT concentration of <8.0 nmol/g in 2012[291]. Finally, it should be remembered that human management of the salmonine populations obscures the full impact of the thiamine deficiency. For example, drift nets were banned in the Baltic Sea in 2008[292], and thiamine bathing of salmonine eggs and larvae in aquaculture is common in the Great Lakes area (USA and Canada)[209] and has been performed to some extent, without bookkeeping, also in the Baltic Sea area. Both measures are likely to affect the salmonine stocks.

Herring (*Clupea harengus*)

Because the analysed herrings had migrated to their spawning grounds at the correct time of the year and were strong enough to catch the jig of the spinning rods, they probably constituted a non-random sample of the Baltic Sea herring population. Contrary to most other field material included in our investigation, these herrings were essentially non-thiamine-deficient. Still, although the spawning grounds at I8 in the County of Blekinge are traditionally well known for their abundance of spawning herring, relatively intense fishing was necessary to obtain the investigated material. On average ca two hours of active fishing were necessary to catch each herring. This may indicate that the abundance of spawning herring could be lower than previously, or be a consequence of the fact that the herring does not eat (catch the jig) during spawning[293–295].

The thiamine deficiency biomarker levels for each sex is presented in Table S6. Sex differences were found for LSI, liver SumT, liver SumT somatic index (SI), and liver/brain SumT ratio. These differences all pertain to the liver and are related to each other. The higher LSI in the females (1.7±0.3%) than in the males (0.6±0.0%) was most probably related to increased hepatic activity in the females in connection with their egg production (*e.g.* vitellogenin production). It is probable that liver SumT concentration was attenuated in the livers of the females and thus lower than in the males. In this context it was interesting to analyse the liver SumT SI, which demonstrated that the females, in fact, had more liver SumT relative to their somatic weight than the males. The higher liver/brain SumT ratio in the males than in the females, was explained by the sex difference in liver SumT combined with no sex difference in brain SumT. The conclusion is that there was no obvious sex difference in the thiamine status.

SCI and GSI for both sexes (Table S6) agreed with literature data on herring ready to spawn[296]. For liver and brain SumT concentrations, no literature data on non-thiamine-deficient herring were available. Compared with the other fish in this investigation, however, the mean liver SumT concentrations of 13.7±2.8 nmol/g in the females and 21.1±4.0 nmol/g in the males were relatively high and could very well be representative of non-thiamine-deficient individuals. The mean brain SumT concentration of 27.8±1.2 nmol/g (pooled sexes, n=11, not shown) were remarkably high and resulted in a liver/brain SumT ratio below one, which in many other species would indicate thiamine deficiency. For these herrings, however, the other thiamine deficiency biomarkers indicated a relatively high thiamine status, so the low liver/brain SumT ratio in this species may be regarded as exceptional. The mean white muscle SumT concentration of 6.3±0.6 nmol/g (pooled sexes, n=20, not shown) was substantially higher than that of other herrings in the Baltic Sea. For example, Sylvander & Bignert[79] reported mean muscle SumT concentrations of 2.59–3.61 nmol/g in material from 1995–2011. In the I8 material, there were no relationships between the proportion TDP and the T concentration, neither in the liver (Fig. S6a) nor in the brain (Fig. S6b). There was, indeed, a negative relationship between the liver TK activity and latency (Fig. S6c), but the latencies were at most 6%. In the brain there was no relationship between the TK activity and latency (Fig. S6d), and neither in the liver (Fig. S6e), nor in the brain (Fig. S6f), was there any relationship between KGDH activity and latency. Mean TK latencies in the liver and brain and mean KGDH latency in the liver were all lower than 2% (Table S6), whereas the mean KGDH latency in the brain was 6.6±4.3% (pooled sexes, n=9, not shown). This higher value was mainly due to three specimens with latencies of 12–15%, which has no obvious interpretation. The overall pattern was a high thiamine status of the herring, which proves that non-thiamine-

deficient individuals may still be found in the field, although they may not be representative for the whole population.

Further Acknowledgements

We thank J. Källström (County of Blekinge, Sweden) for untiring help, as well as all other staff at the Johannishus Manor, for their prompt logistic support during several sampling campaigns. We also thank the ornithologist L. Carlsson (County of Blekinge) for valuable help in many different ways. A. Granhed (Scanfjord Mollösund AB, Mollösund, Sweden) provided blue mussels from the Swedish west coast. When working in Iceland, we received much appreciated help and logistic support from H. Guðmundsson (Flatey), G. Þ. Hallgrímsson (University of Iceland), as well as H. Helgason, E. Palsdóttir, S. Valdimarsson, and the other staff at the Reykjanes Environmental Research Institute in Sandgerði. We were shown great hospitality by A. V. Ásgeirsson at the Álfheimar Guesthouse in Bakkagerði, Borgarfjörður Eystri. Valuable help was also provided by Magnús Þorsteinsson at Höfn in Hafnarhólmi, Borgarfjörður Eystri. We gratefully acknowledge all technical and practical support provided by the staff at the Fisheries Research Station in Älvkarleby (River Dalälven, Sweden), at Mörrums Kronolaxfiske (River Mörrumsån, Sweden), and at Statkraft's Salmon Aquaculture Facility in Laholm (River Lagan, Sweden). At Mörrums Kronolaxfiske, we especially acknowledge C. Elg, P. Karlsson, H. Persson, H. Olsson, U. Sill, O. Söderdahl, and A. Sörensson for their committed support in all work with Atlantic salmon and sea trout. We would also like to commemorate the interest shown and the discerning initiative taken by P. Assarsson, former head of Mörrums Kronolaxfiske, whose further participation in this project was prevented by his untimely death in December 2011. S. Palm (Swedish University of Agricultural Sciences, SLU) performed genetic analysis of the adult Atlantic salmon from River Mörrumsån. Thanks to him, we were able to exclude some hybrids between Atlantic salmon and sea trout from our material. W. Dekker, A. Fjälling, and H. Wickström (SLU), as well as J. Christiansson (Karlstad University, Sweden), provided valuable help in the work with eels. M. Rinaldo and her family kindly provided raw data from the 100 year survey of common eider nests in the Lygne Archipelago (Sweden). R. Hansson translated articles written in Russian and provided valuable comments on the manuscript. M. Sapounidou is acknowledged for fruitful discussions. Thanks also to J.-O. Persson, member of the Statistical Research Group at the Department of Mathematics at Stockholm University (Sweden) for constructive statistical advice. Our work was made possible by access to core facilities at the Department of Environmental Science and Analytical Chemistry (ACES) at Stockholm University. M. Hanson (ACES) kindly assisted us in the thiamine analyses. Finally, we want to thank M. Breitholtz (ACES), J. W. DePierre (Stockholm University), J. D. Fitzsimons (Department of Fisheries and Oceans, Burlington, ON, Canada), R. Holt (Oregon State University, Corvallis, OR, USA), C. E. Kraft (Cornell University, Ithaca, NY, USA), Kjell Larsson (Linnaeus University, Växjö, Sweden), S. V. Marcquenski (Wisconsin Department of Natural Resources, Madison, WI, USA), and D. E. Tillitt (U.S. Geological Survey, Columbia, MO, USA) for their great interest in our work and their knowledgeable support and advice. Any use of trade, product, or firm names in this article is for descriptive purposes only and does not imply endorsement by the US Government.

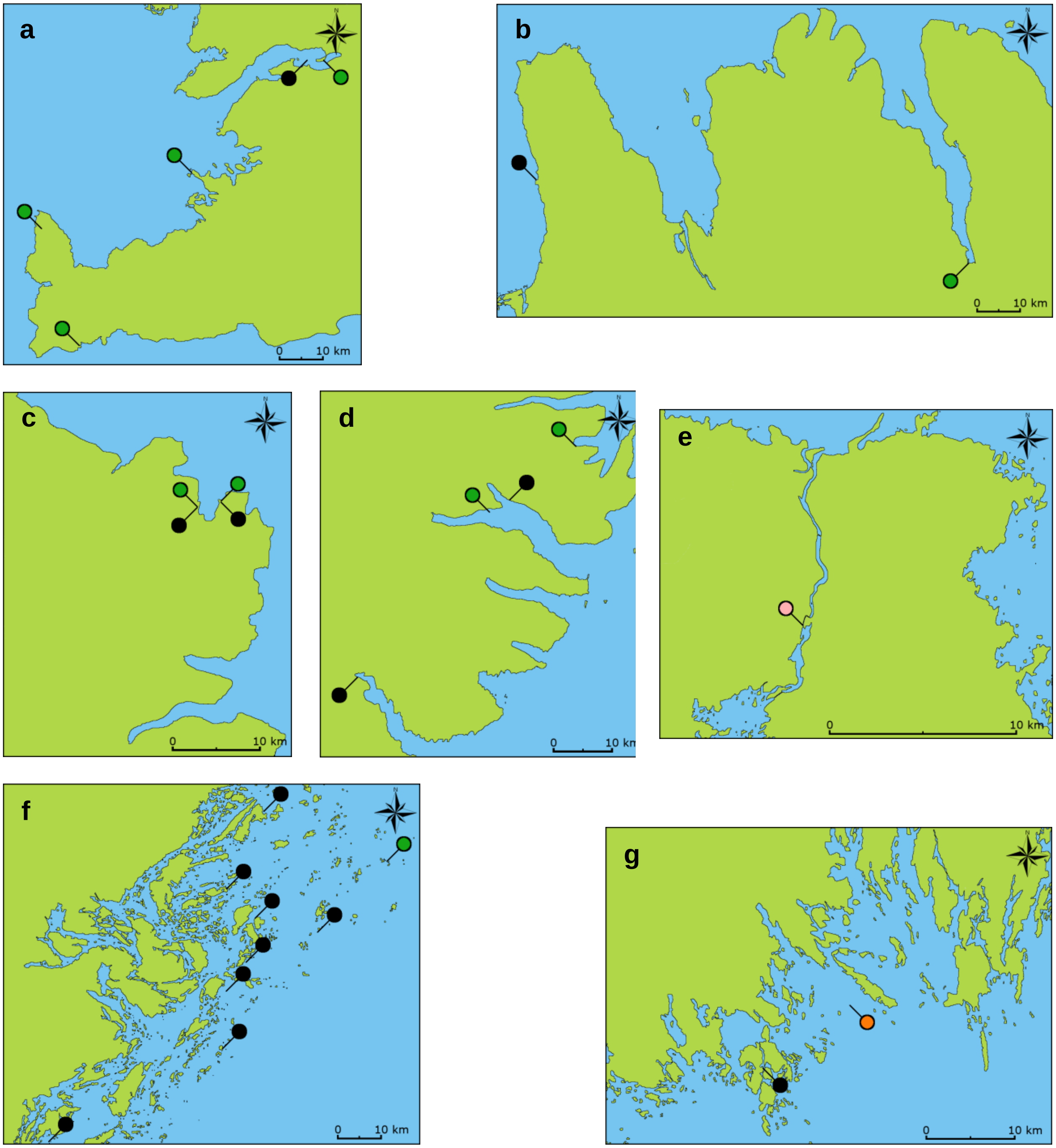

Figure S1. Stations where samples were collected. Black: blue mussel (*Mytilus sp.*). Green: common eider (*Somateria mollissima*). Pink: Atlantic salmon (*Salmo salar*). Orange: chlorophyll *a*. (**a**) South-western Iceland (A). (**b**) Northern Iceland (B). (**c**) North-eastern Iceland (C). (**d**) Eastern Iceland (D). (**e**) County of Uppsala, Sweden (E). (**f**) County of Stockholm, Sweden (F). (**g**) County of Södermanland, Sweden (G). The maps were created with GIMP 2.8.16 (http://www.gimp.org/downloads/).

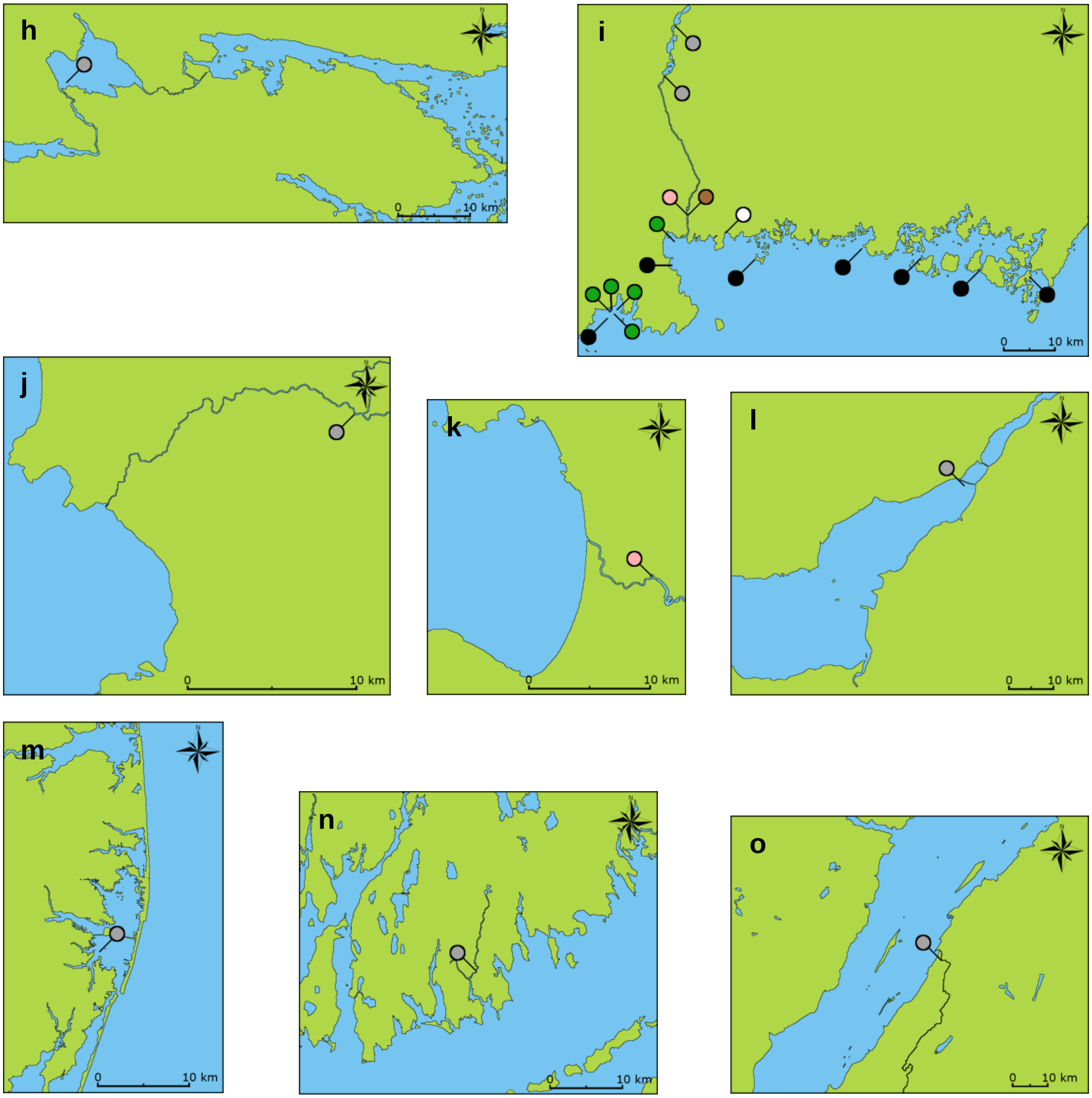

Figure S1 (continued). Stations where samples were collected. Black: blue mussel (*Mytilus sp*.). Green: common eider (*Somateria mollissima*). Grey: European eel (*Anguilla anguilla*) or American eel (*A. rostrata*). Pink: Atlantic salmon (*Salmo salar*). Brown: sea trout (*S. trutta*). White: herring (*Clupea harengus*). (**h**) County of Östergötland, Sweden (H). (**i**) County of Blekinge, Sweden (I). (**j**) County of Skåne, Sweden (J). (**k**) County of Halland, Sweden (K). (**l**) Gloucestershire, UK (L). (**m**) Maryland, USA (M). (**n**) Massachusetts, USA (N). (**o**) Quebec, Canada (O). The maps were created with GIMP 2.8.16 (http://www.gimp.org/downloads/).

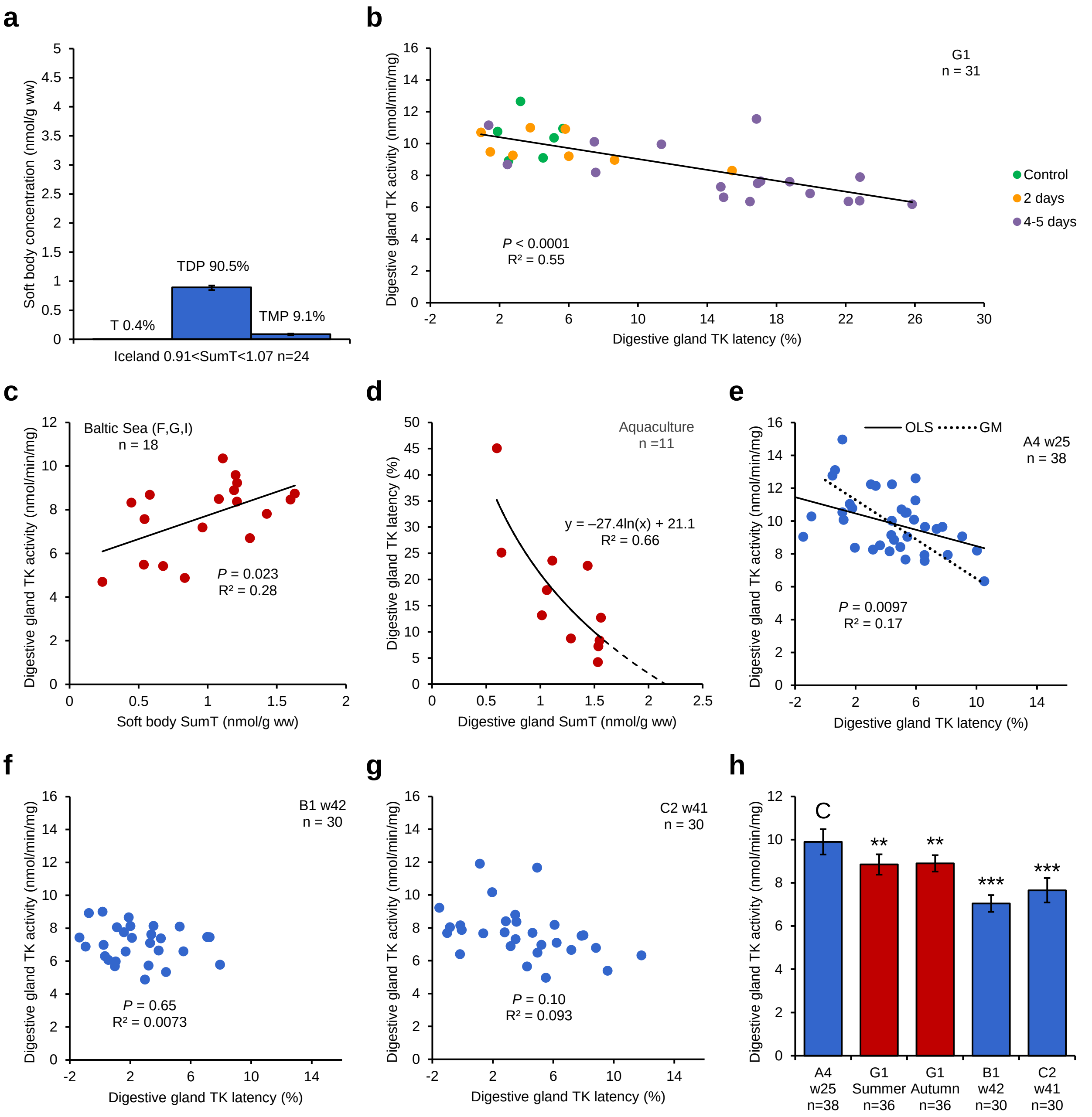

Figure S2. Blue mussel (*Mytilus sp.*). (**a**) Concentration and proportion soft body T, TDP, and TMP in Icelandic specimens with low SumT. (**b**) Digestive gland TK activity and latency in specimens at G1 in the County of Södermanland experimentally subjected to various times of hypoxia and hyperthermia. (**c**) Digestive gland TK activity and soft body (except digestive gland) SumT in the Baltic Sea. (**d**) Digestive gland TK latency and SumT in experimental aquaculture specimens from the Swedish west coast. (**e–g**) Digestive gland TK activity and latency in Icelandic specimens. (**e**) A4 in south-western Iceland. (**f**) B1 in northern Iceland. (**g**) C2 in north-eastern Iceland. (**h**) Digestive gland TK activity in Iceland (blue) and the Baltic Sea area (red). ANOVA-type regression model. ● Bars: arithmetic means. Whiskers: 95% CI. C: control. OLS: ordinary least squares regression. GM: geometric mean regression.

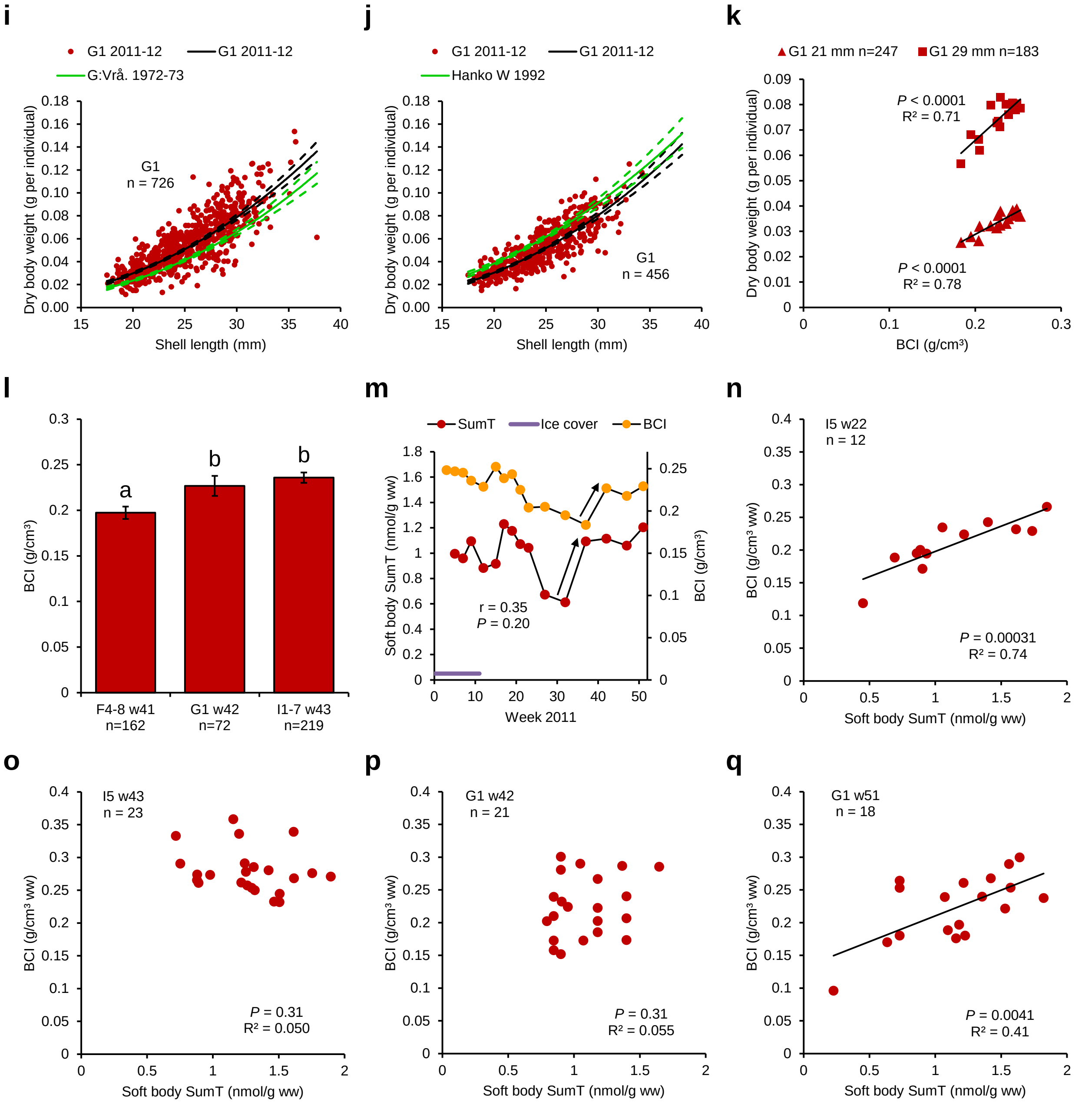

Figure S2 (continued). Blue mussel (*Mytilus sp*.). (i,j) Dry body weight in specimens of different lengths. Dashed lines: 95% CI. (**i**) Outside the reproductive period at G1 2011–2012 (red/black, this investigation) and Vrångskär in the County of Södermanland 1972–1973 (green, adapted from Kautsky[7]). (**j**) March–July at G1 2011–2012 (red/black, this investigation) and west of the Hanko peninsula, Finland, 1992 (green, adapted from Öst & Kilpi[8]). (**k**) Relationship between dry body weight and the wet weight based BCI in two length groups from G1. Each marker represents the arithmetic mean at one sampling occasion. (**l**) BCI in the Baltic Sea area in the autumn 2011. ANOVA. (**m**) Soft body SumT (red) and BCI (orange) at G1 in the County of Södermanland. (**n,o**) BCI and soft body SumT at I5 in the County of Blekinge 2011. (**n**) Week 22. (**o**) Week 43. (**p,q**) BCI and soft body SumT at G1 2011. (**p**) Week 42. (**q**) Week 51. ● Bars: arithmetic means. Whiskers: 95% CI.

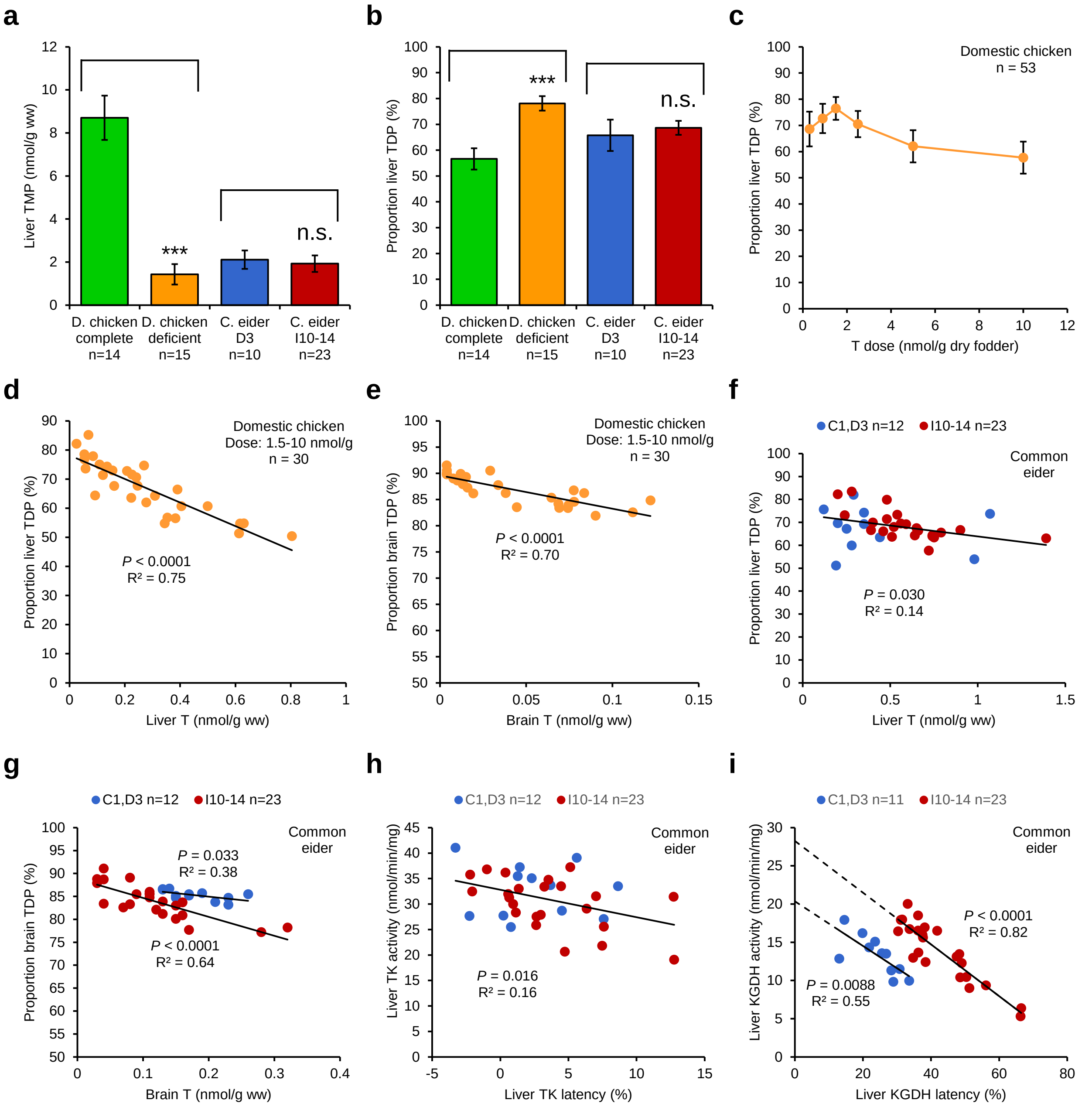

Figure S3. Common eider (*Somateria mollissima*) and domestic chicken (*Gallus gallus*). (a,b) Domestic chicken thiamine "complete" group (green, 17.5 nmol T per g dry fodder) and thiamine "deficient" group (orange, 1.2 nmol T per g dry fodder), common eider females at D3 in eastern Iceland (blue) and at I10–14 in the County of Blekinge (red). Student's *t*-test. (**a**) Liver TMP. (**b**) Proportion liver TDP. (**c**) Domestic chicken response in proportion liver TDP to various doses of T in the dry fodder. (**d,e**) Proportion TDP and T concentration in the liver and brain of domestic chicken given various doses of T in the dry fodder. (**d**) Liver. (**e**) Brain. (**f–i**) Common eider females at C1 and D3 in north-eastern and eastern Iceland (blue) and at I10–14 in the County of Blekinge (red). (**f**) Proportion liver TDP and liver T concentration. (**g**) Proportion brain TDP and brain T concentration. (**h**) Liver TK activity and latency. (**i**) Liver KGDH activity and latency. • Bars: arithmetic means. Whiskers: 95% CI. Group comparisons are indicated by brackets.

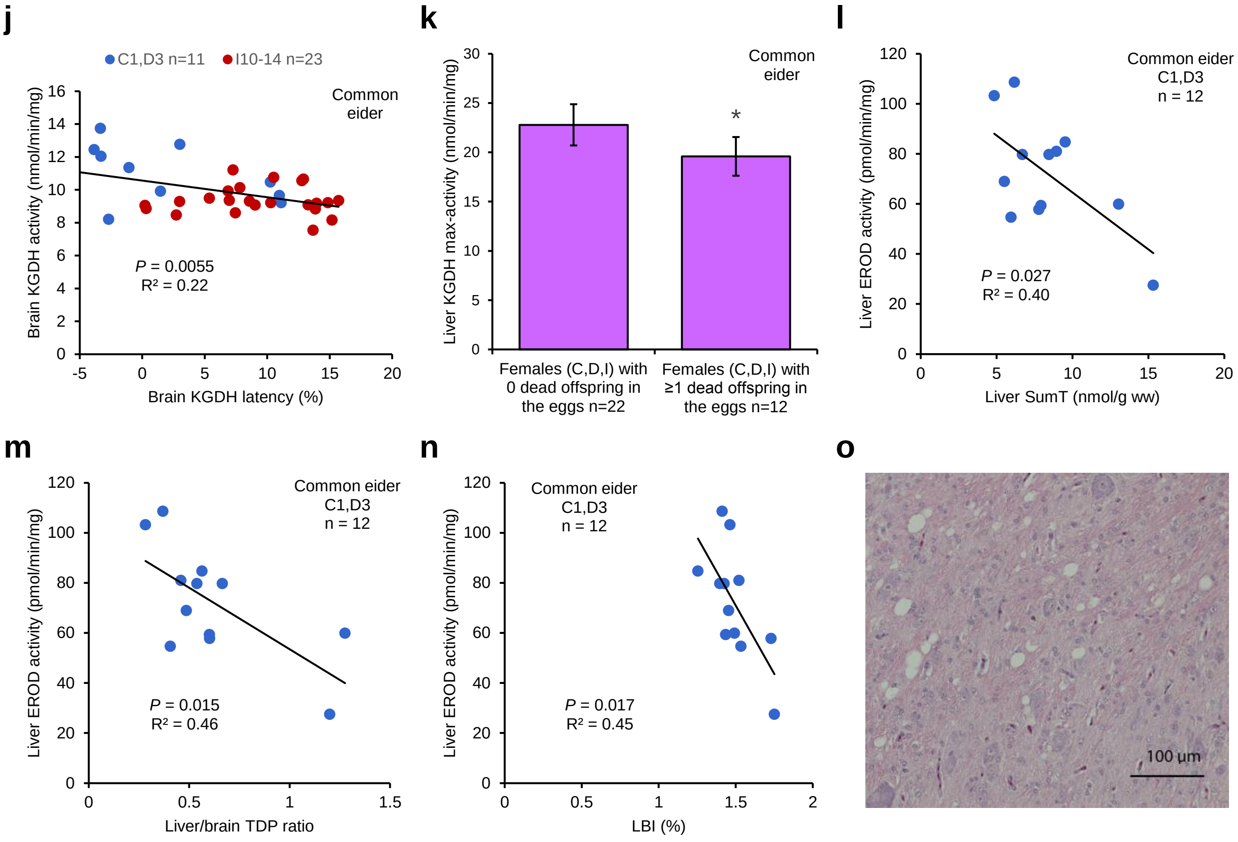

Figure S3 (continued). Common eider (*Somateria mollissima*) and domestic chicken (*Gallus gallus*). (**j**) Brain KGDH activity and latency in common eider females at C1 and D3 in north-eastern and eastern Iceland (blue) and at I10–14 in the County of Blekinge (red). (**k**) Maximum liver KGDH activity in common eider females at C1, D3, and I10–14 with zero dead offspring or at least one dead offspring in the eggs. Student's *t*-test. (**l–n**) Common eider females at C1 and D3. (**l**) Liver EROD activity and liver SumT. (**m**) Liver EROD activity and liver/brain TDP ratio. (**n**) Liver EROD activity and LBI. (**o**) Neuropil vacuolation in the deep cerebellar nuclei of a thiamine-deficient domestic chicken. An unaffected area is seen to the lower right. H&E staining. ● Bars: arithmetic means. Whiskers: 95% CI.

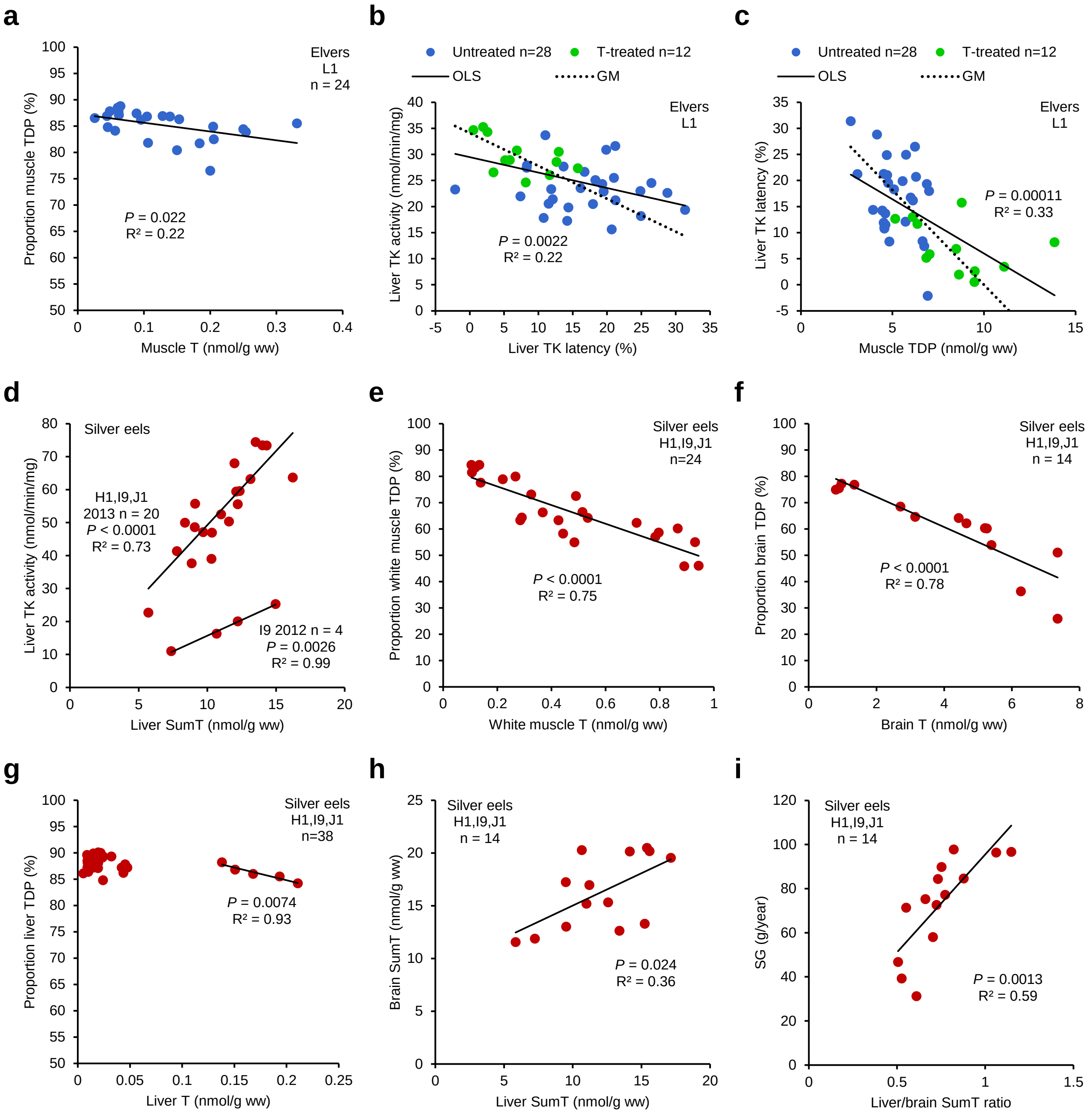

Figure S4. European eel (*Anguilla anguilla*). (**a**–**c**) Untreated (blue) and T-treated (green) elvers from L1 in the UK. (**a**) Proportion muscle TDP and muscle T concentration. (**b**) Liver TK activity and latency. (**c**) Liver TK latency and muscle TDP. (**d**–**i**) Female European silver eels at H1, I9, and J1 in Sweden. (**d**) Liver TK activity and SumT. (**e**) Proportion white muscle TDP and white muscle T concentration. (**f**) Proportion brain TDP and brain T concentration. (**g**) Proportion liver TDP and liver T concentration. (**h**) Brain and liver SumT. (**i**) SG and liver/brain SumT ratio. ● OLS: ordinary least squares regression. GM: geometric mean regression.

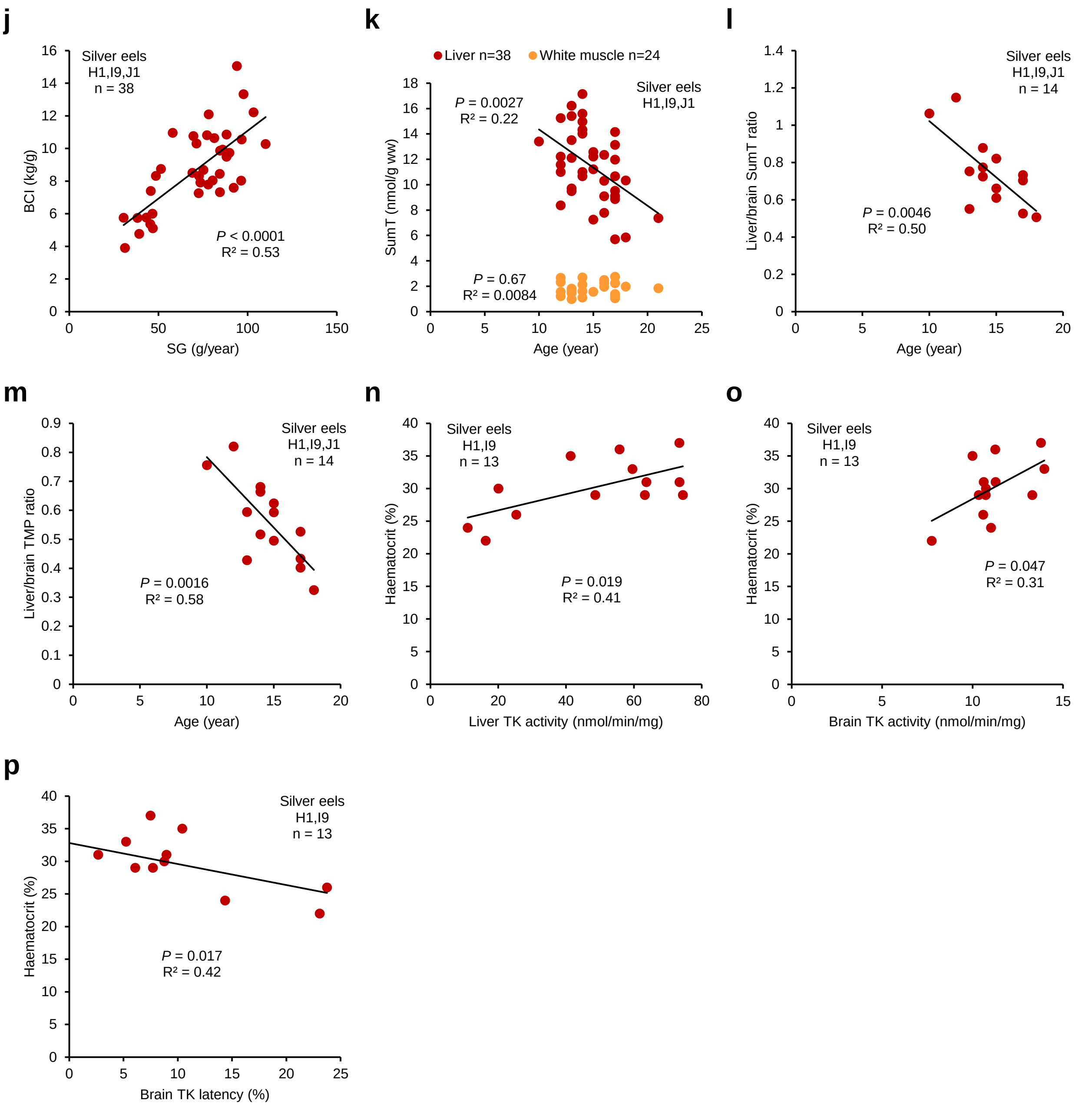

Figure S4 (continued). European eel (*Anguilla anguilla*). (**j–m**) Female European silver eels at H1, I9, and J1 in Sweden. (**j**) BCI and SG. (**k**) Liver (red) and white muscle (orange) SumT and age. (**l**) Liver/brain SumT ratio and age. (**m**) Liver/brain TMP ratio and age. (**n–p**) Female European silver eels at H1 and I9 in Sweden. (**n**) Haematocrit and liver TK activity. (**o**) Haematocrit and brain TK activity. (**p**) Haematocrit and brain TK latency.

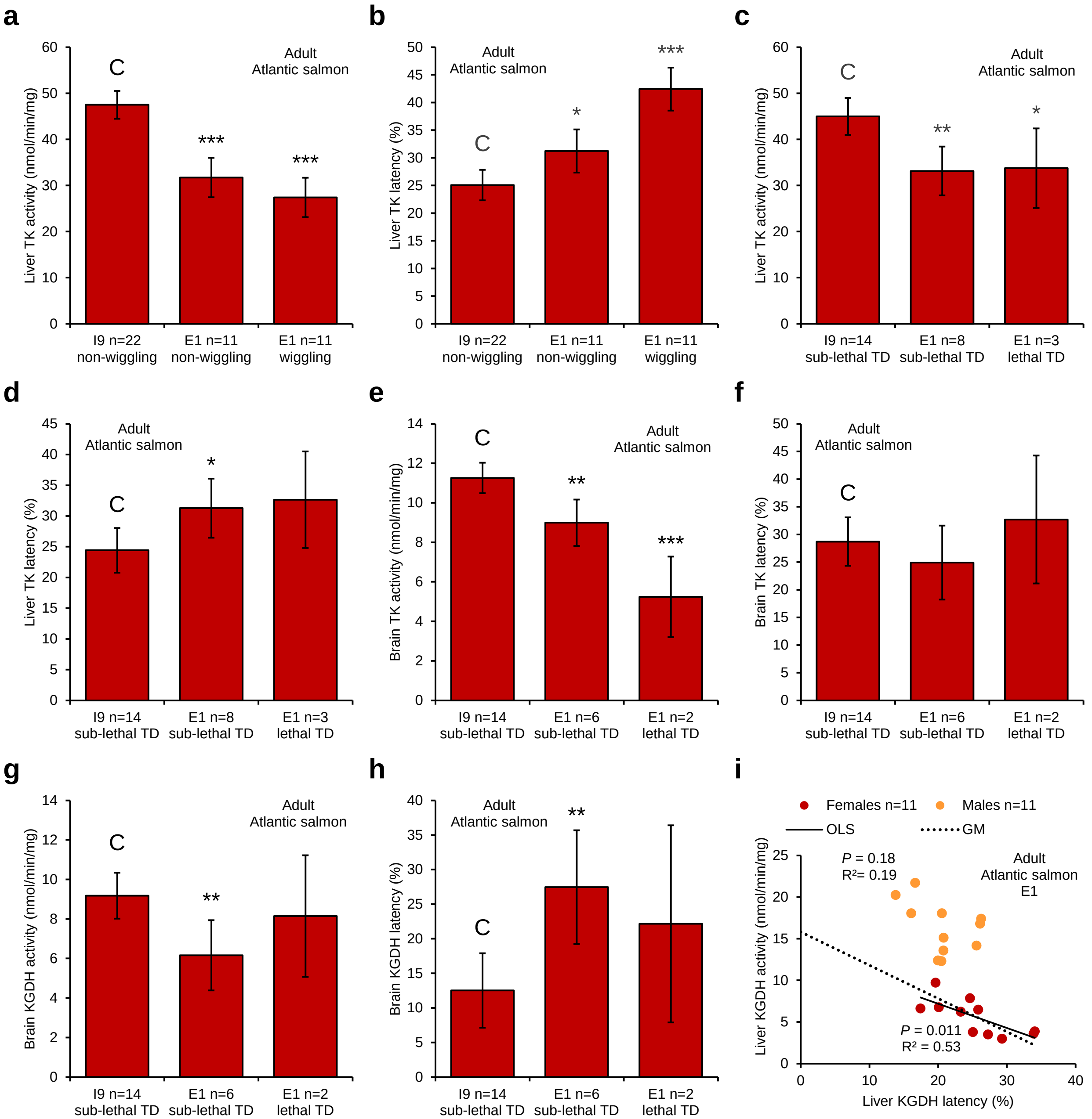

Figure S5. Atlantic salmon (*Salmo salar*). (**a,b**) Non-wiggling and wiggling adults of both sexes at E1 in the County of Uppsala and I9 in the County of Blekinge. ANOVA. (**a**) Liver TK activity. (**b**) Liver TK latency. (**c–h**) Mature females with sublethal and lethal thiamine deficiency (TD) in their offspring at E1 and I9. ANOVA. (**c**) Liver TK activity. (**d**) Liver TK latency. (**e**) Brain TK activity. (**f**) Brain TK latency. (**g**) Brain KGDH activity. (**h**) Brain KGDH latency. (**i**) Liver KGDH activity and latency in adults of both sexes at E1. ● Bars: arithmetic means. Whiskers: 95% CI. C: control. OLS: ordinary least squares regression. GM: geometric mean regression.

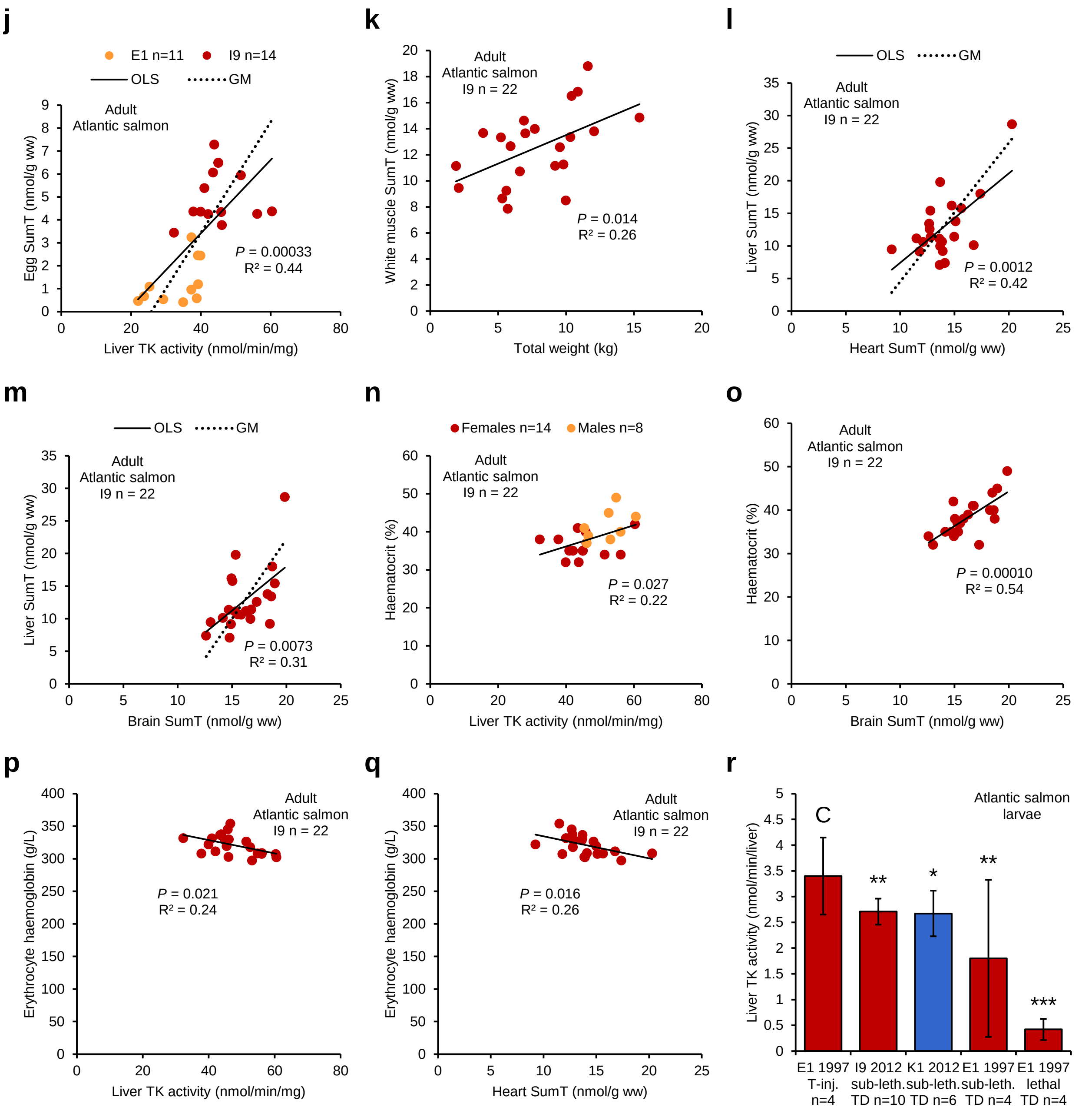

Figure S5 (continued). Atlantic salmon (*Salmo salar*). (**j**) Egg SumT and liver TK activity in mature females at E1 in the County of Uppsala (orange) and I9 in the County of Blekinge (red). (**k–q**) Adults of both sexes at I9. (**k**) White muscle SumT and total weight. (**l**) Liver and heart SumT. (**m**) Liver and brain SumT. (**n**) Haematocrit and liver TK activity. (**o**) Haematocrit and brain SumT. (**p**) Erythrocyte haemoglobin and liver TK activity. (**q**) Erythrocyte haemoglobin and heart SumT. (**r**) Liver TK activity in larvae from n females at E1[70], I9, and K1 166–182 d°C post hatch. Larvae from T-injected females were compared with larvae with sublethal or lethal thiamine deficiency (TD). Z-test. ● Bars: arithmetic means. Whiskers: 95% CI. C: control. OLS: ordinary least squares regression. GM: geometric mean regression.

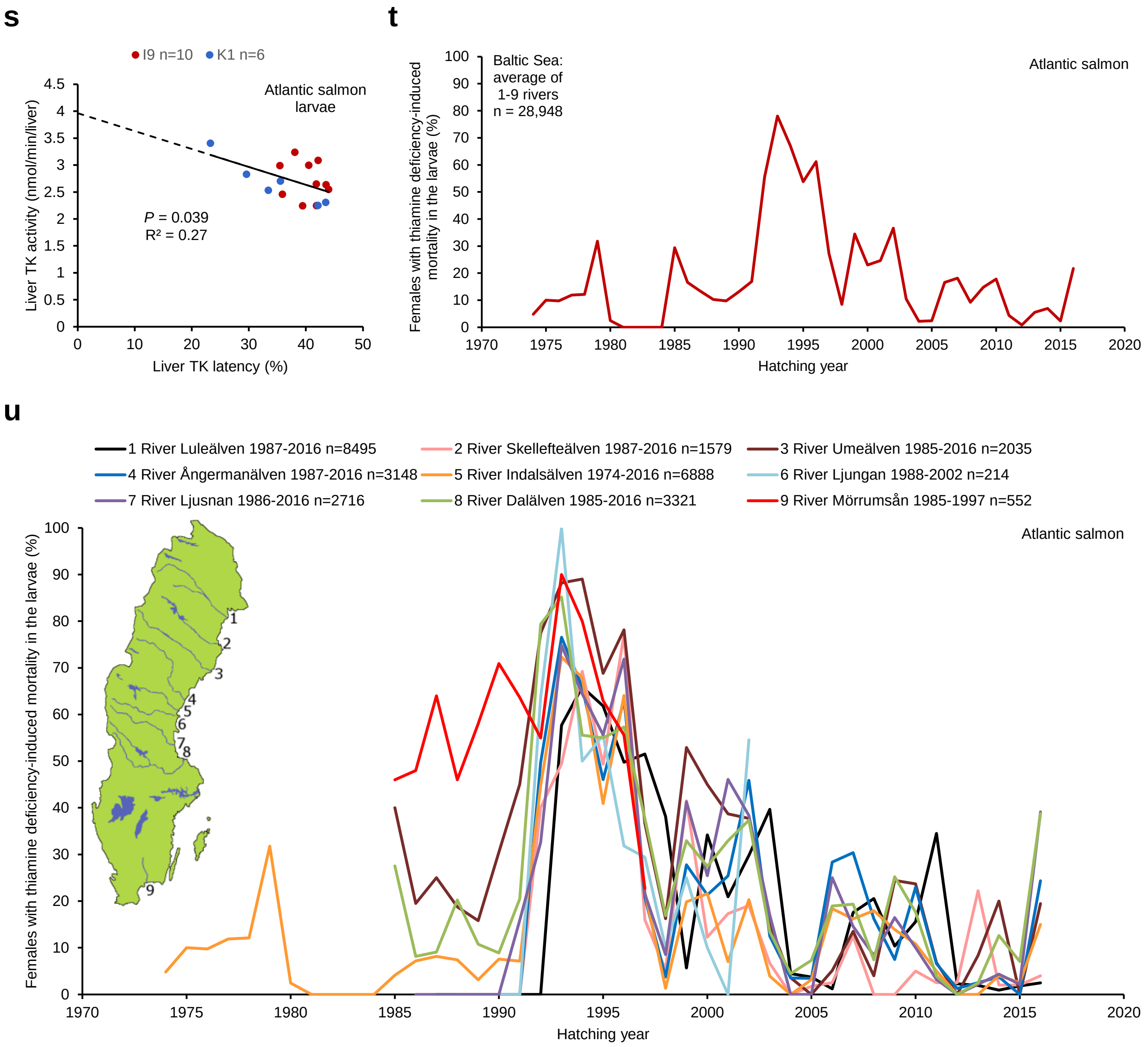

Figure S5 (continued). Atlantic salmon (*Salmo salar*). (**s**) Liver TK activity and latency in larvae from n females at I9 and K1 166–180 d°C post hatch. (**t,u**) Frequency of adult females with thiamine-deficiency-induced mortality in the larvae. (**t**) Baltic Sea average based on one to nine rivers 1974–2016. (**u**) Nine Swedish rivers. The map was created with GIMP 2.8.16 (http://www.gimp.org/downloads/).

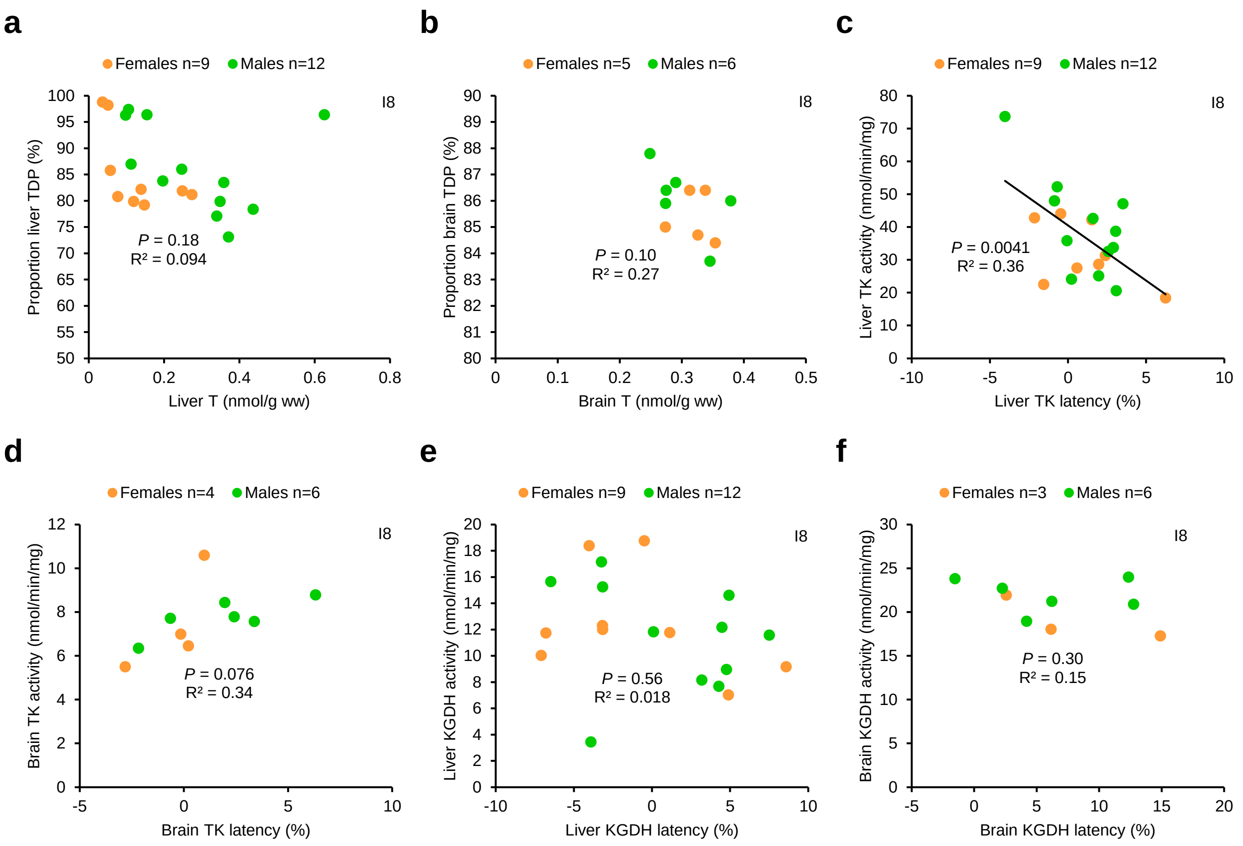

Figure S6. Herring (*Clupea harengus*). Adult females (orange) and males (green) at I8 in the County of Blekinge. Five out of six linear regressions for the pooled sexes were insignificant. (**a**) Proportion liver TDP and liver T concentration. (**b**) Proportion brain TDP and brain T concentration. (**c**) Liver TK activity and latency. (**d**) Brain TK activity and latency. (**e**) Liver KGDH activity and latency. (**f**) Brain KGDH activity and latency.

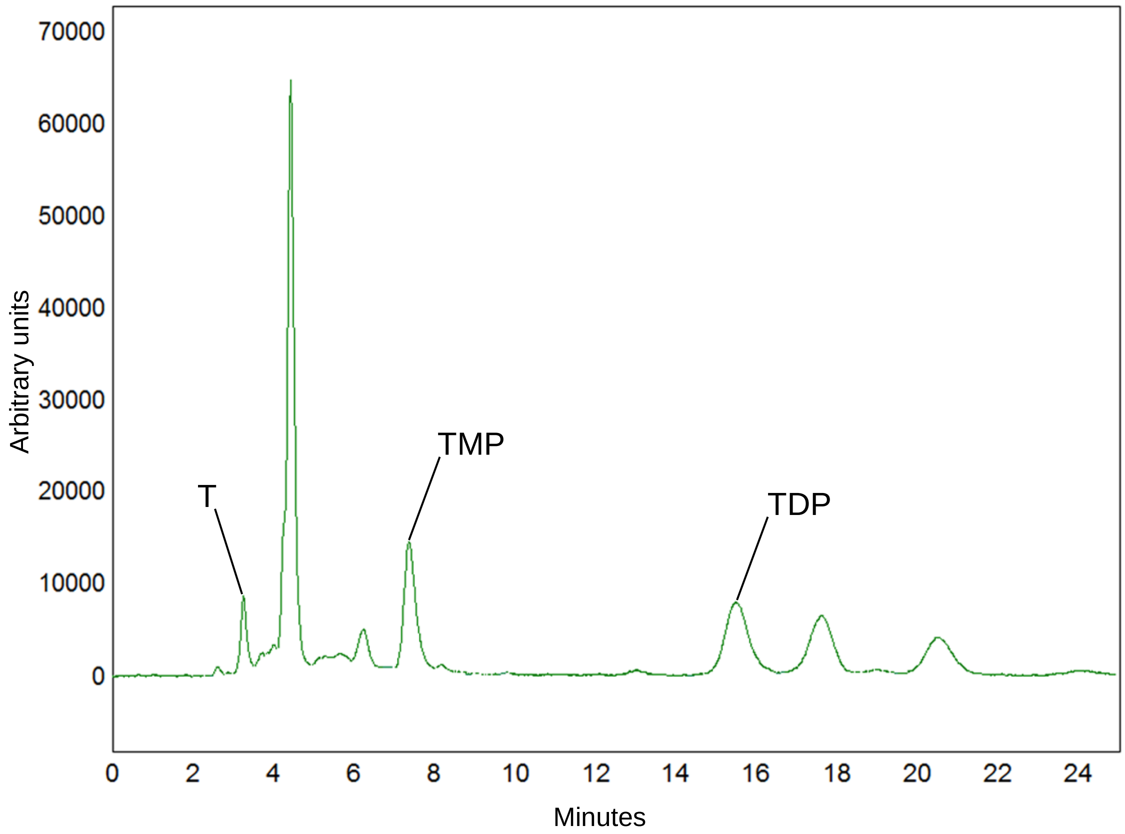

Figure S7a. A typical domestic chicken (*Gallus gallus*) liver chromatogram. Thiamine monophosphate (TMP) and thiamine diphosphate (TDP) concentrations were almost equal, and non-phosphorylated thiamine (T) concentration was comparatively high, although lower than the TMP and TDP concentrations.

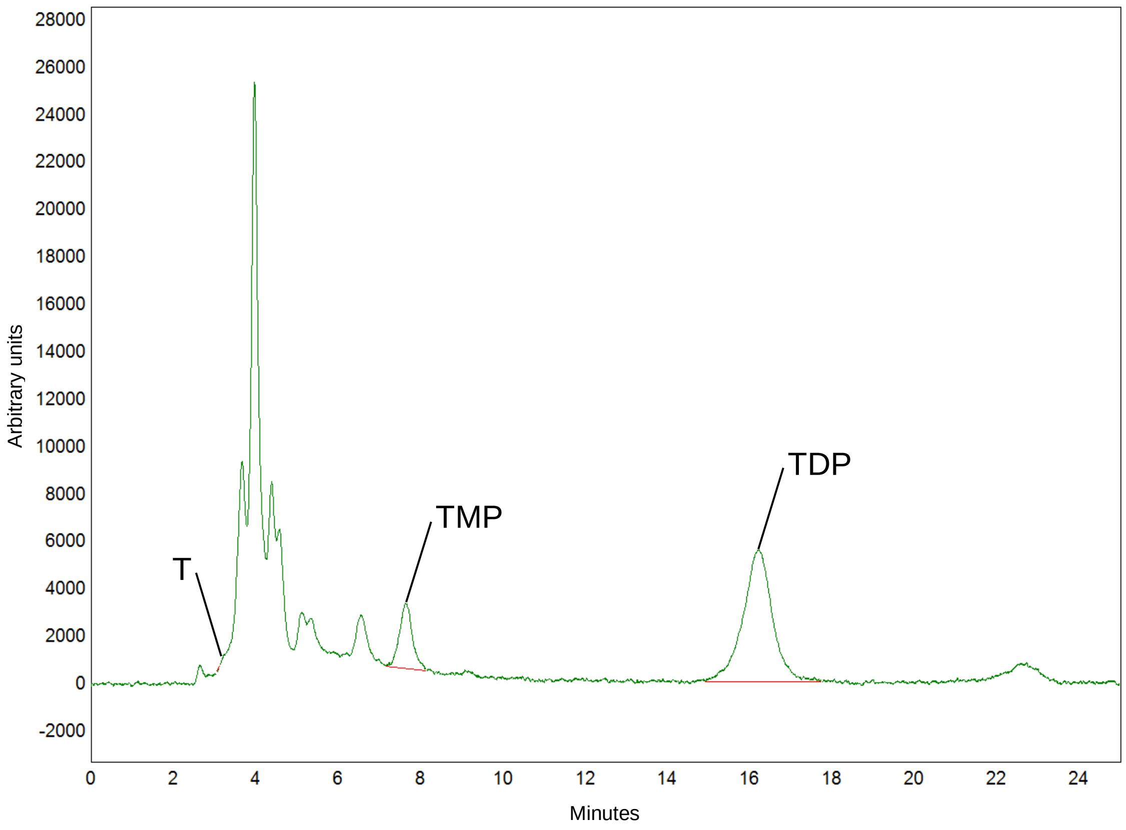

Figure S7b. A typical blue mussel *(Mytilus sp.)* soft body chromatogram. Thiamine monophosphate (TMP) and thiamine diphosphate (TDP) eluted as indicated, whereas non-phosphorylated thiamine (T) was not detectable. Typical, manually drawn, baselines for quantitation are shown in red.

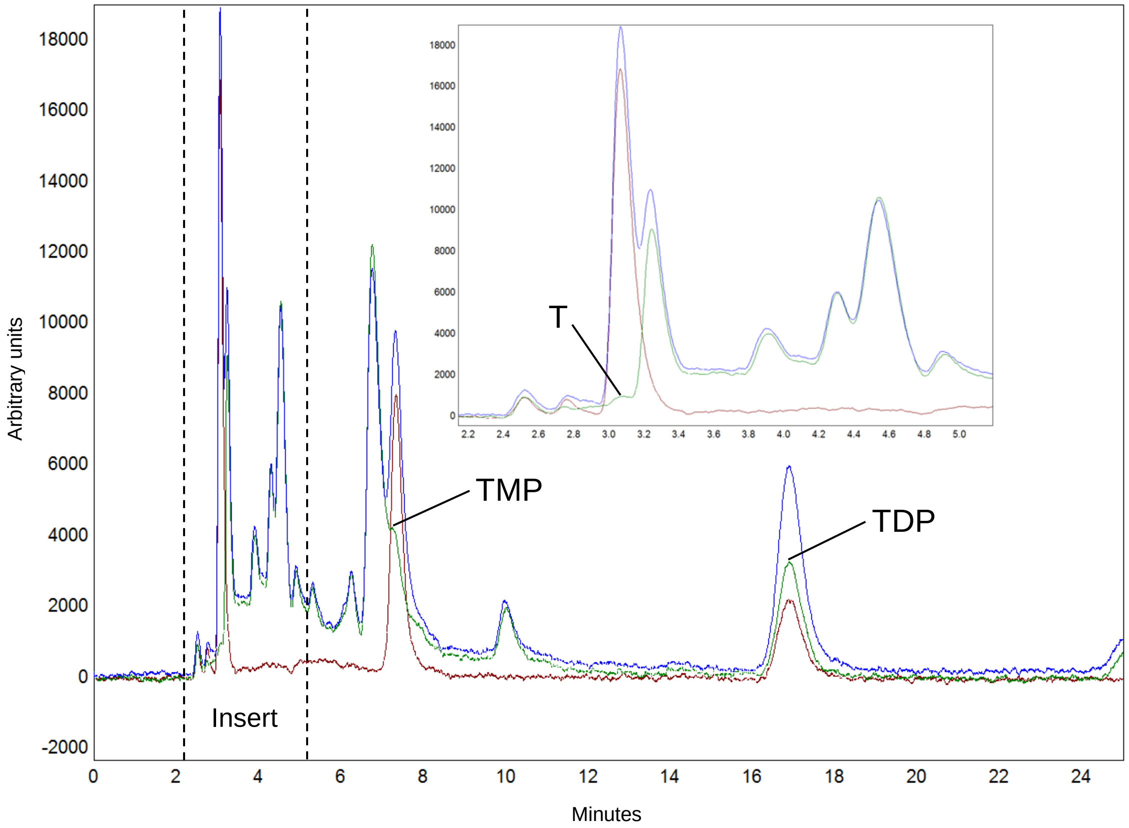

Figure S7c. An atypical blue mussel *(Mytilus sp.)* soft body chromatogram with interfering peaks. Non-phosphorylated thiamine (T), thiamine monophosphate (TMP), and thiamine diphosphate (TDP) peaks (green) were identified by standard addition (blue) and external standard (brown). There was no baseline separation for the T and TMP peaks. The TMP peak appeared only as a shoulder of an interfering peak. Neither T nor TMP concentration was possible to quantitate in this specimen.

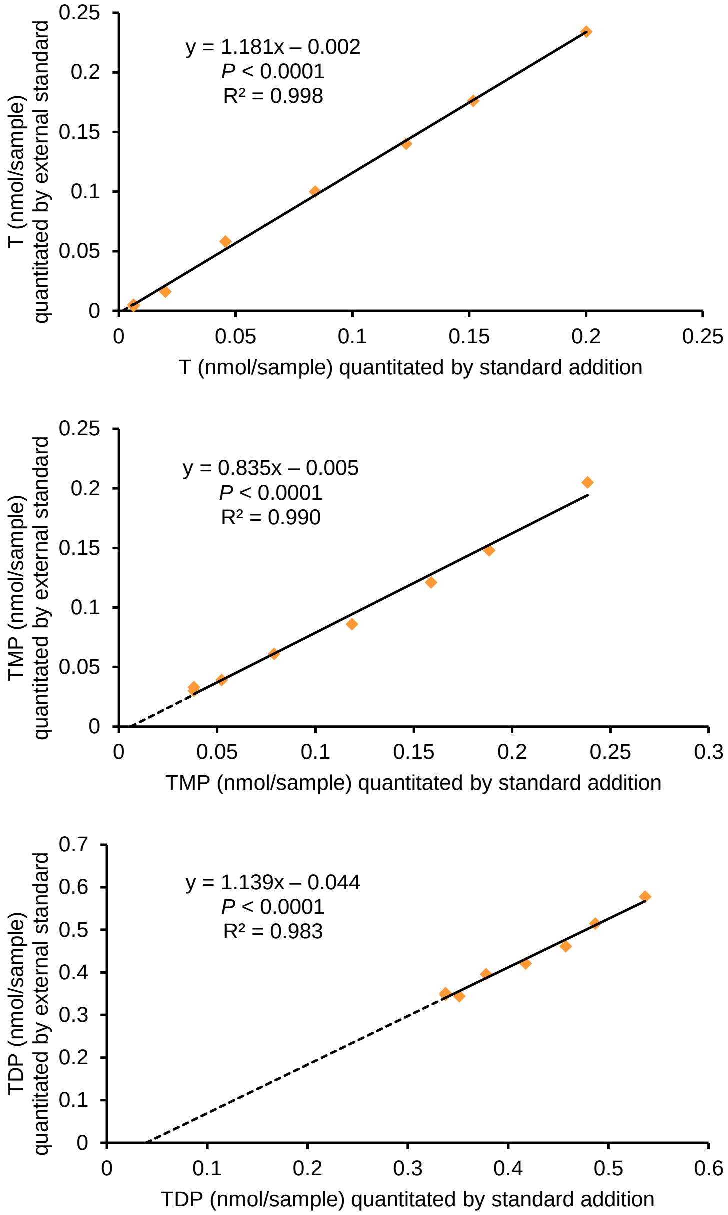

Figure S7d. Comparison of thiamine quantitation by external standard versus standard addition. Non-phosphorylated thiamine (T), thiamine monophosphate (TMP), and thiamine diphosphate (TDP) were quantitated in a homogeneous blue mussel (*Mytilus sp.*) soft body reference material. Quantitation by external standard and standard addition yielded equivalent results, confirmed by statistical testing of the slope and intercept of the regression lines. The slope did not differ from 1 and the intercept did not differ from 0. Accordingly, this quality assurance experiment demonstrated a very good quantitation of thiamine and certified that the thiamine peaks were identified correctly.

Table S1. Collected samples: country, region, code, station, colour in maps, year, type of sample, and coordinates. See also Fig. 1 and Fig. S1.

Country/Region	Code	Station	Code	Colour in maps	Year	Sample	Coordinates (WGS84) Latitude	Longitude
**ICELAND**								
South-western Iceland	A	Grindavík	A1		2005	C. eider egg	N 63° 49.286'	W 22° 30.714'
	A	Miðnesheiði	A2		2005	C. eider egg	N 64° 2.916'	W 22° 40.768'
	A	Miðnesheiði	A2		2006	C. eider egg	N 64° 2.916'	W 22° 40.768'
	A	Kjalarnes	A3		2005	C. eider egg	N 64° 9.460'	W 22° 0.957'
	A	Hvalfjörður	A4		2006	C. eider egg	N 64° 22.411'	W 21° 25.968'
	A	Hvalfjörður	A4		2006	Blue mussel	N 64° 22.409'	W 21° 30.071'
	A	Hvalfjörður	A4		2013	Blue mussel	N 64° 22.409'	W 21° 30.071'
Northern Iceland	B	Húnaflói	B1		2012	Blue mussel	N 65° 50.107'	W 20° 18.880'
	B	Akureyri airport	B2		2006	C. eider egg	N 65° 39.518'	W 18° 4.122'
North-eastern Iceland	C	Borgarfjörður Eystri W	C1		2012	Blue mussel	N 65° 32.113'	W 13° 48.828'
	C	Borgarfjörður Eystri W	C1		2013	Blue mussel	N 65° 32.113'	W 13° 48.828'
	C	Borgarfjörður Eystri W	C1		2013	C. eider egg	N 65° 32.113'	W 13° 48.828'
	C	Borgarfjörður Eystri W	C1		2013	Adult female c. eider	N 65° 32.113'	W 13° 48.828'
	C	Borgarfjörður Eystri NE	C2		2012	Blue mussel	N 65° 32.488'	W 13° 45.059'
	C	Borgarfjörður Eystri NE	C2		2013	Blue mussel	N 65° 32.488'	W 13° 45.059'
	C	Borgarfjörður Eystri NE	C2		2013	C. eider egg	N 65° 32.488'	W 13° 45.059'
Eastern Iceland	D	Norðfjörður	D1		2008	C. eider egg	N 65° 7.872'	W 13° 43.529'
	D	Norðfjörður	D1		2009	C. eider egg	N 65° 7.872'	W 13° 43.529'
	D	Eskifjörður	D2		2013	Blue mussel	N 65° 3.074'	W 13° 57.761'
	D	Reyðarfjörður	D3		2006	C. eider egg	N 65° 2.000'	W 14° 1.828'
	D	Reyðarfjörður	D3		2013	Adult female c. eider	N 65° 2.000'	W 14° 1.828'
	D	Berufjörður	D4		2006	Blue mussel	N 64° 47.221'	W 14° 29.801'
**SWEDEN**								
County of Uppsala	E	River Dalälven	E1		2006	Adult Atl. salmon	N 60° 33.587'	E 17° 26.073'
County of Stockholm	F	Byholmen W	F1		2006	Blue mussel	N 59° 29.220'	E 18° 49.780'
	F	Oxkobb W	F2		2006	Blue mussel	N 59° 25.560'	E 18° 56.820'
	F	Storskäret N	F3		2006	Blue mussel	N 59° 23.820'	E 19° 12.060'
	F	Söderöra	F4		2011	Blue mussel	N 59° 38.773'	E 18° 58.944'
	F	Harö	F5		2011	Blue mussel	N 59° 20.127'	E 18° 54.577'
	F	Södra Skarprunan	F6		2011	Blue mussel	N 59° 16.520'	E 18° 49.756'
	F	Långviksskär	F7		2011	Blue mussel	N 59° 9.298'	E 18° 48.788'
	F	Muskö	F8		2011	Blue mussel	N 58° 57.679'	E 18° 6.374'
	F	Lygne Archipelago	F9		—	C. eider nest survey	N 59° 32.600'	E 19° 29.000'

Table S1 (continued). Collected samples: country, region, code, station, colour in maps, year, type of sample, and coordinates. See also Fig. 1 and Fig. S1.

Country/Region	Code	Station	Code	Colour in maps	Year	Sample	Coordinates (WGS84)	
							Latitude	Longitude
**SWEDEN**								
County of Södermanland	G	Fallsundet	G1		2011	Blue mussel	N 58° 44.615'	E 17° 28.160'
	G	Fallsundet	G1		2012	Blue mussel	N 58° 44.615'	E 17° 28.160'
	G	Station B1	G2		2011	Chlorophyll *a*	N 58° 48.118'	E 17° 37.507'
	G	Station B1	G2		2012	Chlorophyll *a*	N 58° 48.118'	E 17° 37.507'
County of Östergötland	H	River Motala ström	H1		2013	Female Eur. silver eel	N 58° 36.104'	E 15° 53.571'
County of Blekinge	I	Torhamn/Ytterö	I1		2011	Blue mussel	N 56° 5.781'	E 15° 47.143'
	I	Sturkö/Tjurkö	I2		2011	Blue mussel	N 56° 6.458'	E 15° 38.285'
	I	Hasslö bro	I3		2011	Blue mussel	N 56° 7.653'	E 15° 27.553'
	I	Gökalv	I4		2011	Blue mussel	N 56° 8.594'	E 15° 16.903'
	I	Tärnö	I5		2011	Blue mussel	N 56° 7.526'	E 14° 57.498'
	I	Norrören	I6		2011	Blue mussel	N 56° 6.906'	E 14° 42.564'
	I	Södra Gruerna	I7		2011	Blue mussel	N 56° 1.583'	E 14° 30.843'
	I	Karlshamn	I8		2012	Adult herring	N 56° 9.769'	E 14° 51.887'
	I	River Mörrumsån	I9		2011	Sea trout roe	N 56° 11.527'	E 14° 45.011'
	I	River Mörrumsån	I9		2011	Adult Atl. salmon	N 56° 11.527'	E 14° 45.011'
	I	River Mörrumsån	I9		2011	Atl. salmon roe	N 56° 11.527'	E 14° 45.011'
	I	River Mörrumsån	I9		2012	Atl. salmon larvae	N 56° 11.527'	E 14° 45.011'
	I	River Mörrumsån	I9		2012	Female Eur. silver eel	N 56° 30.931'	E 14° 43.007'
	I	River Mörrumsån	I9		2013	Female Eur. silver eel	N 56° 25.978'	E 14° 41.097'
	I	Rödskärven	I10		2011	Adult female c. eider	N 56° 8.857'	E 14° 42.710'
	I	Vållholmen	I11		2011	Adult female c. eider	N 56° 1.928'	E 14° 32.140'
	I	Östra skär	I12		2011	Adult female c. eider	N 56° 1.616'	E 14° 31.673'
	I	Norra skär	I13		2011	Adult female c. eider	N 56° 1.828'	E 14° 31.342'
	I	Stora Gru	I14		2011	Adult female c. eider	N 56° 1.712'	E 14° 31.218'
County of Skåne	J	River Kävlingeån	J1		2013	Female Eur. silver eel	N 55° 46.692'	E 13° 13.884'
County of Halland	K	River Lagan	K1		2012	Atl. salmon larvae	N 56° 30.987'	E 13° 2.989'
**UK**								
Gloucestershire	L	River Severn	L1		2013	Eur. eel elver	N 51° 34.395'	W 2° 41.779'
**USA**								
Maryland	M	Turville Creek	M1		2010	Am. yellow eel	N 38° 22.424'	W 75° 6.979'
Massachusetts	N	Paskamansett River	N1		2011	Female Am. silver eel	N 41° 35.117'	W 70° 59.450'
**CANADA**								
Quebec	O	St. Lawrence River	O1		2011	Female Am. silver eel	N 47° 50.159'	W 69° 32.122'

Table S2a. Comparison of SumT concentration between small, medium, and large blue mussels (*Mytilus sp.*). Different letters (a,b,c) indicate a significant difference (P<0.05, Wald test) in SumT. For significant differences, larger mussels had lower SumT than smaller mussels. The difference in SumT between Small and Large is expressed as percent of Medium SumT.

Group	n	Small 15<x[a]≤23 mm SumT (nmol/g ww) Mean±95% CI	Medium 23<x[a]≤27 mm SumT (nmol/g ww) Mean±95% CI	Large 27<x[a]≤38 mm SumT (nmol/g ww) Mean±95% CI	Small–Large SumT difference (%)
A4,D4 spring 2006	89	3.0±0.2 (a)	3.0±0.5 (a)	2.8±1.1 (a)	6
A4 2013	49	1.7±0.3 (ab)	2.1±0.3 (a)	1.6±0.2 (b)	2
B1,C1,C2,D2 2012–2013	175	1.0±0.1 (a)	0.8±0.1 (b)	0.7±0.1 (c)	45
F4–F8 autumn 2011	84	1.4±0.1 (a)	1.1±0.3 (a)	0.9±1.1 (a)	40
G1 2011–2012	258	1.0±0.1 (a)	1.0±0.1 (a)	0.8±0.1 (b)	16
I1–I6 spring–summer 2011	100	1.4±0.1 (ab)	1.5±0.2 (a)	1.2±0.3 (b)	19
I1–I7 autumn 2011	114	1.4±0.1 (a)	1.2±0.1 (a)	1.2±0.2 (a)	18

[a] x=length.

Table S2b. Ordinary least squares regression of body condition index (BCI) on SumT in blue mussels (*Mytilus sp.*).

**Region**	**Station**	**Code**	**Year**	**Week**	**n**	***P*-value**	**R^2-value**
South-western Iceland	Hvalfjörður	A4	2013	25	28	0.59	0.012
	Hvalfjörður	A4	2013	25	21	0.93	0.00044
Northern Iceland	Húnaflói	B1	2012	42	30	0.032*	0.15
North-eastern Iceland	Borgarfjörður Eystri W	C1	2012	43	24	0.018*	0.23
	Borgarfjörður Eystri W	C1	2013	23	34	0.0050**	0.22
	Borgarfjörður Eystri NE	C2	2012	41	30	0.0082**	0.22
	Borgarfjörður Eystri NE	C2	2013	24	29	0.19	0.062
Eastern Iceland	Eskifjörður	D2	2013	25	28	0.068	0.12
County of Stockholm	Söderöra	F4	2011	41	12	0.75	0.010
	Harö	F5	2011	41	18	0.66	0.012
	Södra Skarprunan	F6	2011	41	18	0.0074**	0.37
	Långviksskär	F7	2011	41	18	0.046*	0.23
	Muskö	F8	2011	41	18	0.21	0.095
County of Södermanland	Fallsundet	G1	2011	5	18	0.11	0.15
	Fallsundet	G1	2011	7	18	0.57	0.020
	Fallsundet	G1	2011	9	20	0.60	0.016
	Fallsundet	G1	2011	12	21	0.63	0.012
	Fallsundet	G1	2011	15	21	0.32	0.052
	Fallsundet	G1	2011	17	15	0.63	0.018
	Fallsundet	G1	2011	19	20	0.88	0.0013
	Fallsundet	G1	2011	21	21	0.84	0.0021
	Fallsundet	G1	2011	23	21	0.33	0.050
	Fallsundet	G1	2011	27	21	0.55	0.019
	Fallsundet	G1	2011	32	18	0.31	0.065
	Fallsundet	G1	2011	37	24	0.019*	0.23
	Fallsundet	G1	2011	42	21	0.31	0.055
	Fallsundet	G1	2011	47	18	0.068	0.19
	Fallsundet	G1	2011	51	18	0.0041**	0.41
	Fallsundet	G1	2012	2	21	0.38	0.041
	Fallsundet	G1	2012	11	21	0.33	0.049
	Fallsundet	G1	2012	24	21	0.40	0.038
County of Blekinge	Torhamn/Ytterö	I1	2011	20	24	0.088	0.13
	Torhamn/Ytterö	I1	2011	43	18	0.039*	0.24
	Sturkö/Tjurkö	I2	2011	20	18	0.46	0.035
	Sturkö/Tjurkö	I2	2011	43	22	0.65	0.011
	Hasslö bro	I3	2011	20	23	0.0025**	0.36
	Hasslö bro	I3	2011	43	22	0.077	0.15
	Gökalv	I4	2011	20	12	0.13	0.21
	Gökalv	I4	2011	43	11	0.59	0.034
	Tärnö	I5	2011	22	12	0.00031***	0.74
	Tärnö	I5	2011	43	23	0.31	0.050
	Norrören	I6	2011	20	11	0.15	0.22
	Norrören	I6	2011	43	11	0.29	0.12
	Södra Gruerna	I7	2011	43	7	0.18	0.32
Total	—	—	—	—	880	<0.0001***	0.35

Table S3a. Comparison of daily SumT intake in experimental domestic chicken (*Gallus gallus*) and feral Baltic Sea common eiders (*Somateria mollissima*). The domestic chickens were given dry fodder corresponding to 10% of their body weight per day, when pair-fed to the lowest dose group, which was offered dry fodder *ad libitum*. The daily SumT intake was calculated for four real SumT doses (25, 10, 1.5, and 0.9 nmol per g dry fodder) and two hypothetical SumT doses (17.5 and 1.2 nmol per g dry fodder). For the common eider we assumed a body weight of 2 kg and a daily food ration of 2 kg[297], consisting entirely of blue mussels (*Mytilus sp.*) from the Baltic Sea. The mean SumT concentration in the soft body of blue mussels from the Baltic Sea was ca 1.2 nmol/g, and since the soft body constituted ca 20% of the total weight (including soft body, shell, and water), the mean SumT concentration in an entire blue mussel was ca 0.24 nmol/g. It appears that the daily SumT intake in the common eider was of the same magnitude as the daily SumT intake in the low-dose experimental domestic chickens, which all showed symptoms of thiamine deficiency on the biochemical level. The thiamine demand may, however, be higher for feral common eider than for experimental domestic chicken because feral birds live under harder conditions of life (temperature, wind, rain, foraging, predator avoidance, *etc.*).

**Species**	**Body weight (bw) (g)**	**Daily food ration (% of bw)**	**Daily food ration (g)**	**Food SumT concentration (nmol/g)**	**Daily SumT intake (nmol)**	**Daily SumT intake (nmol/g bw)**
Domestic chicken	60	10	6	25	150	2.50
	60	10	6	17.5	105	1.75
	60	10	6	10	60	1.00
	60	10	6	1.5	9	0.15
	60	10	6	1.2	7.2	0.12
	60	10	6	0.9	5.4	0.09
Common eider	2,000	100	2,000	0.24	480	0.24

Table S3b. Ordinary least squares regression of liver ethoxyresorufin-*O*-deethylase (EROD) activity on certain thiamine deficiency biomarkers in adult female common eider (*Somateria mollissima*) from Iceland (C1, D3), the County of Blekinge (I10–I14), and the three regions pooled (C, D, I). Both positive and negative linear relationships were found. All statistically significant relationships were consistent, however, with the hypothesis of EROD activity being positively related to thiamine deficiency.

**Thiamine deficiency biomarker (x)**	**Sign of slope (k)**	**Iceland (C1,D3) *P*-value**	**n C1, D3**	**C. of Blekinge (I10–I14) *P*-value**	**n I10–I14**	**Pool (C,D,I) *P*-value**	**n pool (C,D,I)**
BCI (hg/g)	–	0.48	12	0.020*	23	0.014*	35
LBI (%)	–	0.017*	12	0.34	23	0.012*	35
Liver TDP (nmol/g ww)	–	0.011*	12	0.40	23	0.40	35
Liver SumT (nmol/g ww)	–	0.027*	12	0.49	23	0.58	35
Proportion liver TMP (%)	+	0.0022**	12	0.76	23	0.017*	35
Proportion liver TDP (%)	–	0.013*	12	0.95	23	0.11	35
Liver/brain TDP ratio	–	0.015*	12	0.54	23	0.14	35
Liver/brain SumT ratio	–	0.028*	12	0.78	23	0.16	35
Endogenous brain TK activity (nmol/min/mg)	–	0.047*	12	0.86	23	0.97	35
Maximum brain TK activity (nmol/min/mg)	–	0.045*	12	0.89	23	0.82	35

Table S3c. Levels of thiamine deficiency biomarkers linearly related to liver ethoxyresorufin-*O*-deethylase (EROD) activity in the common eider (*Somateria mollissima*) in Eastern Iceland and the County of Blekinge (Sweden). Means of the thiamine deficiency biomarkers presented in Table S3b were compared with Student's *t*-test.

**Thiamine deficiency biomarker**	**Eastern Iceland (D3) Mean±95% CI**	**n D3**	**C. of Blekinge (I10–I14) Mean±95% CI**	**n I10–I14**	**Difference *P*-value**
BCI (hg/g)	2.10±0.17	10	2.13±0.14	23	0.80
LBI (%)	1.46±0.09	10	1.59±0.06	23	0.016*
Liver TDP (nmol/g ww)	4.73±0.84	10	5.27±0.75	23	0.37
Liver SumT (nmol/g ww)	7.18±1.12	10	7.79±1.18	23	0.51
Proportion liver TMP (%)	29.8±5.2	10	23.6±2.6	23	0.017*
Proportion liver TDP (%)	65.7±6.1	10	68.7±2.7	23	0.28
Liver/brain TDP ratio	0.50±0.09	10	0.73±0.09	23	0.0016**
Liver/brain SumT ratio	0.64±0.08	10	0.90±0.11	23	0.0051**
Endogenous brain TK activity (nmol/min/mg)	13.8±1.0	10	12.4±0.7	23	0.026*
Maximum brain TK activity (nmol/min/mg)	13.9±1.1	10	12.6±0.7	23	0.025*
EROD activity (pmol/min/mg)	77.8±13.1	10	58.4±10.0	23	0.025*

Table S4. Biological characteristics and thiamine concentrations of assumed non-thiamine-deficient fish eggs comparable to American eel (*Anguilla rostrata*) eggs.

Species	Egg Ø (mm)	Day-deg[a] from fertilization to hatch (d°C)	Total length at hatch (mm)	Day-deg[a] from fertilization to yolk sac depletion (d°C)	Egg SumT (nmol/g)	Reference
American eel	0.96–1.03	27–36	2.7	>156	—	65
Atlantic cod[b] (*Gadus morhua*)	—	—	—	—	12–19	268
	1.66–1.92	—	—	—	—	298
	1.2–1.6	—	3.6–4.5	—	—	299
	—	84–91	4	157–164	—	300
	—	107	—	>137	—	301
Rainbow smelt (*Osmerus mordax*)	—	140	—	292	>14.9	302
	—	—	—	—	15.6	303
	0.79–0.99	—	—	—	—	304
	1.0	126–141	5.0	198	—	305
	—	134–203	5	—	—	306
	—	149	—	—	—	307
Lake whitefish (*Coregonus clupeaformis*)	—	—	—	—	>11.39	308
	1.88–2.22	—	—	—	—	309
	2.3–2.4	—	—	—	—	310
	—	286–343	10.8–11.3	478–492	—	311
	—	—	11–14	—	—	312

[a] Although day-degrees are convenient to estimate development, the relationship between actual development and day-degrees may vary with the actual temperature.
[b] From the Baltic Sea.

Table S5a. Ordinary least squares regression of activity on latency of thiamine-dependent enzymes in Atlantic salmon (*Salmo salar*) adults and larvae.

**Station**	**Developmental stage**	**Sex**	**Organ**	**Enzyme**	**n**	**Slope**	***P*-value**	**R^2-value**
E1, I9	Adult	Both	Liver	TK	44	Negative	<0.0001***	0.53
E1	Adult	Female	Liver	KGDH	11	Negative	0.011*	0.53
E1	Adult	Male	Liver	KGDH	11	Negative	0.18	0.19
E1, I9	Adult	Both	Brain	TK	30	Negative	0.15	0.074
E1, I9	Adult	Both	Brain	KGDH	30	Negative	<0.0001***	0.43
I9	Larva	Unknown	Liver	TK	10	Negative	0.10	0.30
I9	Larva	Unknown	Liver	KGDH	10	Negative	0.092	0.31

Table S5b. Sex differences in parental Atlantic salmon (*Salmo salar*) at E1 and I9 analysed with Student's *t*-test.

**Variable**	**Station**	**Sex**	**n**	**Mean±95% CI**	***P*-value**
LSI (%)	I9	Female	14	1.20±0.13	0.033*
		Male	8	0.98±0.15	
HSI (%)	I9	Female	14	0.13±0.01	<0.0001***
		Male	8	0.17±0.02	
Haematocrit (%)	I9	Female	14	36±2	0.0026**
		Male	8	42±3	
Brain SumT (nmol/g ww)	I9	Female	14	15.2±0.9	0.0013**
		Male	8	17.8±1.3	
White muscle SumT (nmol/g ww)	I9	Female	14	13.6±1.5	0.016*
		Male	8	10.7±2.1	
Liver TK activity (nmol/min/mg)	I9	Female	14	45.0±4.1	0.029*
		Male	8	51.9±4.5	
Liver TK latency (%)	I9	Female	14	24.4±2.9	0.40
		Male	8	26.2±3.2	
Liver KGDH activity (nmol/min/mg)	E1	Female	11	5.6±1.5	<0.0001***
		Male	11	16.3±2.1	
Liver KGDH latency (%)	E1	Female	11	25.5±3.7	0.028*
		Male	11	20.6±2.8	
Liver TK activity (nmol/min/mg)	E1	Female	11	33.3±4.6	0.0075**
		Male	11	25.8±3.1	
Liver TK latency (%)	E1	Female	11	31.6±5.3	0.0074**
		Male	11	42.0±5.7	

Table S5c. SumT concentrations >4 nmol/g in fresh eggs of feral salmonines (Salmoninae).

SumT[a] (nmol/g)	n[b]	Species[c]	Location	Year	Reference
66	5	Coho salmon	Lake Superior	≤1997	313
44	3	Lake trout	Lake 382, ON, Canada	1994	314
32	5	Lake trout	Roddy Lake, ON, Canada	1994	314
32	6	Lake trout	Roddy Lake, ON, Canada	1996	315
29	6	Lake trout	Lake 442, ON, Canada	1999	315
26	6	Lake trout	Opeongo Lake, ON, Canada	1995	314
26	9	Lake trout	Lake Superior (Isle Royale)	1995	314
24	5	Lake trout	Lake Superior	2002	272
23	8	Lake trout	Lake Superior (Gull Island)	1994	314
22	17	Lake trout	Lake Superior	≤1996	313
22	10	Lake trout	Lake Huron (Thunder Bay)	2009	270
21	6	Lake trout	Lake Manitou, ON, Canada	2003	272
21	24	Lake trout	Lake Huron (Drummond Island)	2009	270
20	—	Lake trout	Lake 468, ON, Canada	≤1998	75
19	18	Lake trout	Lake Manitou, ON, Canada	1994	314
17	6	Lake trout	Lake Manitou, ON, Canada	1994	278
17	6	Lake trout	Lake Manitou, ON, Canada	1995	314
15	21	Lake trout	Lake Michigan (Clay Banks)	2009	270
14	4	Lake trout	Lake Superior	2001	272
13	12	Lake trout	Lake Simcoe, ON, Canada	1994	314
13	10	Rainbow trout	Lake Superior	1995	315
12	31	Chum salmon	Kuskokwim River, AK, USA	2001	291
12	10	Chinook salmon	Kuskokwim River, AK, USA	2001	291
7.9	9	Lake trout	Wellsboro, PA, USA	2002	272
7.8	15	Lake trout	Lake Michigan (Waukegan)	2009	270
7.5	5	Lake trout	Lake Huron (Parry Sound)	2009	270
7.5	8	Chinook salmon	Middle Yukon River, AK, USA	2012	291
7.4	4	Atlantic salmon	River Klarälven, Lake Vänern (Sweden)	1996	268
6.8	20	Coho salmon	Pacific coast	1995	313
6.3	10	Rainbow trout	Lake Michigan	1995	315
6.2	28	Chinook salmon	Upper Yukon River, AK, USA	2012	291
6.1	10	Chinook salmon	Pacific coast	1995	313
5.8	15	Sea trout	River Mörrumsån (Sweden), Baltic Sea	1995	268
5.7	22	Atlantic salmon	River Teno (Finland), Arctic Ocean	1994-6	236
5.2	11	Lake trout	Lake Huron (Tawas Bay)	1994	314
5.0	12	Lake trout	Lake Michigan (Sheboygan Reef)	1994	314
5.0	3	Sea trout	River Emån (Sweden), Baltic Sea	1996	268
4.9	8	Lake trout	Lake Michigan (South Milwaukee)	1994	314
4.8	8	Lake trout	Lake Huron	2002	272
4.7	8	Sea trout	River Gullspång, Lake Vänern (Sweden)	1995	268
4.6	17	Lake trout	Lake Huron	≤1996	313
4.3	12	Coho salmon	Lake Huron	≤1997	313
4.2	10	Coho salmon	Platte River, MI, USA	1997	315
4.1	10	Atlantic salmon	Little Clear Pond, NY, USA	1994	279

[a] SumT analyses are performed on whole eggs. SumT concentrations may pertain to non-water-hardened eggs or water-hardened eggs (ca 25% volume increase).
[b] Number of females.
[c] Scientific names: See the corresponding footnote in Table S5d.

Table S5d. SumT concentrations in fresh eggs of aquaculture salmonines (Salmoninae).

SumT[a] (nmol/g)	n[b]	Species[c]	Location	Year	Reference
62	12	Lake trout	NARL, Wellsboro, PA, USA	1995	316
36	6	Lake trout	NARL, Wellsboro, PA, USA	≤1997	313
30	15	Coho salmon	PRSFH, Beulah, MI, USA	1994	317
28	10	Atlantic salmon	TLAS, Cortland, NY, USA	≤1998	282
22	10	Rainbow trout	Osceola Hatchery, WI, USA	1995	315
21	—	Rainbow trout	Aquaculture, Japan	≤1987	277
21	4	Atlantic salmon	Little Clear Hatchery, NY, USA	1994	279
19	8	Atlantic salmon	TLAS, Cortland, NY, USA	≤1998	282
18	8	Chinook salmon	RFCS, Stouffville, ON, Canada	≤1997	313

[a] SumT analyses are performed on whole eggs. SumT concentrations may pertain to non-water-hardened eggs or water-hardened eggs (ca 25% volume increase).
[b] Number of females.
[c] Scientific names: Atlantic salmon (*Salmo salar*), Chinook salmon (*Oncorhynchus tshawytscha*), chum salmon (*Oncorhynchus keta*), coho salmon (*Oncorhynchus kisutch*), lake trout (*Salvelinus namaycush*), rainbow trout (*Oncorhynchus mykiss*), sea trout (*Salmo trutta*).

Table S6. Levels of thiamine deficiency biomarkers in each sex of Baltic Sea herring (*Clupea harengus*). Sex differences were analysed with Student's *t*-test.

Variable	Sex	n	Range	Mean±95% CI	*P*-value
Total weight (g)	Female	9	48.3–118.8	84.7±14.9	0.62
	Male	12	64.6–100.8	81.1±8.4	
Somatic weight (g)	Female	9	46.2–86.9	68.6±9.5	0.99
	Male	12	54.6–89.7	68.5±7.1	
Total length (cm)	Female	9	19.8–25.3	23.2±1.3	0.87
	Male	12	20.8–25.2	23.1±0.9	
SCI (usually given without unit[a])	Female	9	0.51–0.60	0.54±0.02	0.52
	Male	12	0.50–0.61	0.55±0.02	
GSI (%)	Female	9	4.6–36.7	22.5±6.7	0.20
	Male	12	12.4–23.8	18.4±2.0	
LSI (%)	Female	9	1.1–2.3	1.7±0.3	<0.0001***
	Male	12	0.5–0.7	0.6±0.0	
Blood glucose (mM)	Female	9	6.1–7.8	6.9±0.5	0.81
	Male	12	6.0–8.0	7.0±0.5	
Blood haemoglobin (g/L)	Female	9	92–132	117±10	0.67
	Male	12	102–142	120±8	
Haematocrit (%)	Female	8	33–51	45±6	0.87
	Male	10	38–51	45±3	
Erythrocyte haemoglobin (g/L)	Female	8	244–281	263±13	0.32
	Male	10	233–309	272±16	
White muscle SumT (nmol/g ww)	Female	9	3.8–9.2	6.0±1.2	0.33
	Male	11	5.5–8.1	6.6±0.6	
Liver SumT (nmol/g ww)	Female	9	8.1–20.1	13.7±2.8	0.0056**
	Male	12	11.5–31.4	21.1±4.0	
Liver SumT SI[b] (nmol/hg ww)	Female	9	11.7–31.4	22.7±5.5	0.0010**
	Male	12	8.1–20.1	13.1±2.6	
Brain SumT (nmol/g ww)	Female	5	26.0–30.0	27.7±1.9	0.88
	Male	6	24.9–30.8	27.9±2.3	
Liver/brain SumT ratio	Female	5	0.29–0.67	0.49±0.19	0.021*
	Male	6	0.55–1.08	0.78±0.18	
Liver TK activity (nmol/min/mg)	Female	9	18.4–48.0	34.0±8.1	0.35
	Male	12	20.6–73.7	39.5±9.3	
Liver TK latency[c] (%)	Female	9	0–6.3	0.9±2.0	0.81
	Male	12	0–3.5	1.1±1.4	
Brain TK activity (nmol/min/mg)	Female	4	5.5–10.6	7.4±3.5	0.70
	Male	6	6.4–8.8	7.8±0.9	
Brain TK latency[c] (%)	Female	4	0–1.0	0±2.2	0.20
	Male	6	0–6.3	1.9±3.2	
Liver KGDH activity (nmol/min/mg)	Female	9	7.0–18.8	12.4±3.0	0.49
	Male	12	3.4–17.2	11.1±2.7	
Liver KGDH latency[c] (%)	Female	9	0–8.6	0±2.9	0.61
	Male	12	0–7.5	0.1±3.6	
Brain KGDH activity (nmol/min/mg)	Female	3	17.3–22.0	19.1±6.2	0.099
	Male	6	19.0–24.0	21.9±2.0	
Brain KGDH latency[c] (%)	Female	3	2.6–14.9	7.9±15.8	0.67
	Male	6	0–12.7	6.0±5.9	

[a] The formal unit is g per cm^3

[b] Liver SumT somatic index = nmol liver SumT per hg somatic weight

[c] Negative latencies truncated

Supplementary References

1. Balk, L. *et al*. Wild birds of declining European species are dying from a thiamine deficiency syndrome. *Proc. Natl. Acad. Sci. USA* **106,** 12001–12006 (2009).
2. Väinölä, R. & Hvilsom, M. M. Genetic divergence and a hybrid zone between Baltic and North Sea *Mytilus* populations (Mytilidae: Mollusca). *Biol. J. Linn. Soc.* **43,** 127–148 (1991).
3. Seed, R. Systematics evolution and distribution of mussels belonging to the genus *Mytilus*: An overview. *Am. Malacol. Bull.* **9,** 123–137 (1992).
4. Koehn, R. K., Hall, J. G., Innes, D. J. & Zera, A. J. Genetic differentiation of *Mytilus edulis* in eastern North America. *Mar. Biol.* **79,** 117–126 (1984).
5. Väinölä, R. & Strelkov, P. *Mytilus trossulus* in Northern Europe. *Mar. Biol.* **158,** 817–833 (2011).
6. Kuchel, R. P., Nair, S. & Raftos, D. A. Changes in the transcriptional expression of oxidative stress response genes in Akoya pearl oysters (*Pinctada fucata*) exposed to air and mechanical agitation. *Aquaculture* **362–363,** 33–38 (2012).
7. Kautsky, N. Quantitative studies on gonad cycle, fecundity, reproductive output and recruitment in a Baltic *Mytilus edulis* population. *Mar. Biol.* **68,** 143–160 (1982).
8. Öst, M. & Kilpi, M. Blue mussels *Mytilus edulis* in the Baltic: Good news for foraging eiders *Somateria mollissima*. *Wildlife Biol.* **4,** 81–89 (1998).
9. Jeffrey, S. W. & Humphrey, G. F. New spectrophotometric equations for determining chlorophylls a, b, c_1, and c_2 in higher plants, algae and natural phytoplankton. *Biochem. Physiol. Pflanz.* **167,** 191–194 (1975).
10. Madsen, F. J. On the food habits of diving ducks in Denmark. *Dan. Rev. Game Biol.* **2,** 157–266 (1954).
11. Pethon, P. Food and feeding habits of the common eider (*Somateria mollissima*). *Nytt Mag. Zool.* **15,** 97–111 (1967).
12. Player, P. V. Food and feeding habits of the common eider at Seafield, Edinburgh, in winter. *Wildfowl* **22,** 100–106 (1971).
13. Cantin, M., Bédard, J. & Milne, H. The food and feeding of common eiders in the St. Lawrence estuary in summer. *Can. J. Zool.* **52,** 319–334 (1974).
14. Thompson, B. Transmission dynamics of *Profilicollis botulus* (Acanthocephala) from crabs (*Carcinus maenas*) to eider ducks (*Somateria mollissima*) on the Ythan estuary, N.E. Scotland. *J. Anim. Ecol.* **54,** 605–616 (1985).
15. Bustnes, J. O. & Erikstad, K. E. The diets of sympatric wintering populations of common eider *Somateria mollissima* and king eider *S. spectabilis* in northern Norway. *Ornis Fennica* **65,** 163–168 (1988).
16. Bustnes, J. O. & Erikstad, K. E. Size selection of common mussels, *Mytilus edulis*, by common eiders, *Somateria mollissima*: Energy maximization or shell weight minimization? *Can. J. Zool.* **68,** 2280–2283 (1990).

17. DiNicolantonio, J. J., Niazi, A. K., Lavie, C. J., O'Keefe, J. H. & Ventura, H. O. Thiamine supplementation for the treatment of heart failure: A review of the literature. *Congest. Heart Fail.* **19,** 214–222 (2013).

18. Abram, J. & Goodwin, T. L. Factors affecting chicken bleed out – a review. *World Poultry Sci. J.* **33,** 69–75 (1977).

19. Hisil, Y. & Ötles, S. Changes of Vitamin B_1 concentrations during storage of hen eggs. *Lebensm. Wiss. Technol.* **30,** 320–323 (1997).

20. Souci, S. W., Fachmann, W. & Kraut, H. *Food Composition and Nutrition Tables* 171–174 (Medpharm, 2008).

21. Bäckermann, S., Poel, C. & Ternes, W. Thiamin phosphates in egg yolk granules and plasma of regular and embryonated eggs of hens and in five- and seven-day-old embryos. *Poultry Sci.* **87,** 108–115 (2008).

22. Hoyt, D. F. Practical methods of estimating volume and fresh weight of bird eggs. *Auk* **96,** 73–77 (1979).

23. Swennen, C. & Van der Meer, J. Variation in egg size of common eiders. *Ardea* **80,** 363–373 (1992).

24. Swennen, C. & Van der Meer, J. Composition of eggs of common eiders. *Can. J. Zool.* **73,** 584–588 (1995).

25. Anonymous. *Nutrient Requirements of Poultry, Ninth Revised Edition, 1994* (National Academy Press, 1994).

26. Schmidt, J. The breeding places of the eel. *Phil. Trans. R. Soc. Lon. B* **211,** 179–208 (1923).

27. McCleave, J. D., Kleckner, R. C. & Castonguay, M. Reproductive sympatry of American and European eels and implication for migration and taxonomy. *Am. Fish. Soc. Symp.* **1,** 286–297 (1987).

28. Béguer-Pon, M., Castonguay, M., Shan, S., Benchetrit, J. & Dodson, J. J. Direct observations of American eels migrating across the continental shelf to the Sargasso Sea. *Nat. Commun.* **6,** 8705; 10.1038/ncomms9705 (2015).

29. Helfman, G. S., Bozeman, E. L. & Brothers, E. B. Size, age, and sex of American eels in a Georgia River. *Trans. Am. Fish. Soc.* **113,** 132–141 (1984).

30. Kleckner, R. C. & McCleave, J. D. Spatial and temporal distribution of American eel larvae in relation to North Atlantic Ocean current systems. *Dana* **4,** 67-92 (1985).

31. Guérault, D. *et al*. Glass eel arrivals in the Vilaine estuary (Northern Bay of Biscay) in 1990: Demographic features and early life history. *Irish Fish. Invest. Ser. A Freshwater* **36,** 5–14 (1992).

32. Suzuki, Y., Tsutui, S., Nishikawa, J., Nishida, S. & Nakamura, O. Presumption of food of eel leptocephalus: A review. *J. Taiwan Fish. Res.* **9,** 73–76 (2001).

33. Jessop, B. M., Shiao, J.-C., Iizuka, Y. & Tzeng, W.-N. Migratory behaviour and habitat use by American eels *Anguilla rostrata* as revealed by otolith microchemistry. *Mar. Ecol. Prog. Ser.* **233,** 217–229 (2002).

34. Tesch, F. W. *The Eel* 119–212 (Blackwell Science, 2003).

35. Jessop, B. M. Migrating American eels in Nova Scotia. *Trans. Am. Fish. Soc.* **116,** 161–170 (1987).

36. Colombo, G. & Grandi, G. Histological study of the development and sex differentiation of the gonad in the European eel. *J. Fish Biol.* **48,** 493–512 (1996).

37. Degani, G. & Gallagher, M. L. *Growth and Nutrition of Eels* (Laser Pages Publishing Ltd., 1995).

38. Oliveira, K. Life history characteristics and strategies of the American eel, *Anguilla rostrata*. *Can. J. Fish. Aquat. Sci.* **56,** 795–802 (1999).

39. van Ginneken, V. *et al*. Eel migration to the Sargasso: Remarkably high swimming efficiency and low energy costs. *J. Exp. Biol.* **208,** 1329–1335 (2005).

40. Fricke, H. & Kaese, R. Tracking of artificially matured eels (*Anguilla anguilla*) in the Sargasso Sea and the problem of the eel's spawning site. *Naturwissenschaften* **82,** 32–36 (1995).

41. Helfman, G. S., Facey, D. E., Hales Jr., L. S. & Bozeman Jr., E. L. Reproductive ecology of the American eel. *Am. Fish. Soc. Symp.* **1,** 42–56 (1987).

42. Rousseau, K. *et al*. Long-term inhibitory effects of somatostatin and insulin-like growth factor 1 on growth hormone release by serum-free primary culture of pituitary cells from European eel (*Anguilla anguilla*). *Neuroendocrinology* **67,** 301–309 (1998).

43. Durif, C., Dufour, S. & Elie, P. The silvering process of *Anguilla anguilla*: A new classification from the yellow resident to the silver migrating stage. *J. Fish Biol.* **66,** 1025–1043 (2005).

44. Durif, C., Guibert, A. & Elie, P. Morphological discrimination of the silvering stages of the European eel. *Am. Fish. Soc. Symp.* **58,** 103–111 (2009).

45. Pankhurst, N. W. Relation of visual changes to the onset of sexual maturation in the European eel *Anguilla anguilla* (L.). *J. Fish Biol.* **21,** 127–140 (1982).

46. Aprahamian, M. W. Eel (*Anguilla anguilla* L.) production in the River Severn, England. *Pol. Arch. Hydrobiol.* **33,** 373–389 (1986).

47. Aprahamian, M. W. Age structure of eel, *Anguilla anguilla* (L.), populations in the River Severn, England, and the River Dee, Wales. *Aquacult. Fish. Manage.* **19,** 365–376 (1988).

48. Tesch, F. W. *The Eel* 1–71 (Blackwell Science, 2003).

49. Solomon, D. J. & Beach, M. H. Fish pass design for eel and elver (*Anguilla anguilla*). *R&D Technical Report W2-070/TR* (Environment Agency, 2004).

50. Kuwahara, A., Niimi, A. & Itagaki, H. Studies on a nematode parasitic in the air bladder of the eel. I. Description of *Anguillicola crassa* n. sp. (Philometridea, Anguillicolidae). *Jap. J. Parasit.* **23,** 275–279 (1974).

51. Peters, G. & Hartmann, F. *Anguillicola*, a parasitic nematode of the swim bladder spreading among eel populations in Europe. *Dis. Aquat. Organ.* **1,** 229–230 (1986).

52. Kennedy, C. R. & Fitch, D. J. Colonization, larval survival and epidemiology of the nematode *Anguillicola crassus*, parasitic in the eel, *Anguilla anguilla*, in Britain. *J. Fish Biol.* **36,** 117–131 (1990).

53. Hellström, A., Ljungberg, O. & Bornstein, S. *Anguillicola*, a new eel parasite in Sweden. *Sven. Veterinärtidn.* **40,** 211–213 (1988).

54. Moser, M. L., Patrick, W. S. & Crutchfield Jr., J. U. Infection of American eels, *Anguilla rostrata*, by an introduced nematode parasite, *Anguillicola crassus*, in North Carolina. *Copeia* **2001,** 848–853 (2001).

55. De Charleroy, D., Grisez, L., Thomas, K., Belpaire, C. & Ollevier, F. The life cycle of *Anguillicola crassus*. *Dis. Aquat. Organ.* **8,** 77–84 (1990).

56. Lehmitz, R. & Jürss, K. Hitzestabilität der Transketolase der Niere des Europäischen Aals (*Anguilla anguilla*). *Biol. Rundsch.* **15,** 61–63 (1977).

57. Banauch, D. *et al*. Eine Glucose-Dehydrogenase für die Glucose-Bestimmung in Körperflüssigkeiten. *Z. Klin. Chem. Klin. Biochem.* **13,** 101–107 (1975).

58. Bergmeyer, H. U. (ed.). *Methods of Enzymatic Analysis* Vol. 1 (Verlag Chemie, 1974).

59. Vanzetti, G. An azide-methemoglobin method for hemoglobin determination in blood. *J. Lab. Clin. Med.* **67,** 116–126 (1966).

60. Anonymous. ICES WKAREA Report 2009, Workshop on age reading of European and American eel (WKAREA). *ICES CM 2009\ACOM: 48* (2009).

61. Honeyfield, D. C. *et al*. Development of thiamine deficiencies and early mortality syndrome in lake trout by feeding experimental and feral fish diets containing thiaminase. *J. Aquat. Anim. Health* **17,** 4–12 (2005).

62. Ottinger, C. A., Honeyfield, D. C., Densmore, C. L. & Iwanowicz, L. R. Impact of thiamine deficiency on T-cell dependent and T-cell independent antibody production in lake trout. *J. Aquat. Anim. Health* **24,** 258–273 (2012).

63. Leonard, J. B. K., Norieka, J. F., Kynard, B. & McCormick, S. D. Metabolic rates in an anadromous clupeid, the American shad (*Alosa sapidissima*). *J. Comp. Physiol. B* **169,** 287–295 (1999).

64. Brett, J. R. The respiratory metabolism and swimming performance of young sockeye salmon. *J. Fish. Res. Board. Can.* **21,** 1183–1226 (1964).

65. Oliveira, K. & Hable, W. E. Artificial maturation, fertilization, and early development of the American eel (*Anguilla rostrata*). *Can. J. Zool.* **88,** 1121–1128 (2010).

66. Palstra, A. P., Cohen, E. G. H., Niemantsverdriet, P. R. W., van Ginneken, V. J. T. & van den Thillart, G. E. E. J. M. Artificial maturation and reproduction of European silver eel: Development of oocytes during final maturation. *Aquaculture* **249,** 533–547 (2005).

67. Fleming, A. Reproductive strategies of Atlantic salmon: Ecology and evolution. *Rev. Fish Biol. Fisher.* **6,** 379–416 (1996).

68. Jonsson, N., Jonsson, B. & Hansen, L. P. Changes in proximate composition and estimates of energetic costs during upstream migration and spawning in Atlantic salmon *Salmo salar*. *J. Anim. Ecol.* **66,** 425–436 (1997).

69. Jürss, K. & Tiemann, U. Einfluß von Thiaminpyrophosphat auf die Hitzestabilität der Transketolase der Niere von *Salmo gairdneri*. *Acta Biol. Med. Ger.* **32,** 691–693 (1974).

70. Amcoff, P. *et al*. Hepatic activities of thiamine-dependent enzymes, glucose-6 phosphate dehydrogenase and cytochrome P4501A in Baltic salmon (*Salmo salar*) yolk-sac fry after thiamine treatment. *Aquat. Toxicol.* **48,** 391–402 (2000).

71. Amcoff, P. *et al*. Physiological, biochemical and morphological studies of Baltic salmon yolk-sac fry with an experimental thiamine deficiency: Relations to the M74 syndrome. *Aquat. Toxicol.* **61,** 15–33 (2002).

72. Nash, R. D. M., Valencia, A. H. & Geffen, A. J. The origin of Fulton's condition factor—setting the record straight. *Fisheries* **31,** 236–238 (2006).

73. Pecora, L. J. & Highman, B. Organ weights and histology of chronically thiamine-deficient rats and their pair-fed controls. *J. Nutr.* **51,** 219–229 (1953).

74. Xu, Z.-P., Wawrousek, E. F. & Piatigorsky, J. Transketolase haploinsufficiency reduces adipose tissue and female fertility in mice. *Mol. Cell. Biol.* **22,** 6142–6147 (2002).

75. Brown, S. B., Honeyfield, D. C. & Vandenbyllaardt, L. Thiamine analysis in fish tissues. *Am. Fish. Soc. Symp.* **21,** 73–81 (1998).

76. Kankaanpää, H., Vuorinen, P. J., Sipiä, V. & Keinänen, M. Acute effects and bioaccumulation of nodularin in sea trout (*Salmo trutta* m. *trutta* L.) exposed orally to *Nodularia spumigena* under laboratory conditions. *Aquat. Toxicol.* **61,** 155–168 (2002).

77. Mancinelli, R. *et al*. Simultaneous liquid chromatographic assessment of thiamine, thiamine monophosphate and thiamine diphosphate in human erythrocytes: A study on alcoholics. *J. Chromatogr. B* **789,** 355–363 (2003).

78. Boqué, R. & Vander Heyden, Y. The limit of detection. *LC GC Eur.* **22,** 82–85 (2009).

79. Sylvander, P. & Bignert, A. Temporal variations in thiamine content of Baltic herring are associated with thiamine deficiency in Baltic Salmon, in Ph.D. thesis *Thiamine Dynamics in the Pelagic Food Web of the Baltic Sea*, Sylvander, P. (Stockholm University, 2013).

80. Li, X., Jenssen, E. & Fyhn, H. J. Effects of salinity on egg swelling in Atlantic salmon (*Salmo salar*). *Aquaculture* **76,** 317–334 (1989).

81. Rindi, G., de Giuseppe, L. & Ventura, U. Distribution and phosphorylation of oxythiamine in rat tissues. *J. Nutr.* **81,** 147–154 (1963).

82. Balaghi, M. & Pearson, W. N. Tissue and intracellular distribution of radioactive thiamine in normal and thiamine-deficient rats. *J. Nutr.* **89,** 127–132 (1966).

83. Batifoulier, F., Verny, M.-A., Besson, C., Demigne, C. & Remesy, C. Determination of thiamine and its phosphate esters in rat tissues analyzed as thiochromes on a RP-amide C16 column. *J. Chromatogr. B* **816,** 67–72 (2005).

84. Bettendorff, L., Lakaye, B., Kohn, G. & Wins, P. Thiamine triphosphate: A ubiquitous molecule in search of a physiological role. *Metab. Brain Dis.* **29,** 1069–1082 (2014).

85. Hansson, T. Comparison of two measures of missing cofactor in cofactor-dependent enzymes: Proportion versus relative increase. *Open Biomark. J.* **5,** 16–21; 10.2174/1875318301205010016 (2012).

86. Tate, R. & Nixon, P. F. Measurement of Michaelis constant for human erythrocyte transketolase and thiamin diphosphate. *Anal. Biochem.* **160,** 78–87 (1987).

87. Lowry, O. H., Rosebrough, N. J., Farr, A. L. & Randall, R. J. Protein measurement with the Folin phenol reagent. *J. Biol. Chem.* **193,** 265–275 (1951).

88. Lai, J. C. K. & Cooper, A. J. L. Brain α-ketoglutarate dehydrogenase complex: Kinetic properties, regional distribution, and effects of inhibitors. *J. Neurochem.* **47,** 1376–1386 (1986).

89. Lai, J. C. K. *et al*. Chronic hypoxia in development selectively alters the activities of key enzymes of glucose oxidative metabolism in brain regions. *Neurochem. Res.* **28,** 933–940 (2003).

90. Prough, R. A., Burke, M. D. & Mayer, R. T. in *Methods in Enzymology* Vol. LII, part C (eds. Fleischer, S. & Packer, L.) 372–377 (Academic Press, 1978).

91. Tamhane, C. & Dunlop, D. D. *Statistics and Data Analysis, From Elementary to Intermediate* 121–122 (Prentice Hall, 2000).

92. Sokal, R. R. & Rohlf, J. F. *Biometry, the Principles and Practice of Statistics in Biological Research* 541–549 (Freeman, 1995).

93. Sivertsen, E. Investigations of the relationship between narrowheaded and broadheaded eel and their nourishment. *Fisk. Dir. Skr. Ser. Hav. Unders.* **5,** 1–22 (1938).

94. Westerbom, M., Lappalainen, A. & Mustonen, O. Invariant size selection of blue mussels by roach despite variable prey size distributions. *Mar. Ecol. Prog. Ser.* **328,** 161–170 (2006).

95. Kautsky, N. On the trophic role of the blue mussel (*Mytilus edulis* L.) in a Baltic coastal ecosystem and the fate of the organic matter produced by the mussels. *Kieler Meeresforsch. Sonderh.* **5,** 454–461 (1981).

96. Jansson, A.-M. & Kautsky, N. in *Biology of Benthic Organisms, 11th European Symposium on Marine Biology, Galway, October 1976* (eds. Keegan, B. F., Ceidigh, P. O. & Boaden, P. J. S.) 359–366 (Pergamon Press Ltd., 1977).

97. Kautsky, U. & Kautsky, H. in *Biology and Ecology of Shallow Coastal Waters, Proceedings of the 28th European Marine Biology Symposium, 23rd–28th September 1993, Hersonissos, Crete, Greece* (eds. Eleftheriou, A., Ansell, A. D. & Smith, C. J.) 31–38 (Olsen & Olsen, 1995).

98. Koivisto, M. E. & Westerbom, M. Habitat structure and complexity as determinants of biodiversity in blue mussel beds on sublittoral rocky shores. *Mar. Biol.* **157,** 1463–1474 (2010).

99. Riisgård, H. U., Egede, P. P. & Barreiro Saavedra, I. Feeding behaviour of the mussel, *Mytilus edulis*: New observations, with a minireview of current knowledge. *J. Mar. Biol.* **2011,** 312459; 10.1155/2011/312459 (2011).

100. Sylvander, P., Häubner, N. & Snoeijs, P. The thiamine content of phytoplankton cells is affected by abiotic stress and growth rate. *Microb. Ecol.* **65,** 566–577 (2013).

101. Kautsky, H., Tobiasson, S. & Karlsson, J. in *The Sea 2011 – On the Environmental Status of Swedish Marine Areas* 36–41 (Swedish Institute for the Marine Environment, 2011). http://www.havsmiljoinstitutet.se/digitalAssets/1350/1350800_havet_2011.pdf.

102. Rosioru, D. M. Ecological resilience of *Mytilus galloprovincialis* from the Romanian Black Sea coast at environmental variations. *J. Environ. Prot. Ecol.* **15,** 924–932 (2014).

103. Beukema, J. J. Increased mortality in alternative bivalve prey during a period when the tidal flats of the Dutch Wadden Sea were devoid of mussels. *Neth. J. Sea Res.* **31,** 395–406 (1993).

104. Herlyn, M. & Millat, G. Decline of the intertidal blue mussel (*Mytilus edulis*) stock at the coast of Lower Saxony (Wadden Sea) and influence of mussel fishery on the development of young mussel beds. *Hydrobiologia* **426,** 203–210 (2000).

105. Beukema, J. J. & Dekker, R. Variability in annual recruitment success as a determinant of long-term and large-scale variation in annual production of intertidal Wadden Sea mussels (*Mytilus edulis*). *Helgol. Mar. Res.* **61,** 71–86 (2007).

106. Atkinson, P. W. *et al*. Changes in commercially fished shellfish stocks and shorebird populations in the Wash, England. *Biol. Conserv.* **114,** 127–141 (2003).

107. Brousseau, D. J., Goldberg, R. & Garza, C. Impact of predation by the invasive crab *Hemigrapsus sanguineus* on survival of juvenile blue mussels in western Long Island Sound. *Northeast. Nat.* **21,** 119–133 (2014).

108. Smith, J. R., Fong, P. & Ambrose, R. F. Dramatic declines in mussel bed community diversity: Response to climate change? *Ecology* **87,** 1153–1161 (2006).

109. Smith, J. R., Fong, P. & Ambrose, R. F. Long-term change in mussel (*Mytilus californianus* Conrad) populations along the wave-exposed coast of southern California. *Mar. Biol.* **149,** 537–545 (2006).

110. Broitman, B. R. *et al*. Spatial and temporal patterns of invertebrate recruitment along the west coast of the United States. *Ecol. Monogr.* **78,** 403-421 (2008).

111. Kurihara, T., Kosuge, T., Takami, H., Iseda, M. & Matsubara, K. Evidence of a sharper decrease in a non-indigenous mussel *Mytilus galloprovincialis* than in indigenous bivalves from 1978 to 2006 on Japanese rocky shores. *Biol. Invasions* **12,** 2671–2681 (2010).

112. Romanelli, M., Cordisco, C. A. & Giovanardi, O. The long-term decline of the *Chamelea gallina* L. (Bivalvia: Veneridae) clam fishery in the Adriatic Sea: Is a synthesis possible? *Acta Adriat.* **50,** 171–205 (2009).

113. Beukema, J. J., Dekker, R. & Jansen, J. M. Some like it cold: Populations of the tellinid bivalve *Macoma balthica* (L.) suffer in various ways from a warming climate. *Mar. Ecol. Prog. Ser.* **384,** 135–145 (2009).

114. Jonasson, J. P., Thorarinsdottir, G., Eiriksson, H., Solmundsson, J. & Marteinsdottir, G. Collapse of the fishery for Iceland scallop (*Chlamys islandica*) in Breidafjordur, West Iceland. *ICES J. Mar. Sci.* **64,** 298–308 (2007).

115. Barton, A., Hales, B., Waldbusser, G. G., Langdon, C. & Feely, R. A. The Pacific oyster, *Crassostrea gigas*, shows negative correlation to naturally elevated carbon dioxide levels:

Implications for near-term ocean acidification effects. *Limnol. Oceanogr.* **57,** 698–710 (2012).

116. Dyrynda, E. A. *et al*. Modulations in cell-mediated immunity of *Mytilus edulis* following the 'Sea Empress' oil spill. *J. Mar. Biol. Assoc. UK* **77,** 281–284 (1997).

117. Li, Y., Qin, J. G., Li, X. & Benkendorff, K. Assessment of metabolic and immune changes in postspawning Pacific oyster *Crassostrea gigas*: Identification of a critical period of vulnerability after spawning. *Aquacult. Res.* **41,** e155–e165; 10.1111/j.1365-2109.2010.02489.x (2010).

118. Pernet, F. *et al*. Mass mortalities of Pacific oysters *Crassostrea gigas* reflect infectious diseases and vary with farming practices in the Mediterranean Thau lagoon, France. *Aquacult. Environ. Interact.* **2,** 215–237 (2012).

119. Berthelin, C., Kellner, K. & Mathieu, M. Storage metabolism in the Pacific oyster (*Crassostrea gigas*) in relation to summer mortalities and reproductive cycle (West Coast of France). *J. Comp. Physiol. B* **125,** 359–369 (2000).

120. Kristmundsson, Á., Helgason, S., Bambir, S. H., Eydal, M. & Freeman, M. A. Previously unknown apicomplexan species infecting Iceland scallop, *Chlamys islandica* (Müller, 1776), queen scallop, *Aequipecten opercularis* L., and king scallop, *Pecten maximus* L. *J. Invertebr. Pathol.* **108,** 147–155 (2011).

121. Baba, K., Miyazono, A., Matsuyama, K., Kohno, S. & Kubota, S. Occurrence and detrimental effects of the bivalve-inhabiting hydroid *Eutima japonica* on juveniles of the Japanese scallop *Mizuhopecten yessoensis* in Funka Bay, Japan: Relationship to juvenile massive mortality in 2003. *Mar. Biol.* **151,** 1977–1987 (2007).

122. Samain, J.-F. Review and perspectives of physiological mechanisms underlying genetically-based resistance of the Pacific oyster *Crassostrea gigas* to summer mortality. *Aquat. Living Resour.* **24,** 227–236 (2011).

123. McCandless, D. W., Schenker, S. & Cook, M. Encephalopathy of thiamine deficiency: Studies of intracerebral mechanisms. *J. Clin. Invest.* **47,** 2268–2280 (1968).

124. Gibson, G. E., Ksiezak-Reding, H., Sheu, K.-F. R., Mykytyn, V. & Blass, J. P. Correlation of enzymatic, metabolic, and behavioral deficits in thiamin deficiency and its reversal. *Neurochem. Res.* **9,** 803–814 (1984).

125. Butterworth, R. F., Giguere, J.-F. & Besnard, A.-M. Activities of thiamine-dependent enzymes in two experimental models of thiamine-deficiency encephalopathy: 1. The pyruvate dehydrogenase complex. *Neurochem. Res.* **10,** 1417–1428 (1985).

126. Giguère, J.-F. & Butterworth, R. F. Activities of thiamine-dependent enzymes in two experimental models of thiamine deficiency encephalopathy: 3. Transketolase. *Neurochem. Res.* **12,** 305–310 (1987).

127. Borgna-Pignatti, C., Marradi, P., Pinelli, L., Monetti, N. & Patrini, C. Thiamine-responsive anemia in DIDMOAD syndrome. *J. Pediatr.* **114,** 405–410 (1989).

128. Rindi, G. *et al*. Thiamine transport by erythrocytes and ghosts in thiamine-responsive megaloblastic anaemia. *J. Inher. Metab. Dis.* **15,** 231–242 (1992).

129. Akinci, A., Teziç, T., Ertürk, G., Tarim, Ö. & Dalva, K. Thiamine-responsive megaloblastic anemia with diabetes mellitus and sensorineural deafness. *Acta Paediatr. Jpn.* **35,** 262–266 (1993).

130. Alston, T. A. & Abeles, R. H. Enzymatic conversion of the antibiotic metronidazole to an analog of thiamine. *Arch. Biochem. Biophys.* **257,** 357–362 (1987).

131. Diav-Citrin, O., Shechtman, S., Gotteiner, T., Arnon, J. & Ornoy, A. Pregnancy outcome after gestational exposure to metronidazole: A prospective controlled cohort study. *Teratology* **63,** 186–192 (2001).

132. Bellyei, S. *et al*. Cloning, sequencing, structural and molecular biological characterization of placental protein 20 (PP20)/human thiamin pyrophosphokinase (hTPK). *Placenta* **26,** 34–46 (2005).

133. Cooray, R. *et al*. Screening for microorganisms associated with M74 disease syndrome in sea-run Baltic salmon (*Salmo salar*). *Ambio* **28,** 77–81 (1999).

134. Elts, J. *et al*. Status and numbers of Estonian birds, 2003–2008. *Hirundo* **22,** 3–31 (2009).

135. Skov, H. *et al*. Waterbird populations and pressures in the Baltic Sea. *TemaNord 2011:550* (2011).

136. Ekroos, J. *et al*. Declines amongst breeding eider *Somateria mollissima* numbers in the Baltic/Wadden Sea flyway. *Ornis Fennica* **89,** 81–90 (2012).

137. Larsson, K. *et al*. Effects of an extensive *Prymnesium polylepis* bloom on breeding eiders in the Baltic Sea. *J. Sea Res.* **88,** 21–28 (2014).

138. Ekroos, J., Öst, M., Karell, P., Jaatinen, K. & Kilpi, M. Philopatric predisposition to predation-induced ecological traps: Habitat-dependent mortality of breeding eiders. *Oecologia* **170,** 979–986 (2012).

139. Jónsson, J. E. *et al*. Relationships between long-term demography and weather in a sub-arctic population of common eider. *PLoS ONE* **8,** e67093; 10.1371/journal.pone.0067093 (2013).

140. Garðarsson, A. Recent changes in numbers of cliff-breeding seabirds in Iceland. *Bliki* **27,** 13–22 (2006).

141. Garðarsson, A. Breeding success of kittiwake in Iceland in 2005. *Bliki* **27,** 23–26 (2006).

142. Bornaechea, P. G. & Garðarsson, A. A survey of the bird cliffs of Snæfellsnes, W-Iceland, in 2005. *Bliki* **27,** 51–54 (2006).

143. Wildemann, L., Böhm, M., Pabst, W. & Hess, B. Über die Beziehungen zwischen der Thiaminaufnahme und dem Gehalt an Thiamin, Thiaminpyrophosphat sowie Transketolaseaktivität in Rattenorganen. *Enzymol. Biol. Clin.* **10,** 81–112 (1969).

144. Ihara, H., Igarashi, H., Kakinoki, T., Hagane, Y. & Hashizume, N. Classification of hypo and hyperthiaminosis. *Int. J. Anal. Bio-Sci.* **1,** 45–49 (2013).

145. Parker, W. D. *et al*. Brain mitochondrial metabolism in experimental thiamine deficiency. *Neurology* **34,** 1477–1481 (1984).

146. Héroux, M. & Butterworth, R. F. Reversible alterations of cerebral γ-aminobutyric acid in pyrithiamine-treated rats: Implications for the pathogenesis of Wernicke's encephalopathy. *J. Neurochem.* **51,** 1221–1226 (1988).

147. Butterworth, R. F. & Héroux, M. Effect of pyrithiamine treatment and subsequent thiamine rehabilitation on regional cerebral amino acids and thiamine-dependent enzymes. *J. Neurochem.* **52,** 1079–1084 (1989).

148. Hildén, O. Ecology of duck populations in the island group of Valassaaret, Gulf of Bothnia. *Ann. Zool. Fenn.* **1,** 153–279 (1964).

149. Coulson, J. C. Variation in clutch size of the common eider: A study based on 41 breeding seasons on Coquet Island, Northumberland, England. *Waterbirds* **22,** 225–238 (1999).

150. Wrighton, S. A. *et al*. Modulation of rat hepatic cytochromes P450 by chronic methapyrilene treatment. *Biochem. Pharmacol.* **42,** 1093–1097 (1991).

151. Chen, H.-W. *et al*. The combined effects of garlic oil and fish oil on the hepatic antioxidant and drug-metabolizing enzymes of rats. *Brit. J. Nutr.* **89,** 189–200 (2003).

152. Takahashi, K. *et al*. Regulation of cytochrome b_5 expression by miR-223 in human liver: Effects on cytochrome P450 activities. *Pharmaceut. Res.* **31,** 780–794 (2014).

153. Whyte, J. J., Jung, R. E., Schmitt, C. J. & Tillitt, D. E. Ethoxyresorufin-*O*-deethylase (EROD) activity in fish as a biomarker of chemical exposure. *Crit. Rev. Toxicol.* **30,** 347–570 (2000).

154. van der Oost, R., Beyer, J. & Vermeulen, N. P. E. Fish bioaccumulation and biomarkers in environmental risk assessment: A review. *Environ. Toxicol. Pharmacol.* **13,** 57–149 (2003).

155. Gräns, J., Wassmur, B. & Celander, M. C. One-way inhibiting cross-talk between arylhydrocarbon receptor (AhR) and estrogen receptor (ER) signaling in primary cultures of rainbow trout hepatocytes. *Aquat. Toxicol.* **100,** 263–270 (2010).

156. Zamaratskaia, G., Rasmussen, M. K., Herbin, I., Ekstrand, B. & Zlabek, V. *In vitro* inhibition of porcine cytochrome P450 by 17β-estradiol and 17α-estradiol. *Interdiscip. Toxicol.* **4,** 78–84 (2011).

157. Norrgren, L., Andersson, T., Bergqvist, P. A. & Björklund, I. Chemical, physiological and morphological studies of feral Baltic salmon (*Salmo salar*) suffering from abnormal fry mortality. *Environ. Toxicol. Chem.* **12,** 2065–2075 (1993).

158. Wade, A. E., Wu, B. C., Holbrook, C. M. & Caster, W. O. Effects of thiamin antagonists on drug hydroxylation and properties of cytochrome P-450 in the rat. *Biochem. Pharmacol.* **22,** 1573–1580 (1973).

159. Grosse III, W. & Wade, A. E. The effect of thiamine consumption on liver microsomal drug-metabolizing pathways. *J. Pharmacol. Exp. Ther.* **176,** 758–765 (1971).

160. Omura, T. & Sato, R. The carbon monoxide-binding pigment of liver microsomes. I. Evidence for its hemoprotein nature. *J. Biol. Chem.* **239,** 2370–2378 (1964).

161. Ruchirawat, M., Mahathanatrakul, W., Sinrashatanant, Y. & Kittikool, D. Effects of thiamine deficiency on the metabolism and acute toxicity of dimethylnitrosamine in the rat. *Biochem. Pharmacol.* **27,** 1783–1786 (1978).

162. Yoo, J. S., Park, H. S., Ning, S. M., Lee, M. J. & Yang, C. S. Effects of thiamine deficiency on hepatic cytochromes P450 and drug-metabolizing enzyme activities. *Biochem. Pharmacol.* **39,** 519–525 (1990).

163. Wade, A. E., Greene, F. E., Ciordia, R. H., Meadows, J. S. & Caster, W. O. Effects of dietary thiamine intake on hepatic drug metabolism in the male rat. *Biochem. Pharmacol.* **18,** 2288–2292 (1969).

164. Galdhar, N. R. & Pawar, S. S. Hepatic drug metabolism and lipid peroxidation in thiamine deficient rats. *Int. J. Vitam. Nutr. Res.* **46,** 14–23 (1976).

165. Wade, A. E., Wu, B. & Lee, J. Nutritional factors affecting drug-metabolizing enzymes of the rat. *Biochem. Pharmacol.* **24,** 785–789 (1975).

166. Wade, A. E., Bunce, O. R., Harley, W., Baker, M. T. & Holmes, D. Effect of thiamin deprivation on the in vitro metabolic activation of benzo(a)pyrene. *Pharmacology* **29,** 142–148 (1984).

167. Wade, A. E., Evans, J. S., Holmes, D. & Baker, M. T. Influence of dietary thiamin on phenobarbital induction of rat hepatic enzymes responsible for metabolizing drugs and carcinogens. *Drug Nutr. Interact.* **2,** 117–130 (1983).

168. Ruchirawat, M., Aramphongphan, A., Tanphaichitr, V. & Bandittanukool, W. The effect of thiamine deficiency on the metabolism of acetaminophen (paracetamol). *Biochem. Pharmacol.* **30,** 1901–1906 (1981).

169. Sturludottir, E. *et al*. Spatial and temporal trends of contaminants in mussel sampled around the Icelandic coastline. *Sci. Total Environ.* **454–455,** 500–509 (2013).

170. Jörundsdóttir, H. Ó. *et al*. Pristine Arctic: Background mapping of PAHs, PAH metabolites and inorganic trace elements in the North-Atlantic Arctic and sub-Arctic coastal environment. *Sci. Total Environ.* **493,** 719–728 (2014).

171. Iber, H. *et al*. Modulation of drug metabolism in infectious and inflammatory diseases. *Drug Metab. Rev.* **31,** 29–41 (1999).

172. Renton, K. W. & Nicholson, T. E. Hepatic and central nervous system cytochrome P450 are down-regulated during lipopolysaccharide-evoked localized inflammation in brain. *J. Pharmacol. Exp. Ther.* **294,** 524–530 (2000).

173. Amcoff, P., Lundström, J., Teimert, L., Börjeson, H. & Norrgren, L. Physiological and morphological effects of microinjection of oxythiamine and PCBs in embryos of Baltic salmon (*Salmo salar*): A comparison with the M74 syndrome. *Ambio* **28,** 55–66 (1999).

174. Lundström, J. *et al*. Antioxidative systems, detoxifying enzymes and thiamine levels in Baltic salmon (*Salmo salar*) that develop M74. *Ambio* **28,** 24–29 (1999).

175. Nakajima, T. *et al*. Monoclonal antibody-directed characterization of benzene, ethoxyresorufin and pentoxyresorufin metabolism in rat liver microsomes. *Biochem. Pharmacol.* **40,** 1255–1261 (1990).

176. Kim, S. G., Reddy, S. L., States, J. C. & Novak, R. F. Pyridine effects on expression and molecular regulation of the cytochrome P450IA gene subfamily. *Mol. Pharmacol.* **40,** 52–57 (1991).

177. Kelley, R. W., Cheng, D. & Backes, W. L. Heteromeric complex formation between CYP2E1 and CYP1A2: Evidence for the involvement of electrostatic interactions. *Biochemistry* **45,** 15807–15816 (2006).

178. Foulon, V. *et al*. Purification, molecular cloning, and expression of 2-hydroxyphytanoyl-CoA lyase, a peroxisomal thiamine pyrophosphate-dependent enzyme that catalyzes the carbon-carbon bond cleavage during α-oxidation of 3-methyl-branched fatty acids. *Proc. Natl. Acad. Sci. USA* **96,** 10039–10044 (1999).

179. Sniekers, M. *et al*. Thiamine pyrophosphate: An essential cofactor for the α-oxidation in mammals – implications for thiamine deficiencies? *Cell. Mol. Life Sci.* **63,** 1553–1563 (2006).

180. Schönfeld, P. & Struy, H. Refsum disease diagnostic marker phytanic acid alters the physical state of membrane proteins of liver mitochondria. *FEBS Lett.* **457,** 179–183 (1999).

181. Wanders, R. J., Jansen, G. A. & Skjeldal, O. H. Refsum disease, peroxisomes and phytanic acid oxidation: A review. *J. Neuropathol. Exp. Neurol.* **60,** 1021–1031 (2001).

182. Brandt Busanello, E. N. *et al*. Disruption of mitochondrial homeostasis by phytanic acid in cerebellum of young rats. *Cerebellum* **12,** 362–369 (2013).

183. Okita, R. T. & Okita, J. R. Cytochrome P450 4A fatty acid omega hydroxylases. *Curr. Drug Metab.* **2,** 265–281 (2001).

184. Xu, F., Ng, V. Y., Kroetz, D. L. & Ortiz de Montellano, P. R. CYP4 isoform specificity in the ω-hydroxylation of phytanic acid, a potential route to elimination of the causative agent of Refsum's disease. *J. Pharmacol. Exp. Ther.* **318,** 835–839 (2006).

185. Blair, P. V. *et al*. Dietary thiamin level influences levels of its diphosphate form and thiamin-dependent enzymic activities of rat liver. *J. Nutr.* **129,** 641–648 (1999).

186. Berdanier, C. D., Tobin, R. B., Nielsen, R. C., Mehlman, M. A. & Veech, R. L. Effect of polychlorinated biphenyls and thiamin deficiency on liver metabolism in growing rats. *J. Toxicol. Environ. Health* **1,** 91–105 (1975).

187. Brink, A. J., Lochner, A. & Lewis, C. M. Thiamine deficiency and beriberi heart disease. *S. Afr. Med. J.* **40,** 581–590 (1966).

188. Swank, R. L. Avian thiamin deficiency: A correlation of the pathology and clinical behavior. *J. Exp. Med.* **71,** 683–702 (1940).

189. Gries, C. L. & Scott, M. L. The pathology of thiamin, riboflavin, pantothenic acid and niacin deficiencies in the chick. *J. Nutr.* **102,** 1269–1285 (1972).

190. Holz, P. H., Phelan, J. R. & Slocombe, R. Thiamine deficiency in honeyeaters. *J. Avian Med. Surg.* **16,** 21–25 (2002).

191. Jubb, K. V. F. & Huxtable, C. R. in *Pathology of Domestic Animals* Vol. 1 (eds. Jubb, K. V. F., Kennedy, P. C. & Palmer, N.) 343–344 (Academic Press, 1993).

192. Saab, C. Y. & Willis, W. D. The cerebellum: Organization, functions and its role in nociception. *Brain Res. Rev.* **42,** 85–95 (2003).

193. Husband, S. & Shimizu, T. Evolution of the Avian Visual System, in *Avian Visual Cognition* (ed. Cook, R. G.) (On-line, 2001). http://www.pigeon.psy.tufts.edu/avc/husband/.

194. Schmahmann, J. D. in *The Cerebellum and Its Disorders* (eds. Manto, M.-U. & Pandolfo, M.) 137 (Cambridge University Press, 2002).

195. Mörner, T., Carlsson, L., Hägerroth, P.-Å. & Balk, L. abstract 359 presented at the Joint 61st WDA/10th Biennial EWDA Conference "Convergence in Wildlife Health", Lyon, 23–27 July, 2012.

196. Mörner, T. *et al*. abstract P25 presented at the 62nd International Conference of the Wildlife Disease Association, Knoxville, Tennessee, July 27–August 2, 2013.

197. Aoyama, J. Life history and evolution of migration in catadromous eels (genus *Anguilla*). *Aqua-BioSci. Monogr. (ABSM)* **2,** 1–42 (2009).

198. Dekker, W. & Casselman, J. M. (coordinators). The 2003 Québec declaration of concern about eel declines—11 years later: Are eels climbing back up the slippery slope? *Fisheries* **39,** 613–614 (2014).

199. Dekker, W. in *Eel Biology* (eds. Aida, K., Tsukamoto, K. & Yamauchi, K.) 237–254 (Springer, 2003).

200. McCarthy, T. K. *et al*. Long-term effects of hydropower installations and associated river regulation on River Shannon eel populations: Mitigation and management. *Hydrobiologia* **609,** 109–124 (2008).

201. Kimura, S., Inoue, T. & Sugimoto, T. Fluctuation in the distribution of low-salinity water in the North Equatorial Current and its effect on the larval transport of the Japanese eel. *Fish. Oceanogr.* **10,** 51–60 (2001).

202. Knights, B. A review of the possible impacts of long-term oceanic and climate changes and fishing mortality on recruitment of anguillid eels of the Northern Hemisphere. *Sci. Total Environ.* **310,** 237–244 (2003).

203. Palstra, A. P., van Ginneken, V. J. T., Murk, A. J. & van den Thillart, G. E. E. J. M. Are dioxin-like contaminants responsible for the eel (*Anguilla anguilla*) drama? *Naturwissenschaften* **93,** 145–148 (2006).

204. Castonguay, M. *et al*. Why is recruitment of the American eel, *Anguilla rostrata*, declining in the St. Lawrence River and Gulf? *Can. J. Fish. Aquat. Sci.* **51,** 479–488 (1994).

205. Hagström, O. & Wickström, H. Immigration of young eels to the Skagerrak-Kattegat area 1900 to 1989. *Int. Rev. Ges. Hydrobiol.* **75,** 707–716 (1990).

206. Tsukamoto, K. Spawning of eels near a seamount. *Nature* **439,** 929 (2006).

207. Tsukamoto, K. *et al*. Oceanic spawning ecology of freshwater eels in the western North Pacific. *Nat. Commun.* **2,** 179; 10.1038/ncomms1174 (2011).

208. Amcoff, P., Börjeson, H., Eriksson, R. & Norrgren, L. Effects of thiamine treatments on survival of M74-affected feral Baltic salmon. *Am. Fish. Soc. Symp.* **21,** 31–40 (1998).

209. Brown, S. B. *et al*. Effectiveness of egg immersion in aqueous solutions of thiamine and thiamine analogs for reducing early mortality syndrome. *J. Aquat. Anim. Health* **17,** 106–112 (2005).

210. Natarajan, K. V. Distribution of thiamine, biotin, and niacin in the sea. *Appl. Microbiol.* **16,** 366–369 (1968).

211. Natarajan, K. V. Distribution and significance of vitamin B_{12} and thiamine in the subarctic Pacific Ocean. *Limnol. Oceanogr.* **15,** 655–659 (1970).

212. Ohwada, K. & Taga, N. Distribution and seasonal variation of vitamin B_{12}, thiamine and biotin in the sea. *Mar. Chem.* **1,** 61–73 (1972).

213. Nishijima, T. & Hata, Y. Distribution of vitamin B_{12}, thiamine, and biotin in the water of Harima-Nada Sea. *B. Jpn. Soc. Sci. Fish.* **52,** 1533–1545 (1986).

214. von Cleménçon, H. Verbreitung einiger B-Vitamine in Kleingewässern aus der Umgebung von Bern und Untersuchungen über die Abgabe von B-vitaminen durch Algen. *Schweiz. Z. Hydrol.* **25,** 157–165 (1963).

215. Hutchinson, G. E. *A Treatise on Limnology* Vol. 2 306–354 (Wiley, 1967).

216. Ohwada, K. & Taga, N. Vitamin B_{12}, thiamine, and biotin in Lake Sagami. *Limnol. Oceanogr.* **17,** 315–320 (1972).

217. Ohwada, K., Otsuhata, M. & Taga, N. Seasonal cycles of vitamin B_{12}, thiamine and biotin in the surface water of Lake Tsukui. *B. Jpn. Soc. Sci. Fish.* **38,** 817–823 (1972).

218. Tanaka, H., Kagawa, H. & Ohta, H. Production of leptocephali of Japanese eel (*Anguilla japonica*) in captivity. *Aquaculture* **201,** 51–60 (2001).

219. Hashimoto, Y., Arai, S. & Nose, T. Thiamine deficiency symptoms experimentally induced in the eel. *B. Jpn. Soc. Sci. Fish.* **36,** 791–797 (1970).

220. Arai, S., Nose, T. & Hashimoto, Y. Qualitative requirements of young eels *Anguilla japonica* for water-soluble vitamins and their deficiency symptoms. *Bull. Freshwater Fish. Res. Lab.* **22,** 69–83 (1972).

221. Kreutzmann, H.-L. & Lehmitz, R. Der Einfluß von Vitamin-B_1-Mangel, auf physiologische und morphologische Blutparameter, bei Aalen (*Anguilla anguilla*). *Arch. Tierernähr.* **26,** 293–303 (1976).

222. Boon, J. H., Lokin, C. J. A., Ceusters, R. & Ollevier, F. Some properties of the blood of European eel (*Anguilla anguilla*) and the possible relationship with *Anguillicola crassus* infestations. *Aquaculture* **76,** 203–208 (1989).

223. Höglund, J., Andersson, J. & Härdig, J. Haematological responses in the European eel, *Anguilla anguilla* L., to sublethal infestation by *Anguillicola crassus* in a thermal effluent of the Swedish Baltic. *J. Fish Dis.* **15,** 507–514 (1992).

224. Kelly, C. E., Kennedy, C. R. & Brown, J. A. Physiological status of wild European eels (*Anguilla anguilla*) infected with the parasitic nematode, *Anguillicola crassus*. *Parasitology* **120,** 195–202 (2000).

225. Palstra, A. P., Heppener, D. F. M., van Ginneken, V. J. T., Székely, C. & van den Thillart, G. E. E. J. M. Swimming performance of silver eels is severely impaired by the swim-bladder parasite *Anguillicola crassus*. *J. Exp. Mar. Biol. Ecol.* **352,** 244–256 (2007).

226. Boon, J. H. *et al*. The effect of different infection levels with infective larvae of *Anguillicola crassus* on haematological parameters of European eel (*Anguilla anguilla*). *Aquaculture* **87,** 243–253 (1990).

227. Tiemann, U. & Lehmitz, R. Frühdiagnose eines Vitamin-B_1-Mangels bei der Regenbogenforelle (*Salmo gairdneri*) und beim Aal (*Anguilla anguilla*). *Z. Binnenfisch. DDR* **10,** 312–319 (1975).

228. Balk, L., Meijer, J., DePierre, J. W. & Appelgren, L.-E. The uptake and distribution of [^{3}H]benzo[*a*]pyrene in the northern pike (*Esox lucius*). Examination by whole-body autoradiography and scintillation counting. *Toxicol. Appl. Pharmacol.* **74,** 430–449 (1984).

229. Egginton, S. Metamorphosis of the American eel, *Anguilla rostrata* Le Seur: II. Structural reorganisation of the locomotory musculature. *J. Exp. Zool.* **238,** 297–309 (1986).

230. Brækkan, O. R. Function of the red muscle in fish. *Nature* **178,** 747–748 (1956).

231. Hulbert, W. C. Ph.D. thesis *The Functional Role of Red and White Muscle in the Eel,* Anguilla rostrata*: An Ultrastructural and Enzyme Study*. (University of Ottawa, 1976).

232. Hulbert, W. C. & Moon, T. W. The potential for lactate utilization by red and white muscle of the eel *Anguilla rostrata* L. *Can. J. Zool.* **56,** 128–135 (1978).

233. Boström, S.-L. & Johansson, R. G. Enzyme activity patterns in white and red muscle of the eel (*Anguilla anguilla*) at different developmental stages. *Comp. Biochem. Physiol. B* **42,** 533–542 (1972).

234. Fitzsimons, J. D. *et al*. Impacts of diet on thiamine status of Lake Ontario American eels. *Trans. Am. Fish. Soc.* **142,** 1358–1369 (2013).

235. Brækkan, O. R. A comparative study of vitamins in the trunk muscles of fishes. *Fisk. Dir. Skr. Ser. Teknol. Unders.* **3,** 1–42 (1959).

236. Koski, P., Soivio, A., Hartikainen, K., Hirvi, T. & Myllylä, T. M74 syndrome and thiamine in salmon broodfish and offspring. *Boreal Environ. Res.* **6,** 79–92 (2001).

237. Ohta, H., Kagawa, H., Tanaka, H., Okuzawa, K. & Hirose, K. Changes in fertilization and hatching rates with time after ovulation induced by 17,20*β*-dihydroxy-4-pregnen-3-one in the Japanese eel, *Anguilla japonica*. *Aquaculture* **139,** 291–301 (1996).

238. Ohta, H. *et al*. Artificial induction of maturation and fertilization in the Japanese eel, *Anguilla japonica*. *Fish Physiol. Biochem.* **17,** 163–169 (1997).

239. Kagawa, H. in *Eel Biology* (eds. Aida, K., Tsukamoto, K. & Yamauchi, K.) 401–414 (Springer, 2003).

240. Kagawa, H., Tanaka, H., Ohta, H., Unuma, T. & Nomura, K. The first success of glass eel production in the world: Basic biology on fish reproduction advances new applied technology in aquaculture. *Fish Physiol. Biochem.* **31,** 193–199 (2005).

241. Ellerby, D. J., Spierts, I. L. Y. & Altringham, J. D. Slow muscle power output of yellow- and silver-phase European eels (*Anguilla anguilla* L.): Changes in muscle performance prior to migration. *J. Exp. Biol.* **204,** 1369–1379 (2001).

242. Ketola, H. G. *et al*. Thiamine status of Cayuga Lake rainbow trout and its influence on spawning migration. *N. Am. J. Fish. Manage.* **25,** 1281–1287 (2005).

243. Fitzsimons, J. D. *et al*. The effect of thiamine injection on upstream migration, survival, and thiamine status of putative thiamine-deficient coho salmon. *J. Aquat. Anim. Health* **17,** 48–58 (2005).

244. Thurow, F. Über Fangerträge und Wachstum des Aales in der westlichen Ostsee. *Z. Fischerei Hilfswiss.* **8,** 597–626 (1959).

245. Larsson, P., Hamrin, S. & Okla, L. Fat content as a factor inducing migratory behavior in the eel (*Anguilla anguilla* L.) to the Sargasso Sea. *Naturwissenschaften* **77,** 488–490 (1990).

246. van Ginneken, V. J. T. & van den Thillart, G. E. E. J. M. Eel fat stores are enough to reach the Sargasso. *Nature* **403,** 156–157 (2000).

247. Belpaire, C. G. J. *et al*. Decreasing eel stocks: Survival of the fattest? *Ecol. Freshwater Fish* **18,** 197–214 (2009).

248. Williams Jr., J. N. & Anderson, C. E. Effect of thiamine deficiency and thiamine injection on total liver lipids, phospholipid, plasmalogen and cholesterol in the rat. *J. Nutr.* **69,** 229–234 (1959).

249. Kather, H., Rivera, M. & Brand, K. Interrelationship and control of glucose metabolism and lipogenesis in isolated fat-cells. Effect of the amount of glucose uptake on the rates of the pentose phosphate cycle and of fatty acid synthesis. *Biochem. J.* **128,** 1089–1096 (1972).

250. Bubber, P., Ke, Z.-J. & Gibson, G. E. Tricarboxylic acid cycle enzymes following thiamine deficiency. *Neurochem. Int.* **45,** 1021–1028 (2004).

251. Mkrtchyan, G. *et al*. Molecular mechanisms of the non-coenzyme action of thiamin in brain: Biochemical, structural and pathway analysis. *Sci. Rep.* **5,** 12583; 10.1038/srep12583 (2015).

252. Österblom, H., Bignert, A., Fransson, T. & Olsson, O. A decrease in fledging body mass in common guillemot *Uria aalge* chicks in the Baltic Sea. *Mar. Ecol. Prog. Ser.* **224,** 305–309 (2001).

253. Bignert, A. *et al*. *Comments Concerning the National Swedish Contaminant Monitoring Programme in Marine Biota, 2014* (Report No. 1:2014, Swedish Museum of Natural History, 2014).

254. Faxneld, S. *et al*. *Biological Effects and Environmental Contaminants in Herring and Baltic Sea Top Predators* (Report No. 6:2014, Swedish Museum of Natural History, 2014).

255. Balbontín, J. *et al*. Lifetime individual plasticity in body condition of a migratory bird. *Biol. J. Linn. Soc.* **105,** 420–434 (2012).

256. Rioux Paquette, S., Pelletier, F., Garant, D. & Bélisle, M. Severe recent decrease of adult body mass in a declining insectivorous bird population. *Proc. R. Soc. B* **281,** 20140649; 10.1098/rspb.2014.0649 (2014).

257. Erlandson-Hammargren, E. & Pedersen, B. *Mörrum* 32 (Piscator, 1998).

258. Fitzsimons, J. D. The effect of B-vitamins on a swim-up syndrome in Lake Ontario lake trout. *Suppl. J. Great Lakes Res.* **21,** 286–289 (1995).

259. McDonald, G., Fitzsimons, J. D. & Honeyfield, D. C. (eds.). Early life stage mortality syndrome in fishes of the Great Lakes and Baltic Sea, *American Fisheries Society, Symposium 21* (1998).

260. Bengtsson, B.-E., Hill, C., Nellbring, S. & Kessler, E. (eds.). Reproductive disturbances in Baltic Sea fish: An international perspective, *Ambio 28(1)* (1999).

261. Blazer, V. S. & Brown, L. L. (eds.). Early mortality syndrome in Great Lakes salmonines, *Journal of Aquatic Animal Health 17(1)* (2005).

262. Amcoff, P., Norrgren, L., Börjeson, H. & Lindeberg, J. in *Report From the Second Workshop on Reproduction Disturbances in Fish 20–23 November 1995, Högberga Gård, Lidingö, Stockholm, Sweden* (eds. Bengtsson, B.-E., Hill, C. & Nellbring, S.) 38–39 (Report 4534, Swedish Environmental Protection Agency, 1996).

263. Koski, P., Pakarinen, M. & Soivio, A. in *Report From the Second Workshop on Reproduction Disturbances in Fish 20–23 November 1995, Högberga Gård, Lidingö, Stockholm, Sweden* (eds. Bengtsson, B.-E., Hill, C. & Nellbring, S.) 46 (Report 4534, Swedish Environmental Protection Agency, 1996).

264. Marcquenski, S. V. in *Report From the Second Workshop on Reproduction Disturbances in Fish 20–23 November 1995, Högberga Gård, Lidingö, Stockholm, Sweden* (eds. Bengtsson, B.-E., Hill, C. & Nellbring, S.) 73–75 (Report 4534, Swedish Environmental Protection Agency, 1996).

265. Fitzsimons, J. & Brown, S. in *Report From the Second Workshop on Reproduction Disturbances in Fish 20–23 November 1995, Högberga Gård, Lidingö, Stockholm, Sweden* (eds. Bengtsson, B.-E., Hill, C. & Nellbring, S.) 76–78 (Report 4534, Swedish Environmental Protection Agency, 1996).

266. Hornung, M. W., Miller, L., Peterson, R. E., Marcquenski, S. & Brown, S. in *Report From the Second Workshop on Reproduction Disturbances in Fish 20–23 November 1995, Högberga Gård, Lidingö, Stockholm, Sweden* (eds. Bengtsson, B.-E., Hill, C. & Nellbring, S.) 82–83 (Report 4534, Swedish Environmental Protection Agency, 1996).

267. Brown, S., Palace, V., Klaverkamp, J. & Fitzsimons, J. in *Report From the Second Workshop on Reproduction Disturbances in Fish 20–23 November 1995, Högberga Gård, Lidingö, Stockholm, Sweden* (eds. Bengtsson, B.-E., Hill, C. & Nellbring, S.) 84–85 (Report 4534, Swedish Environmental Protection Agency, 1996).

268. Amcoff, P., Börjeson, H., Landergren, P., Vallin, L. & Norrgren, L. Thiamine (vitamin B_1) concentrations in salmon (*Salmo salar*), brown trout (*Salmo trutta*) and cod (*Gadus morhua*) from the Baltic Sea. *Ambio* **28,** 48–54 (1999).

269. Brown, S. & Honeyfield, D. *Early Mortality Syndrome Research and Information Coordination Meetings* (2004 Project Progress Report, Great Lakes Fishery Commission, 2004).

270. Riley, S. C., Rinchard, J., Honeyfield, D. C., Evans, A. N. & Begnoche, L. Increasing thiamine concentrations in lake trout eggs from Lakes Huron and Michigan coincide with low alewife abundance. *N. Am. J. Fish. Manage.* **31,** 1052–1064 (2011).

271. Fitzsimons, J. D. *et al*. Use of stable isotopes to identify redds of putative hatchery and wild Atlantic salmon and evaluate their spawning habitat and egg thiamine status in a Lake Ontario tributary. *N. Am. J. Fish. Manage.* **33,** 741–753 (2013).

272. Fitzsimons, J. D. *et al*. Influence of thiamine deficiency on lake trout larval growth, foraging, and predator avoidance. *J. Aquat. Anim. Health* **21,** 302–314 (2009).

273. Ketola, H. G. *et al*. Effect of thiamine status on probability of Lake Ontario Chinook salmon spawning in the upper or lower sections of Salmon River, New York. *N. Am. J. Fish. Manage.* **29,** 895–902 (2009).

274. Carvalho, P. S. M. *et al*. Thiamine deficiency effects on the vision and foraging ability of lake trout fry. *J. Aquat. Anim. Health* **21,** 315–325 (2009).

275. Ottinger, C. A., Honeyfield, D. C., Densmore, C. L. & Iwanowicz, L. R. *In vitro* immune functions in thiamine-replete and -depleted lake trout (*Salvelinus namaycush*). *Fish Shellfish Immun.* **38,** 211–220 (2014).

276. Lee, B.-J., Jaroszewska, M., Dabrowski, K., Czesny, S. & Rinchard, J. Effects of vitamin B_1 (thiamine) deficiency in lake trout alevins and preventive treatments. *J. Aquat. Anim. Health* **21,** 290–301 (2009).

277. Sato, M., Yoshinaka, R., Kuroshima, R., Morimoto, H. & Ikeda, S. Changes in water soluble vitamin contents and transaminase activity of rainbow trout egg during development. *Nippon Suisan Gakk.* **53,** 795–799 (1987).

278. Brown, S. B., Fitzsimons, J. D., Palace, V. P. & Vandenbyllaardt, L. Thiamine and early mortality syndrome in lake trout. *Am. Fish. Soc. Symp.* **21,** 18–25 (1998).

279. Fisher, J. P., Brown, S. B., Wooster, G. W. & Bowser, P. R. Maternal blood, egg and larval thiamin levels correlate with larval survival in landlocked Atlantic salmon (*Salmo salar*). *J. Nutr.* **128,** 2456–2466 (1998).

280. Roth-Maier, D. A., Kirchgessner, M. & Rajtek, S. Retention and utilization of thiamin by gravid and non gravid rats with varying dietary thiamin supply. *Int. J. Vitam. Nutr. Res.* **60,** 343–350 (1990).

281. Roth-Maier, D. A., Trübswetter, N., Stangl, G. I. & Kirchgessner, M. Dietary thiamin supply during lactation influences thiamin status in lactating rats and their offspring and the thiamin level in milk. *Z. Ernährungswiss.* **36,** 169–175 (1997).

282. Fynn-Aikins, K., Bowser, P. R., Honeyfield, D. C., Fitzsimons, J. D. & Ketola, H. G. Effect of dietary amprolium on tissue thiamin and Cayuga syndrome in Atlantic salmon. *Trans. Am. Fish. Soc.* **127,** 747–757 (1998).

283. Polin, D., Ott, W. H., Wynosky, E. R. & Porter, C. C. Estimation of thiamine requirement for optimum hatchability from the relationship between dietary and yolk levels of the vitamin. *Poultry Sci.* **42,** 925–928 (1963).

284. Blass, J. P., Piacentini, S., Boldizsar, E. & Baker, A. Kinetic studies of mouse brain transketolase. *J. Neurochem.* **39,** 729–733 (1982).

285. Salminen, M. in Joint Meeting on Causes of Marine Mortality of Salmon in the North Pacific and North Atlantic Oceans and in the Baltic Sea, March 14–15, 2002, Vancouver, British Columbia, Canada. *Technical Report No. 4* 27–29 (NPAFC, 2002).

286. Jonsson, N., Jonsson, B. & Hansen, L. P. The marine survival and growth of wild and hatchery-reared Atlantic salmon. *J. Appl. Ecol.* **40,** 900–911 (2003).

287. Friedland, K. D., Reddin, D. G. & Castonguay, M. Ocean thermal conditions in the post-smolt nursery of North American Atlantic salmon. *ICES J. Mar. Sci.* **60,** 343–355 (2003).

288. Friedland, K. D. *et al*. The recruitment of Atlantic salmon in Europe. *ICES J. Mar. Sci.* **66,** 289–304 (2009).

289. Mäntyniemi, S. *et al*. Both predation and feeding opportunities may explain changes in survival of Baltic salmon post-smolts. *ICES J. Mar. Sci.* **69,** 1574–1579 (2012).

290. Karlström, Ö. Development of the M74 syndrome in wild populations of Baltic salmon (*Salmo salar*) in Swedish rivers. *Ambio* **28,** 82–86 (1999).

291. Honeyfield, D. C., Murphy, J. M., Howard, K. G., Strasburger, W. W. & Matz, A. An exploratory assessment of thiamine status in Western Alaska chinook salmon (*Onchorhynchus tshawytscha*). *North Pac. Anadromous Fish Comm. Bull.* **6,** in press (2016).

292. Anonymous. ICES WGBAST Report 2014, Report of the Baltic salmon and trout assessment working group (WGBAST). *ICES CM 2014/ACOM:08* (2014).

293. Stacey, N. E. & Hourston, A. S. Spawning and feeding behavior of captive Pacific herring, *Clupea harengus pallasi*. *Can. J. Fish. Aquat. Sci.* **39,** 489–498 (1982).

294. Nøttestad, L. *et al*. Schooling dynamics of Norwegian spring spawning herring (*Clupea harengus* L.) in a coastal spawning area. *Sarsia* **80,** 277–284 (1996).

295. Dalpadado, P., Ellertsen, B., Melle, W. & Dommasnes, A. Food and feeding conditions of Norwegian spring-spawning herring (*Clupea harengus*) through its feeding migrations. *ICES J. Mar. Sci.* **57,** 843–857 (2000).

296. Laine, P., Eklund, J. & Soikkeli, M. Reproduction of Baltic herring after dissimilar growth and overwintering seasons. *Ophelia* **48,** 155–165 (1998).

297. Guillemette, M. The effect of time and digestion constraints in common eiders while feeding and diving over blue mussel beds. *Funct. Ecol.* **12,** 123–131 (1998).

298. Nissling, A. & Westin, L. Egg buoyancy of Baltic cod (*Gadus morhua*) and its implications for cod stock fluctuations in the Baltic. *Mar. Biol.* **111,** 33–35 (1991).

299. Nissling, A., Larsson, R., Vallin, L. & Frohlund, K. Assessment of egg and larval viability in cod, *Gadus morhua*: Methods and results from an experimental study. *Fish. Res.* **38,** 169–186 (1998).

300. Frommel, A. Y., Schubert, A., Piatkowski, U. & Clemmesen, C. Egg and early larval stages of Baltic cod, *Gadus morhua*, are robust to high levels of ocean acidification. *Mar. Biol.* **160,** 1825–1834 (2013).

301. Åkerman, G. & Balk, L. Descriptive studies of mortality and morphological disorders in early life stages of cod and salmon originating from the Baltic Sea. *Am. Fish. Soc. Symp.* **21,** 41–61 (1998).

302. Chalupnicki, M. A. *et al*. Thiamine status and culture of rainbow smelt (*Osmerus mordax*) from Owasco Lake, New York. *J. Freshwater Ecol.* **25,** 211–217 (2010).

303. Chalupnicki, M. A., Ketola, H. G., Zehfus, M. H., Crosswait, J. R. & Rinchard, J. Thiamine status of rainbow smelt (*Osmerus mordax*) eggs in the Great Lakes, USA. *J. Freshwater Ecol.* **27,** 31–39 (2012).

304. Bailey, M. M. Age, growth, maturity, and sex composition of the American smelt, *Osmerus mordax* (Mitchill), of western Lake Superior. *Trans. Am. Fish. Soc.* **93,** 382–395 (1964).

305. Cooper, J. E. Identification of eggs, larvae, and juveniles of the rainbow smelt, *Osmerus mordax*, with comparisons to larval alewife, *Alosa pseudoharengus*, and gizzard shad, *Dorosoma cepedianum*. *Trans. Am. Fish. Soc.* **107,** 56–62 (1978).

306. McKenzie, R. A. Smelt life history and fishery in the Miramichi River, New Brunswick. *Bull. Fish. Res. Board Can.* **144,** 1–77 (1964).

307. Hoover, E. E. The spawning activities of fresh water smelt, with special reference to the sex ratio. *Copeia* **1936,** 85–91 (1936).

308. Riley, S. C. *et al*. Thiamine concentrations in lake whitefish eggs from the upper Great Lakes are related to maternal diet. *J. Great Lakes Res.* **37,** 732–737 (2011).

309. Muir, A. M. *et al*. Reproductive life-history strategies in lake whitefish (*Coregonus clupeaformis*) from the Laurentian Great Lakes. *Can. J. Fish. Aquat. Sci.* **71,** 1256–1269 (2014).

310. Hart, J. L. The spawning and early life history of the whitefish, *Coregonus clupeaformis* (Mitchill), in the Bay of Quinte, Ontario. *Contrib. Can. Biol. Fish.* **6,** 165–214 (1931).

311. Brown, R. W. & Taylor, W. W. Effects of egg composition and prey density on the larval growth and survival of lake whitefish (*Coregonus clupeaformis* Mitchill). *J. Fish Biol.* **40,** 381–394 (1992).

312. Morrow, J. E. *The Freshwater Fishes of Alaska* 38 (Alaska Northwest Publishing Company, 1980).

313. Marcquenski, S. V. & Brown, S. B. in *Chemically induced alterations in functional development and reproduction of fishes* (eds. Rolland, R. M., Gilbertson, M. & Peterson, R. E.) 135–152 (SETAC Press, 2005).

314. Fitzsimons, J. D. & Brown, S. B. Reduced egg thiamine levels in inland and Great Lakes lake trout and their relationship with diet. *Am. Fish. Soc. Symp.* **21,** 160–171 (1998).

315. Brown, S. B. *et al*. Thiamine status in adult salmonines in the Great Lakes. *J. Aquat. Anim. Health* **17,** 59–64 (2005).

316. Honeyfield, D. C., Fynn-Aikins, K., Fitzsimons, J. D. & Mota, J. A. Effect of dietary amprolium on egg and tissue thiamine concentrations in lake trout. *Am. Fish. Soc. Symp.* **21,** 172–177 (1998).

317. Honeyfield, D. C., Hnath, J. G., Copeland, J., Dabrowski, K. & Blom, J. H. Correlation of nutrients and environmental contaminants in Lake Michigan coho salmon with incidence of early mortality syndrome. *Am. Fish. Soc. Symp.* **21,** 135–145 (1998).
